# Indium-Mediated Acyloxyallylation-Based Synthesis of *Galacto*-Configured Higher-Carbon Sugar Alcohols as Potential Phase Change Materials

**DOI:** 10.1021/acs.joc.4c00067

**Published:** 2024-04-05

**Authors:** Nina Biedermann, Julian Schnizer, Daniel Lager, Michael Schnürch, Christian Stanetty

**Affiliations:** † Institute of Applied Synthetic Chemistry, TU Wien, Getreidemarkt 9/163, 1060 Vienna, Austria; ‡ Energy Department, AIT Austrian Institute of Technology GmbH, Giefinggasse 2, 1210 Vienna, Austria

## Abstract

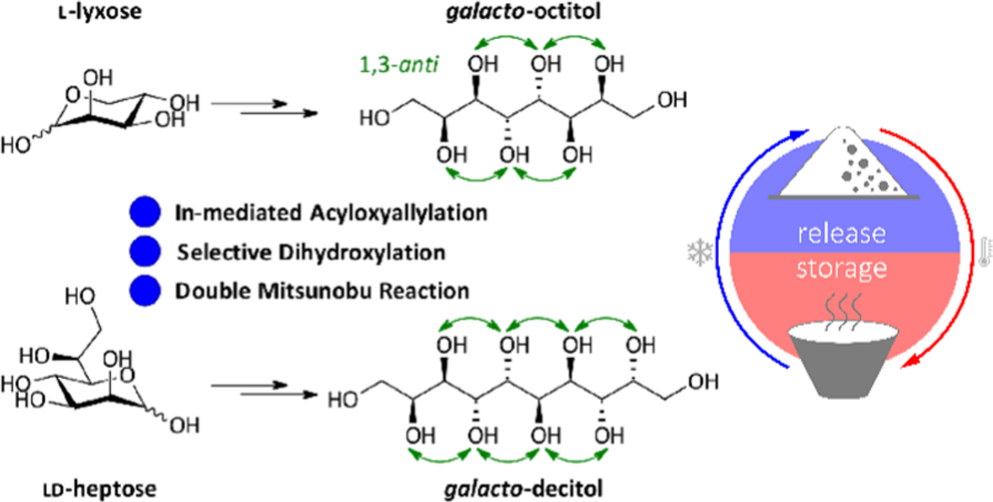

Sugar alcohols fulfilling specific structural requirements are a substance class with great potential as organic phase change materials (PCMs). Within this work, we demonstrate the indium-mediated acyloxyallylation (IMA) as a useful strategy for the synthesis of higher-carbon sugar alcohols of the *galacto*-family featuring all hydroxyl groups in a 1,3-*anti*-relationship with three major synthetic achievements: first, the dihydroxylation of the IMA-derived allylic sugar derivates was systematically studied in terms of diastereoselectivity, revealing a high degree of substrate control toward *anti*-addition. Second, we demonstrated the use of a “double Mitsunobu” reaction, inverting the stereochemistry of terminal diols. Third, the IMA toolbox was expanded to accomplish the synthesis of derivatives with up to 10 carbon atoms from particularly unreactive aldoses. Thermal investigations of all synthesized sugar alcohols, including examples with exclusive 1,3-*anti*- and suboptimal 1,3-*syn*-relationships as well as even and odd numbers of carbon atoms, were performed. We observed clear trends in melting points and thermal storage densities and discovered limitations of organic substances in this class with melting points above 240 °C as PCMs in terms of thermal stability. With our study, we provide insights into the dependence of thermal properties on structural features, thus contributing to further understanding of organic PCMs for thermal energy storage applications.

## Introduction

**Figure 1:**
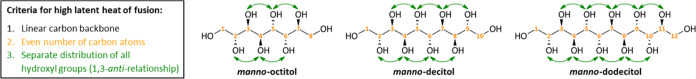
*Manno*-series of sugar alcohols designed by Inagaki and Ishida^18^ and investigated in the molecular dynamics (MD) simulations.

In nature, carbohydrates or sugars are abundant polyhydroxylated compounds that play an essential role in various biological processes. However, sugars and derivatives are not only valuable in nature but also find applications in medicinal or synthetic chemistry.^1−4^ Here, sugar alcohols have gained interest since they can act as sugar mimics and chiral synthons and have become especially popular in the design of drugs.^5−7^ Later, sugar alcohols became interesting in materials science as well, e.g., in the development of functional materials.^8,9^ Focusing on the latter, natural sugar alcohols such as, e.g., erythritol, d-mannitol, or mixtures of natural sugar alcohols have been investigated as potential phase change materials (PCMs) for thermal energy storage (TES) applications since they display thermal storage densities of up to 350 J/g.^10−16^ These values are remarkable since the already commercially exploited paraffins typically reach storage densities of 150–250 J/g.^17^ Additionally, sugar alcohols allow us to operate in a different temperature range due to their significantly higher melting points as compared to paraffins. Hence, efforts were undertaken to make sugar alcohols practically applicable to TES systems, addressing issues such as cycle stability during repeated melting and crystallization. We became interested in this compound class by a computational study by Inagaki and Ishida,^18^ where extraordinarily high thermal storage densities of up to 450–500 J/g have been predicted for higher-carbon sugar alcohols with specific structural features. These sugar alcohols were designed by the formal elongation of the natural d-mannitol following three structural criteria: a linear carbon backbone, an even number of carbon atoms, and *1,3-anti*-configuration of all hydroxyl groups (Figure 1). Such complex (to synthesize) structures will clearly not represent the next generation of energy storage materials.

**Figure 2:**
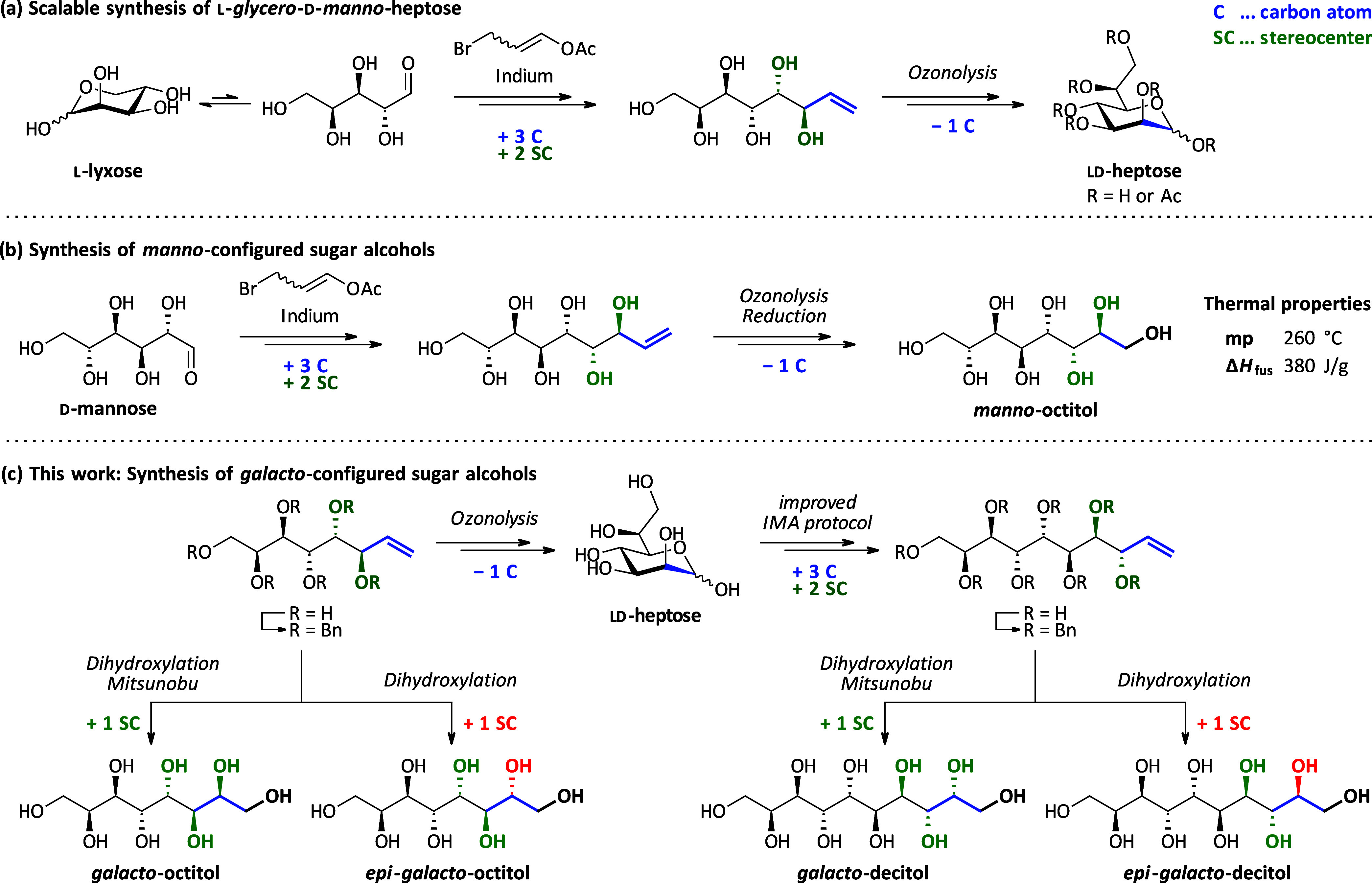
Synthesis of higher-carbon sugar species via indium-mediated acyloxyallylation—an overview.

However, we took on the challenge to synthesize higher-carbon sugar alcohols following the stated criteria to, first, compare the calculated values to experimental ones and second, confirm the importance of the structural features for high thermal storage densities since most of the calculated values could not be supported by experimental data due to the elaborate synthesis^19−23^ or inaccessibility of the compounds at that time. The addition of carbon atoms to the anomeric center of reducing sugars is often plagued by poor stereoselectivity, and often only little control is possible. Additionally, most transformations require protection strategies of the sugar species, making the transformations laborious.^24^ With the indium-mediated acyloxyallylation (IMA), a method was developed and investigated in recent years by us and others,^25−27^ which allows the direct elongation of unprotected aldoses by three carbon atoms with good control of diastereoselectivity and therefore allows a more straightforward synthesis of higher-carbon species. In the IMA, a bromopropenyl ester (acetate, benzoate, or pivaloate) is added to the aldehyde functionality of an unprotected sugar. In this transformation, the formation of four different isomers is possible since two new stereocenters are formed. However, it was found that in the case of unprotected aldoses, the isomer with *lyxo-*configuration is formed with good selectivity (>60:40 *dr lyxo*:other isomers). *Lyxo*-configuration corresponds to a *syn*-orientation of the former α-hydroxyl group and the new hydroxyl group formed from the aldehyde, and an *anti*-orientation of the two new stereocenters. The major factors that influence the success of this transformations are the solubility of the aldose, the reactivity or rather availability of the specific aldose as an aldehyde reflected by its open-chain content (OCC), and the lifetime of the indium organyl under the reaction conditions. Especially, the balance between the latter two was found to be crucial to obtain a clean transformation of the sugar to the enitol species. The IMA was shown to be a useful tool in the synthesis of the bacterial sugar l-*glycero*-d-*manno*-heptose, short ld-heptose, allowing for a short sequence at scale (Figure 2a).^26^ Furthermore, the IMA was applied by us for the synthesis of higher-carbon sugar alcohols of the *manno*-series that has been proposed and investigated in the mentioned computational study (Figure 2b).^28^ Investigations of the thermal properties of the synthesized sugar alcohols showed that the calculated thermal storage densities were in consistence with the experimentally observed ones and that sugar alcohols that fulfill the stated criteria indeed possess exceptionally high thermal storage densities, indicated as the latent heat of fusion (Δ*H*_fus_), among thermally stable, organic materials.

**Figure 3:**
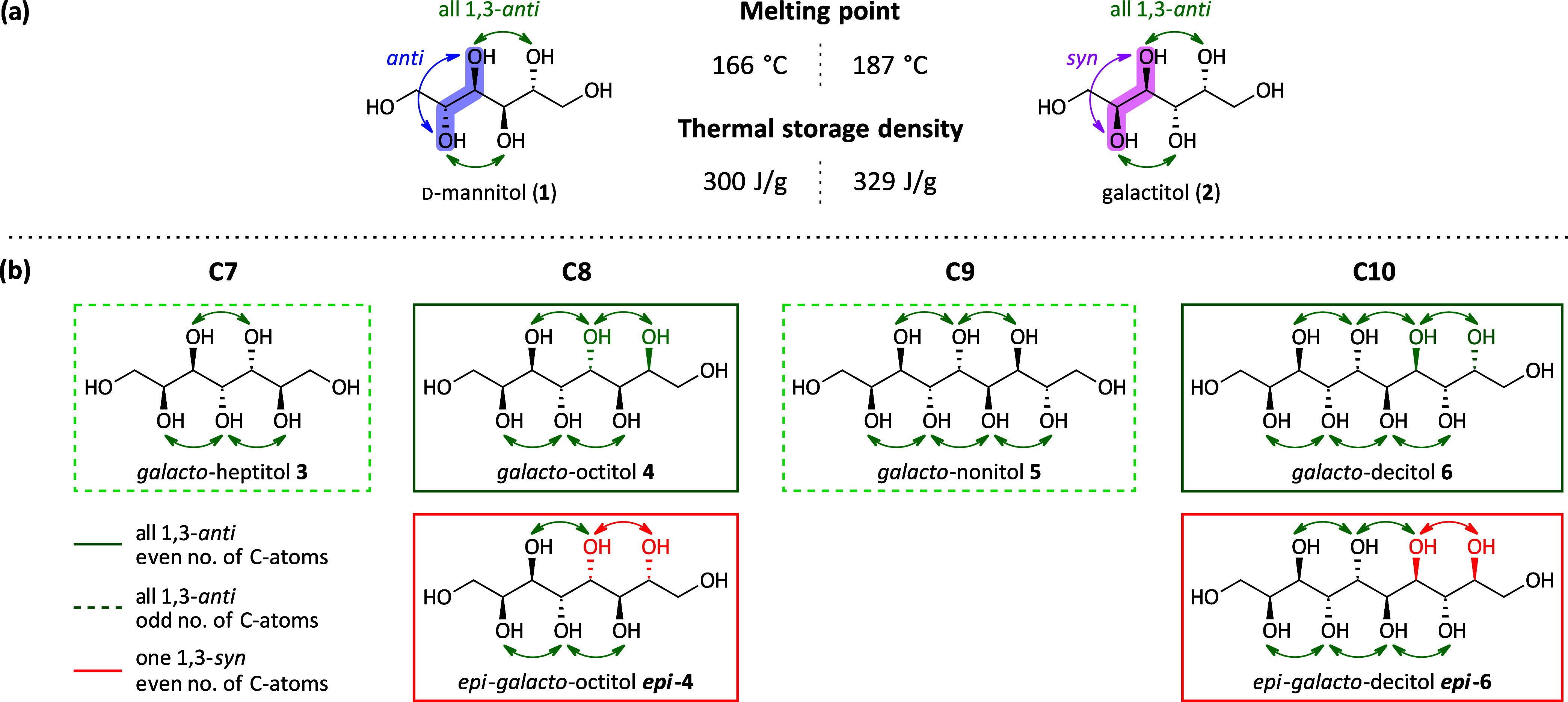
(a) d-Mannitol (**1**) vs galactitol (**2**) regarding their hydroxyl group distribution and thermal properties (determined via STA; see the Supporting Information (SI)). (b) Design of the *galacto*-series of sugar alcohols starting from galactitol, including examples not matching all stated criteria.

Within the current work, our focus was on the utilization of the IMA for the synthesis of higher-carbon sugar alcohols of the galactitol family, the only second series of sugar alcohols that fulfills the three stated criteria in order to obtain high thermal storage densities, in theory. Sugar alcohols of this so-called *galacto*-series have not been investigated in regard to their thermal properties so far, neither computationally nor experimentally. Therefore, we developed a novel strategy toward this class of sugar alcohols with up to 10 carbon atoms (Figure 2c). In the *galacto*-series, all hydroxyl groups equally are in a 1,3-*anti*-relationship, and in contrast to the *manno*-series, the two hydroxyl groups at the terminal stereocenters are in a *syn*-relationship. Noteworthily, the parent galactitol possesses an even higher thermal storage density than d-mannitol^10^ (Figure 3a). With our research, we contribute to a better understanding of the relationship between structural features of organic molecules and their thermal properties and targeted examples matching all criteria as well as examples not matching all stated criteria (Figure 3b). For consistency, throughout the manuscript, we refer to the longer homologues of the parent galactitol-stereochemistry with the desired all 1,3-*anti*-relative stereochemistry as, e.g., *galacto*-octitol, addressing the total chain length. Compounds differing from this stereochemical pattern on one of the additional stereocenters are referred to as, e.g., *epi*-*galacto*-octitol.

## Results and Discussion

### Development of the (Retro)synthetic Strategy toward *Galacto*-Configured Higher Sugar Alcohols

**Figure 4:**
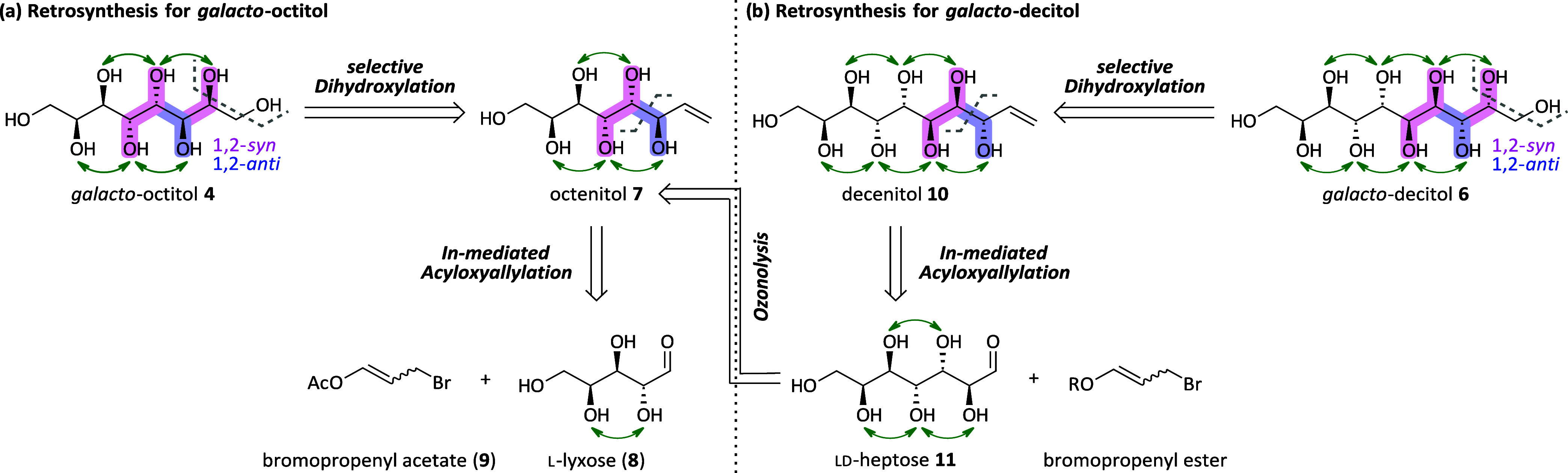
Retrosynthetic analysis for the targeted *galacto*-octitol **4** and *galacto*-decitol **6**.

In general, the retrosynthetic analysis of *galacto*-configured sugar alcohols with an even number of carbon atoms reveals two key steps in the forward synthesis: the selective dihydroxylation of the enitol species toward the sugar alcohol and the IMA of the three-carbon shorter aldose toward this enitol. These two steps can be repetitively performed starting from l-lyxose (**8**) to obtain the *galacto*-octitol **4** (C8) and *galacto*-decitol **6** (C10) (Figure 4); the latter could analogously be extended to the *galacto*-dodecitol **SI-3** (C12), in theory. Initially, focusing on the *galacto*-octitol **4** (Figure 4a), the synthesis was developed starting from the pentose l-lyxose (**8**). The sugar alcohol *galacto*-octitol **4** should be obtained from octenitol **7** via dihydroxylation (DH) with selectivity for the desired configuration of the introduced secondary hydroxyl group. Octenitol **7** with the desired stereochemical composition (all 1,3-*anti*-relationship) is accessible via indium-mediated acyloxyallylation from the reducing sugar l-lyxose (**8**) with 3-bromopropenyl acetate (**9**). This reaction is known in the literature^25,26^ and has also been demonstrated on large scale, facilitated by its superior crystallinity allowing for purification via recrystallization with a high recovery of the targeted main diastereomer. At the dihydroxylation step, we aimed for high selectivity for the *syn*-2,3-diol to obtain the sugar alcohol with all hydroxyl groups in *1,3-anti*-relationship directly from the enitol species **7**. Considering classical DH conditions using an osmium-catalyst, we assumed that especially Sharpless asymmetric dihydroxylation (AD) allows us to tune the stereochemical outcome of the reaction. The same principle retrosynthetic steps can be repeated, dissecting the longer-chain decitol **6** to the reported ld-heptose **11**, which is the oxidation product of the central octenitol **7** (Figure 4b).

#### Synthesis of Octitols via IMA and Dihydroxylation

**Scheme 1:**
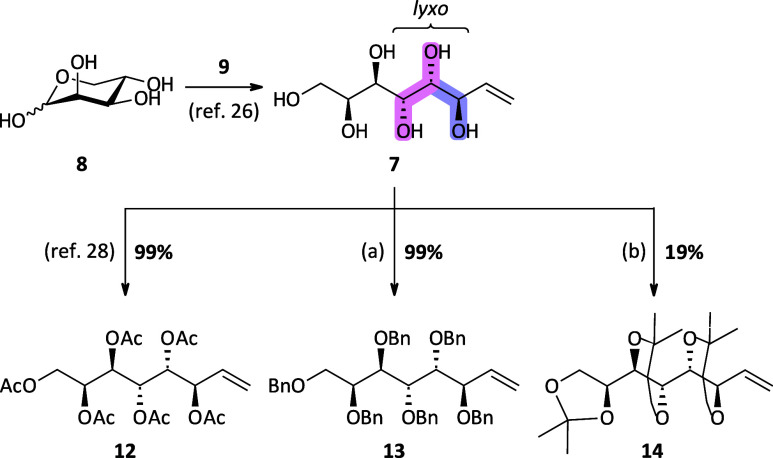
Synthesis of Differently Protected Enitol Species **12**–**14** (a) BnBr, NaH, *n*-Bu_4_NI, *N*,*N*-dimethylformamide (DMF), rt, 18 h; (b) 2,2-dimethoxypropane, *p*-TSA·H_2_O, DMF, 50 °C, 48 h.

For the planned dihydroxylation reaction, protection of the reported octenitol **7**(26) was necessary to achieve solubility of the substrate in an organic solvent. Three different protecting groups (PGs) were chosen to investigate their influence on the stereochemical outcome of the DH. Hexaacetyl **12** and hexabenzyl octenitol **13** were obtained in excellent yields using standard reaction conditions. Furthermore, three acetonide protecting groups could be introduced to the six hydroxyl groups present in the octenitol using 2,2-dimethoxypropane, giving compound **14**, although with a significantly lower yield of only 19%, mainly due to the formation of an isomeric triacetonide, complicating purification (Scheme 1). Targeted product **14** was clarified by 2D-NMR and ^13^C NMR analysis based on an NMR study from the literature.^29^ The structure of the second octenitol species with also three isopropylidene groups present could not be unambiguously clarified by NMR analysis (see the SI).

With the three protected octenitols **12**–**14**, different dihydroxylation (DH) conditions were tested to investigate the dependency of the stereochemical outcome on the used protecting group and DH conditions (Table 1). Two different protocols were considered: (1) Upjohn dihydroxylation using K_2_[OsO_2_(OH)_4_] as the catalyst and stoichiometric amount of *N*-methylmorpholine-*N*-oxide (NMO) as a co-oxidant and (2) Sharpless asymmetric dihydroxylation (AD) using the two different mixtures of reagents, namely, AD-mix-α and AD-mix-β, to induce facial selectivity. Early attempts showed that the use of a solvent mixture *t*-BuOH/H_2_O/dichloromethane (DCM) in a ratio of 1:1:1 resulted in faster reaction progress due to the substrate’s better solubility in the reaction mixture compared to the standard solvent system *t*-BuOH/H_2_O. In the case of Sharpless AD conditions, the use of commercially available AD-mix-α/β resulted in sluggish reaction progress. However, with a three times concentrated AD-mix (for preparation, see the SI) with higher loadings of osmate and ligand, a modification adopted from Kobayashi et al.,^30^ reaction rates could be accelerated significantly. The catalyst-enriched AD-mixes are stated as AD-mix-α (×3) and AD-mix-β (×3).

**Table 1:**
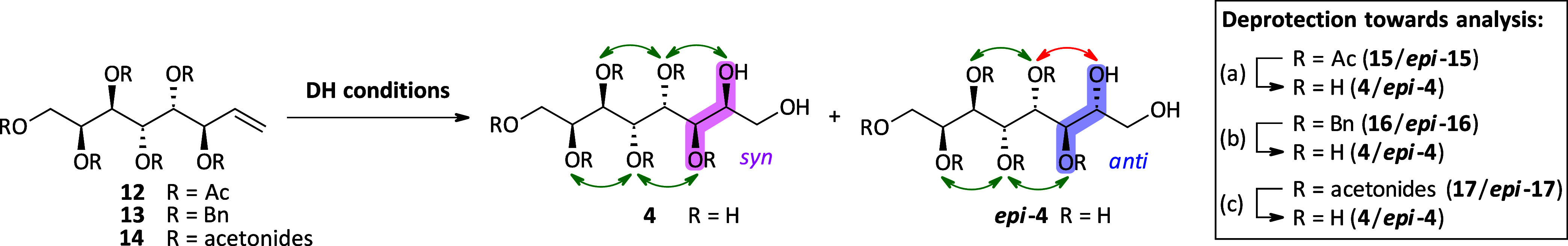

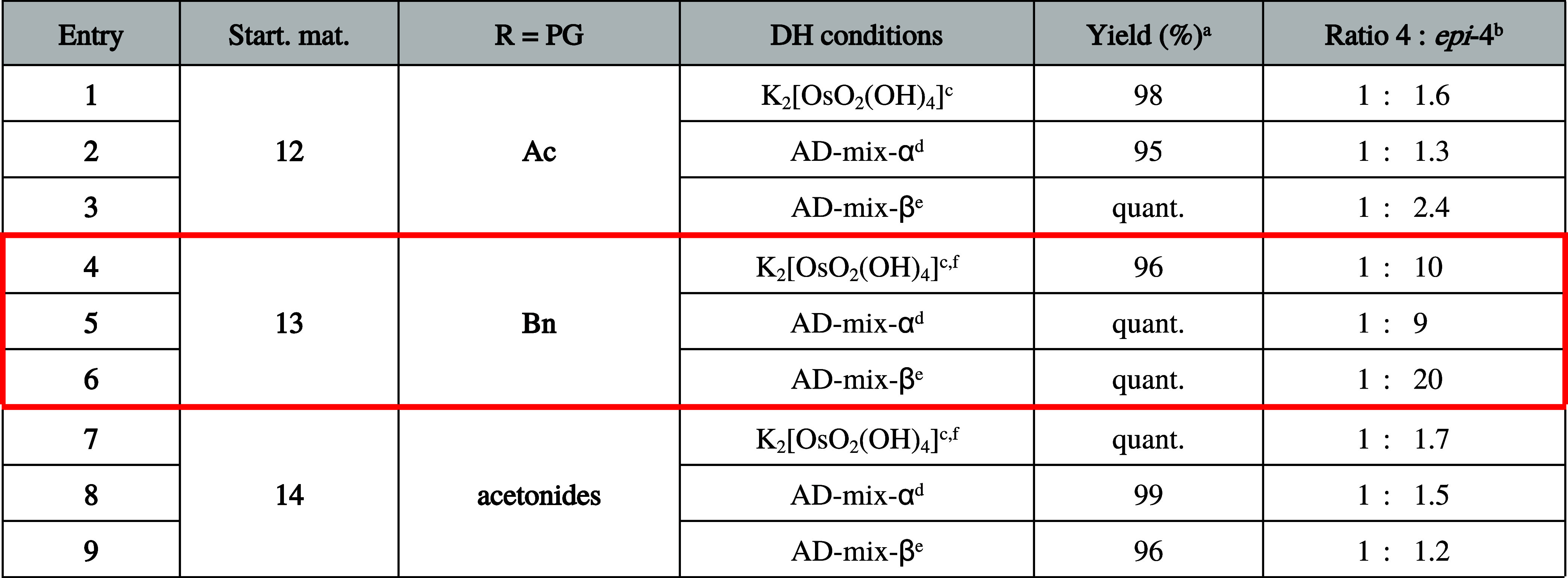
Screening Results for Different Dihydroxylation (DH) Conditions and Octenitol Substrates **12**–**14** on Analytical Scale

a Isolated yield over 2 steps.

b Product ratio determined upon deprotection via integration from ^13^C NMR (see Figure 5).

c Upjohn conditions: K_2_[OsO_2_(OH)_4_] (1 mol %), NMO·H_2_O (1 equiv), *t*-BuOH/H_2_O (1:1, 0.1–0.3 M), rt.

d Sharpless conditions A: AD-mix-α (×3) (1.4 g/mmol substrate), MsNH_2_ (2 equiv), *t*-BuOH/H_2_O/DCM (1:1:1, 0.3 M), rt.

e Sharpless conditions B: AD-mix-β (×3) (1.4 g/mmol substrate), MsNH_2_ (2 equiv), *t*-BuOH/H_2_O/DCM (1:1:1, 0.3 M), rt.

f *t*-BuOH/H_2_O/DCM (1:1:1) solvent mixture used; **deprotection toward analysis:** (a) NaOMe (30% in MeOH), MeOH, rt, 1 h; (b) H_2_ (1 atm), Pd/C (10 wt %), MeOH, rt, 18 h; and (c) Dowex-H^+^, H_2_O, 80 °C, 2 h.

The different pairs of protected diastereomeric octitols were all deprotected toward **4/*****epi*****-4** to facilitate comparative analysis of diastereomeric ratios at that stage via NMR analysis. Due to overlapping signals, the quantification could not be deduced from ^1^H NMR, but the two diastereomers could easily be distinguished in ^13^C NMR spectra (see Figure 5). For a mixture of both sugar alcohols, four signals from the targeted symmetrical 2,3-*syn* product **4**, each representing two carbon atoms, and eight signals from the asymmetrical 2,3-*anti* product ***epi*****-4** were observed in the ^13^C NMR spectra. Based on a detailed study by Otte et al.,^31^ sufficiently similar response factors for the ^13^C NMR spectroscopic integration were assumed for diastereomers even with standard ^13^C NMR techniques (standard pulse sequences with broad-band decoupling and short D1 values) following two rules: (1) the two different compounds must possess the same number of hydrogen atoms and (2) the ratios of integrals need to be averaged over all different carbon atoms. This technique was used for the estimation of the diastereomeric ratio **4**: ***epi*****-4**. In Table 1, the screening results for all protected octenitol substrates **12**–**14** and DH conditions are summarized. Detailed reaction conditions and procedures for all dihydroxylations and deprotection steps can be found in the SI.

**Figure 5:**
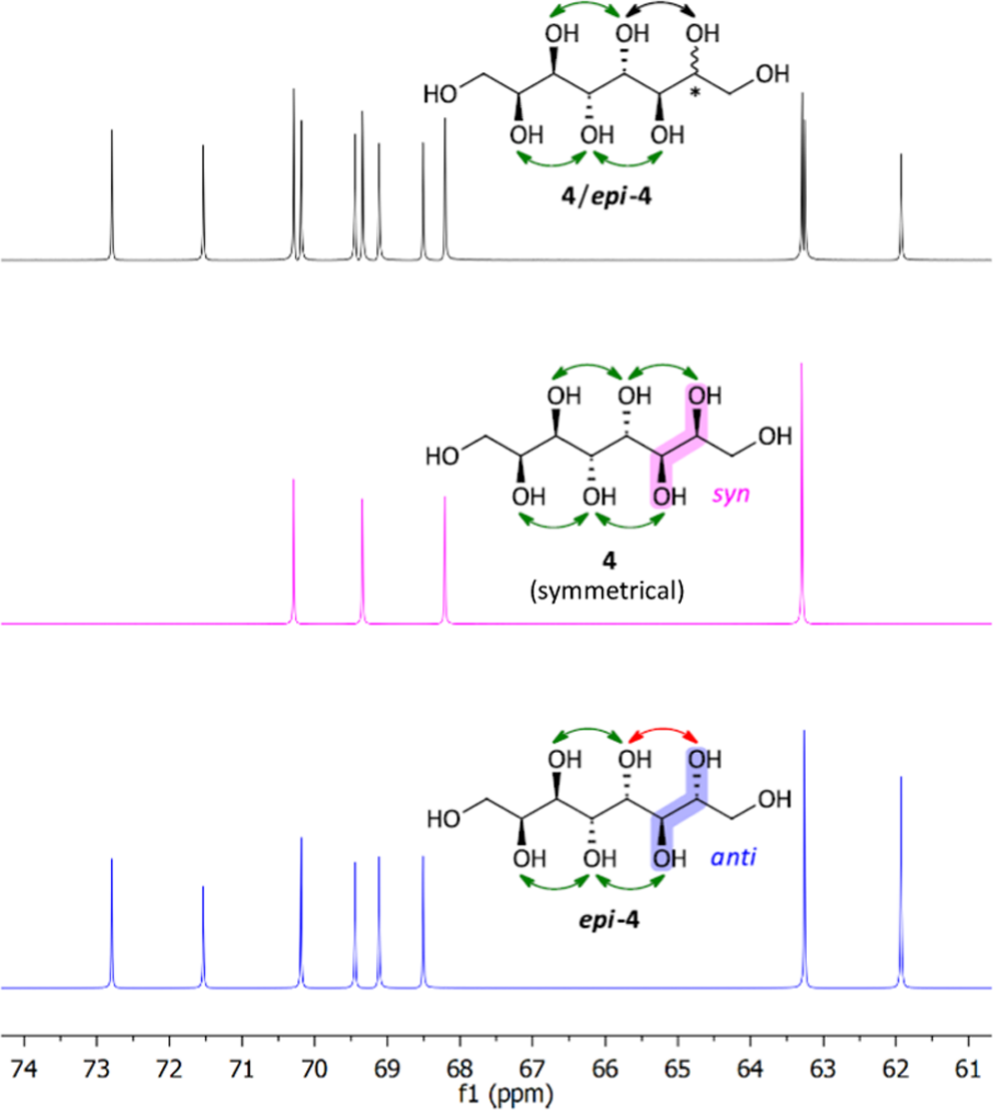
Comparison of the ^13^C NMR-spectra of a roughly 1:1.6 mixture of **4** and ***epi*****-4** (top, black), pure symmetric 2,3-*syn* product **4** (middle, magenta), and pure 2,3-*anti* product ***epi*****-4** (bottom, blue).

In all cases, the product with an *anti*-relationship between the introduced secondary hydroxyl group and the existing alkyl-/acyloxy group was preferably formed independent of the substrate and the DH conditions. Surprisingly, both AD-mixes showed selectivity toward the 2,3-*anti* product in all cases. Acetate and acetonide protection (Table 1, entries 1–3 and 7–9) delivered only moderate selectivity; however, outstandingly high selectivity for the *anti* product was obtained for substrate **13** with benzyl protection under the used DH conditions (Table 1, entries 4–6).

### Preparative Synthetic Route toward *Galacto*-octitol and *En Route* Synthesis of the C2 Epimer *Epi*-*galacto*-octitol

Since none of the investigated substrates **12**–**14** preferably formed the 2,3-*syn* product under any of the applied dihydroxylation conditions, an adaption of the planned synthetic route to obtain *galacto*-octitol **4** with all hydroxyl groups in a 1,3-*anti*-relationship was necessary. The idea was to perform the dihydroxylation with high selectivity for the 2,3-*anti* product and then invert the stereochemistry of the secondary hydroxyl group via a Mitsunobu reaction. Since in our case two free hydroxyl groups are present in the molecule, we planned for a “double Mitsunobu” reaction where both hydroxyl groups are esterified using an excess of reagents. Examples from the literature^32,33^ with sterically even more demanding substrates showed that the “double Mitsunobu” of 1,2-diols is in general feasible. Therefore, the DH was carried out with the hexabenzyl octenitol **13** under Sharpless AD conditions using AD-mix-β (×3) since this set of protecting group and conditions gave the highest selectivity with ∼20:1 *dr* (***epi*****-16**/**16**) (Table 1, entry 6). However, the conditions had to be adapted to the larger scale in terms of higher catalyst loading to increase the reaction rate, subsequently obtaining the product mixture ***epi*****-16**/**16** in excellent yield. At this stage of the sequence, no efficient preparative separation was achieved and the mixture of diastereomers was submitted to the next step, the Mitsunobu reaction. The reaction was carried out with PPh_3_, diisopropyl azodicarboxylate (DIAD), and 4-nitrobenzoic acid, using 5 equiv each relative to the substrate. Expecting superior crystallinity of the targeted *galacto*-octitol **4**, initially, the diastereomeric mixture **18**/***epi*****-18** with the 2,3-*syn* product **18** now as the major component was directly deprotected via transesterification using NaOMe in MeOH followed by reductive hydrogenation using Pd on activated charcoal in MeOH. As expected, a 20:1 mixture (calculated from ^13^C NMR, *vide supra*) of the *galacto*-octitol **4** with all hydroxyl groups in 1,3-*anti*-relationship and the *epi*-*galacto*-octitol ***epi*****-4** with one 1,3-*syn*-relationship was obtained. However, in contrast to the enitol species, where the perfectly configured diastereomer (*lyxo*-configuration) **7** could be effectively isolated via recrystallization, the minor octitol ***epi*****-4** could not be removed completely by recrystallization of the obtained mixture **4**/***epi*****-4** from MeOH/H_2_O mixtures or pure H_2_O.

**Scheme 2:**
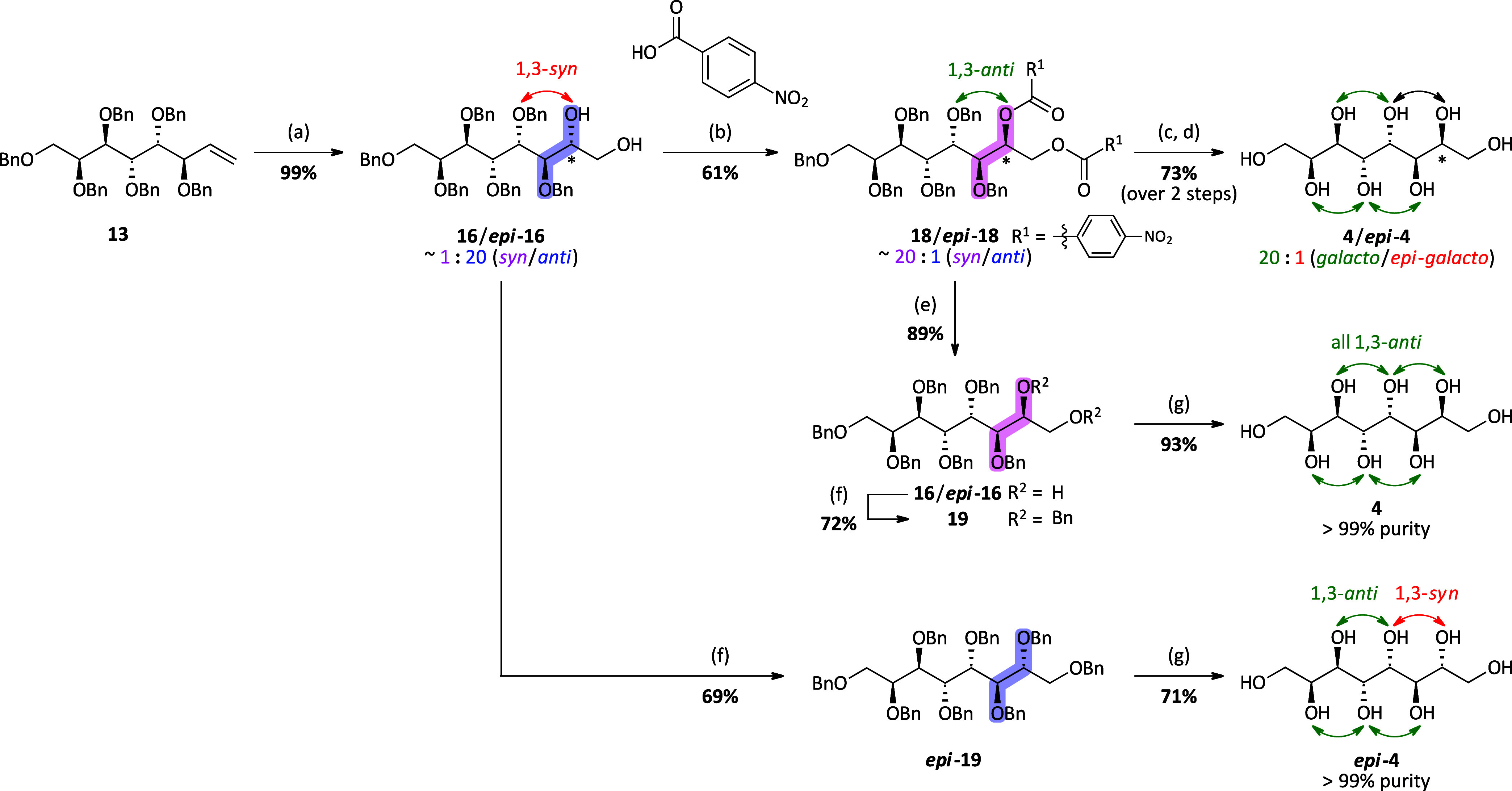
Synthetic Route toward the *Galacto*-octitol **4** and *Epi*-*galacto*-octitol ***epi*****-4** (a) AD-mix-β (×3), MsNH_2_, *t*-BuOH/H_2_O/DCM (1:1:1), rt, 12 h; (b) PPh_3_, DIAD, tetrahydrofuran (THF), 0 °C, then ***epi*****-16**/**16** and 4-nitrobenzoic acid, 0–45 °C, 24 h; (c) NaOMe (30% in MeOH), MeOH, rt, 2 h; (d) H_2_ (1 atm), Pd/C (10 wt %), HCl (cat.), MeOH, rt, 14 h; (e) aq. LiOH (1 M), THF, rt, 1.5 h; (f) BnBr, NaH, *n*-Bu_4_NI, DMF, rt, 18 h; (g) H_2_ (1 atm), Pd/C (10 wt %), AcOH (cat.), MeOH/EtOAc (1:1), 50 °C, 18 h.

Alternatively, it was found that the transformation into the corresponding octabenzyl species allows separation of the minor diastereomer ***epi*****-19** by standard flash column chromatography using a slow gradient. Therefore, the free hydroxyl groups in **16** obtained after cleavage of the ester groups were protected with benzyl groups and diastereomer **19** was obtained in pure form (>99%) after column chromatography. *Galacto*-octitol **4** was finally isolated after catalytic hydrogenation, cleaving all present benzyl groups. In contrast to the conditions used earlier (Scheme 2, conditions (d)), the solvent was switched to a MeOH/EtOAc mixture (1:1) since compound **19** was insoluble in pure MeOH. Additionally, the reaction was performed at 50 °C instead of rt for better solubility of the formed intermediates, allowing the cleavage of all benzyl groups at atmospheric pressure within 18 h, giving the *galacto*-octitol **4**. Noteworthily, both perbenzylated **19** and octitol **4** are symmetric compounds, thus proving the stereochemistry to be the annotated one. *En route*, the C2 epimer ***epi*****-4** was synthesized directly from the initial dihydroxylation product ***epi*****-16** following the same protocol as it has been used targeting pure *galacto*-octitol **4**, namely, benzylation and furnishing pure ***epi*****-19** via chromatographic separation followed by deprotection via catalytic hydrogenation. The *epi*-*galacto*-octitol ***epi*****-4** was not the main target within this synthesis route but is of interest when it comes to thermal properties since the distribution of the hydroxyl groups is expected to have a high influence on the melting point and melting enthalpy.

### Synthesis of Decitols and Nonitol via Elongation of l-*Glycero*-d-*manno*-Heptose by IMA

Analogous to the synthetic route toward the octitols, the synthesis of the decitols was started from l-*glycero*-d-*manno*-heptose (**11**) that is accessible via ozonolysis of octenitol **7** as described in the literature.^26^ Initial attempts for the elongation of heptose **11** toward the decenitol using the standard IMA conditions showed only little conversion to the enitol species. Therefore, we had a deeper look into the IMA of heptose **11**, a less reactive aldose, and successfully developed a protocol to achieve clean conversion.

#### An Improved Protocol for the IMA Allowing the Elongation of Less Reactive Aldoses

**Scheme 3:**
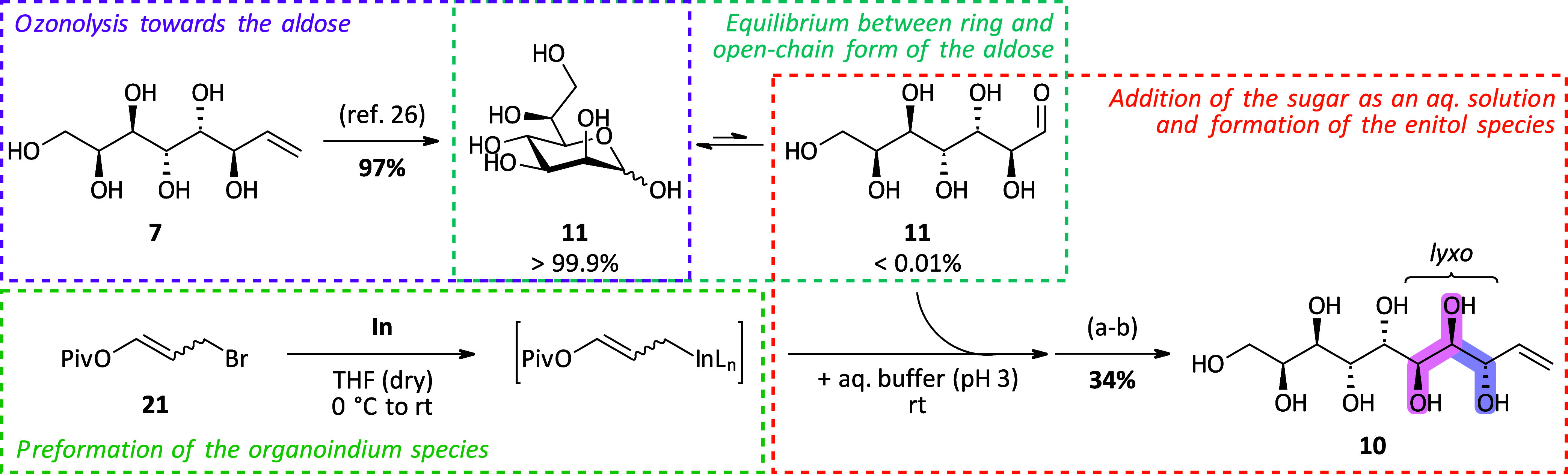
Elongation of Heptose **11** under Grignard-Type Conditions toward Decenitol **10** (a) Ac_2_O, 4-dimethylaminopyridine (DMAP), pyridine, rt, 18 h; (b) NaOMe (30% in MeOH), MeOH, rt, 3 h; trituration (MeOH); recrystallization (H_2_O).

In general, fast and complete consumption of the sugar is of high importance when performing IMA. Otherwise, side reactions such as the formation of ethyl glycosides in an acid-catalyzed Fischer glycosylation^25^ and Wurtz-type dimerization^34^ of the organoindium species upon hydrolysis take over. Furthermore, the purification becomes elaborate since purification via column chromatography must be performed at the peracetylated stage in such cases. Generally, the success of the outcome of this protocol mainly depends on two properties of the used sugar: its open-chain content (OCC), representing the availability of the free aldehyde moiety in solution, and its solubility in the reaction mixture. In some cases, these issues can be overcome by decreasing the concentration of the sugar (<1%) and increasing the reagent amount, which has been shown earlier in our group in the case of d-mannose.^28^ However, with this protocol, the reaction progress is sluggish and fast addition of indium and efficient stirring become even more crucial. In Table 2, the investigations on the IMA for heptose **11** are summarized. The standard Barbier-type protocol did not proceed with full consumption of the starting material even when more equivalents of reagent were used (4 equiv In, 6 equiv 3-bromopropenyl acetate (**9**)) (Table 2, entry 1). Since heptose **11** is even less soluble in EtOH than d-mannose and was found to exhibit a similarly low open-chain content (∼0.03%, see the SI), first, the solvent was changed to dioxane/H_2_O (8:1) and the reaction was performed with benzoate ester **20** (Table 2, entry 2), following the strategy from Palmelund and Madsen.^25^ However, this also resulted in low conversion of heptose **11** to the enitols (∼20% based on quant. ^1^H NMR with maleic acid as the internal standard) even when more robust pivalate ester **21**(27) was used as the elongation reagent (Table 2, entry 3). Next, a two-step Grignard-type protocol was tested (Table 2, entries 4 and 5). This synthetic protocol, developed by Lombardo et al.^34^ for simple aldehydes, includes the preformation of the organoindium species in anhydrous THF and addition of the aldehyde species as a solution in acid phthalate buffer (pH 3) (for the detailed protocol, see Scheme 3). Using the benzoate ester **20** (Table 2, entry 4), still no complete consumption of the sugar was observed (∼40% based on quant. ^1^H NMR with maleic acid as the internal standard). Only when we combined this approach with the more stable pivalate reagent **21** (Table 2, entry 5), full consumption of heptose **11** was achieved and ^1^H NMR analysis after acetylation and global deprotection showed that the desired *lyxo*-isomer **10** was again the major isomer formed (70:30 *dr lyxo*:other isomers), with even slightly increased selectivity over the standard Barbier-type protocol. To the best of our knowledge, this Grignard-type protocol has never been implemented in the IMA of reducing sugars and the necessity for the pivalate reagent **21** indicated the importance of matching kinetics of the mutarotation of the equilibrating sugar and the decomposition of the reagent. This protocol, in our experience, represents the current last resort when targeting a particularly unreactive (masked) aldehyde species.

**Table 2:**
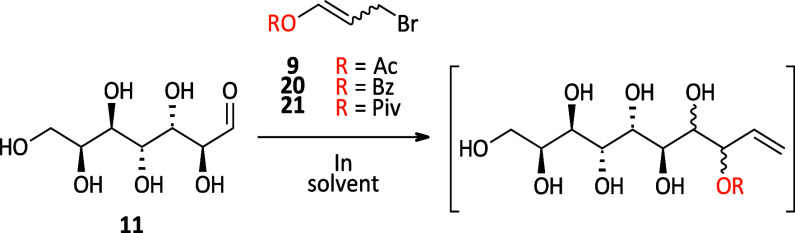

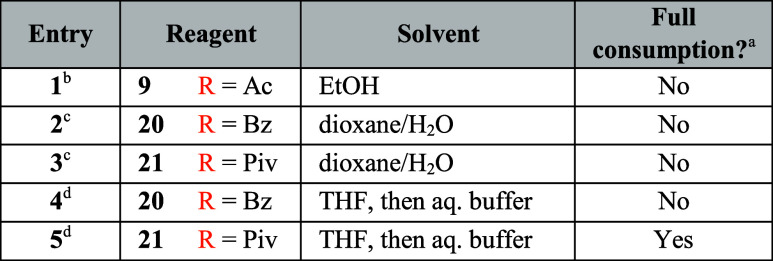
Redevelopment of the IMA for the Less Reactive Aldose l-*Glycero*-d-*manno*-heptose (**11**)

a Reaction monitoring via TLC (CHCl_3_/MeOH/H_2_O 14:7:1)—product was not isolated.

b Barbier-type protocol: **11** (1 equiv) in dry EtOH (0.1 M), 48 °C, subsequent addition of In (3 equiv) and **9** (4.5 equiv).

c Barbier-type protocol: **11** (1 equiv) in dioxane/H_2_O (8:1, 0.1 M), 60 °C, subsequent addition of In (2 equiv) and **20** (entry 2) or **21** (entry 3) (3 equiv).

d Grignard-type protocol: step 1—In (2 equiv) in dry THF (0.8 M), addition of **20** (entry 4) or **21** (entry 5) at 0 °C, then rt, 45 min; step 2—addition of **11** (1 equiv) in acid phthalate buffer (pH 3) (2.7 M), rt, 45 min.

With this redeveloped protocol in hand, the elongation of heptose **11** was carried out on a larger scale (16 mmol) using the two-step Grignard-type protocol (Scheme 3). In the first step, the organoindium species was preformed by the addition of 3-bromopropenyl pivalate (**21**) to a suspension of indium in anhydrous THF under an argon atmosphere at 0 °C. This was then vigorously stirred at rt for about 40 min until no residual indium powder could be observed. In the second step, sugar **11** was dissolved in acid phthalate buffer (pH 3) (for details, see the Experimental Section) and added to the reagent solution at once. The mixture of decenitol diastereomers with the *lyxo*-isomer **10** as the major component (*lyxo*/*xylo*/*ribo* isomers in ratio 70:18:12, determined via integration of H-3 in ^1^H NMR; see the SI) was then isolated upon peracetylation, aqueous workup, and subsequent deprotection. The stereochemical configuration of the diastereomers formed in the IMA is proposed based on the findings of prior research^26−28^ that revealed consistent stereochemical patterns across a range of carbohydrates, with respect to the stereochemistry at newly formed centers C-2 and C-3. The *lyxo*-decenitol **10** could again be obtained in pure form via trituration with MeOH and final recrystallization from H_2_O due to its superior crystallinity compared to the other diastereomers with an overall yield of 34% over 3 steps. Noteworthily, when our new protocol was applied in the attempted elongation of C9-homologue l-*lyxo*-l-*manno*-nonose (**SI-1**) (with equal OCC compared to heptose **11**) even with increased amounts of reagents, the *lyxo*-dodecenitol product **SI-3** could only be isolated in a total yield of 11% (see the SI). According to our interpretation, the increasing number of additional hydroxyl groups caused an additional burden for the successful IMA in addition to solubility and open-chain content.

#### The Synthesis of *Galacto*-decitol, *Epi*-*galacto*-decitol, and *Galacto*-nonitol

**Scheme 4:**
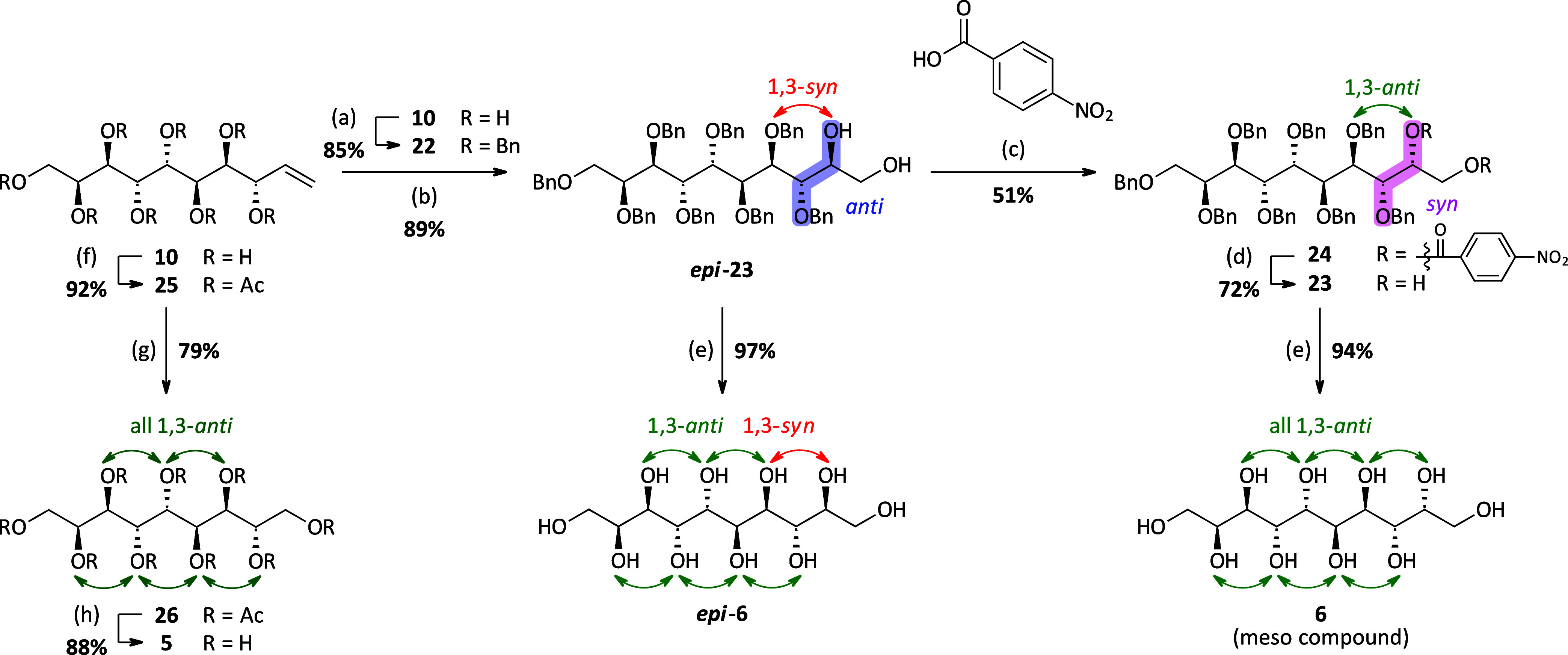
Synthetic Route toward Decitols **6** and ***epi*****-6** and Nonitol **5** Starting from Decenitol **10** (a) BnBr, NaH, *n*-Bu_4_NI, DMF, rt, 18 h; (b) AD-mix-β (×3), MsNH_2_, *t*-BuOH/H_2_O/DCM (1:1:1), 2 d; (c) PPh_3_, DIAD, THF, 0 °C, then ***epi*****-23** and 4-nitrobenzoic acid, 0–45 °C, 18 h; (d) aq. LiOH (0.5 M), THF, 0 °C to rt, 18 h; (e) H_2_ (50 bar), Pd/C (10 wt %), AcOH (cat.), EtOAc/MeOH (4:1), rt, 4–7 d; (f) Ac_2_O, DMAP, pyridine, rt, 2 h; (g) (i) O_3_, DCM/MeOH (3:1), −78 °C, 30 min, then NaBH_4_, rt, 12 h, (ii) Ac_2_O, DMAP, pyridine, rt, 2 h; and (h) NaOMe (30% in MeOH), MeOH, rt, 2 h.

With decenitol **10** in hand, the synthesis of *galacto*-decitol **6** and *epi*-*galacto*-decitol ***epi*****-6** was performed, repeating the developed synthetic strategy for octitols **4** and ***epi*****-4** (Scheme 4). Enitol **10** was protected with benzyl groups, giving the octabenzyl enitol **22** in good yield, and the dihydroxylation reaction was performed using AD-mix-β (3×), giving again the *anti*-2,3-diol product ***epi*****-23** with high selectivity (>10:1 *dr*). However, in the case of the C10-sugar species, the product ***epi*****-23** could be isolated in pure form at this stage via column chromatography using a slow gradient. Targeting the *galacto*-decitol **6**, the “double Mitsunobu” reaction was performed as the next step to invert the stereochemistry of the previously introduced secondary hydroxyl group. This step gave comparably lower yields (51%), but the inversion was equally successful as above. In the next step, the ester groups were cleaved via ester hydrolysis using LiOH in THF, giving **23**, which was finally submitted to catalytic hydrogenation to obtain the free sugar alcohol **6**. As already discussed for the octitol (*vide supra*), solubility issues played a major role in the successful cleavage of all benzyl groups. In the case of the partly protected decitol **23**, it was necessary to use EtOAc/MeOH in a 4:1 mixture as the solvent and perform the reaction at low concentrations (1%). Furthermore, high Pd loadings of ∼20 mol % were required, and the hydrogenation had to be performed in a high-pressure laboratory autoclave at 50 bar H_2_-pressure to achieve conversion to the fully deprotected sugar alcohol **6**, although with long reaction times of up to 7 days. However, with this optimized setup, the targeted sugar alcohol **6** could be obtained in excellent yield and isolated in high purity upon trituration with EtOH and H_2_O. Again, decitol **6** is a symmetric compound, more precisely a *meso* compound, giving only five signals in the ^13^C NMR spectrum for the 10 carbon atoms present, thus again proving the stereochemistry to be the annotated one. The *epi*-*galacto*-decitol ***epi*****-6** was obtained via catalytic hydrogenation from the dihydroxylation product ***epi*****-23**, using the same reaction conditions as described for the *galacto*-decitol **6**. *En route*, the *galacto*-nonitol **5** was easily accessible using the ozonolysis and reduction protocol that has already been established for the synthesis of higher sugar alcohols by Draskovits et al.,^28^ starting from the peracetylated decenitol **25**.

### Investigations on the Thermal Properties of the Synthesized Sugar Alcohols

With the sugar alcohols in hand, we moved on to investigate their thermal properties since the configuration of the *galacto*-series increased the expectation that similar high heats of fusion can be expected as calculated by Inagaki and Ishida^18^ and partly already experimentally confirmed^28^ for the *manno*-series. Among other criteria such as melting point, thermal conductivity, and thermal stability, a high heat of fusion is crucial for a high-energy storage density. For organic PCMs, high storage densities are referred to values >200 J/g. Currently, paraffins are most widely applied, even though their storage densities are at the lower end of the previously defined range, but they display excellent thermal stability.^17^ For example, paraffin PCMs with melting temperatures of 30–60 °C were found to be useful for solar water heating systems and integration with heat pumps.^35^ Organic PCMs, however, usually possess melting temperatures for applications below 180 °C, and, in general, fewer PCMs in the middle- to high-temperature range (150–250 °C) have been developed, a temperature range that was found to be covered by the synthesized sugar alcohols within this paper.

**Figure 6:**
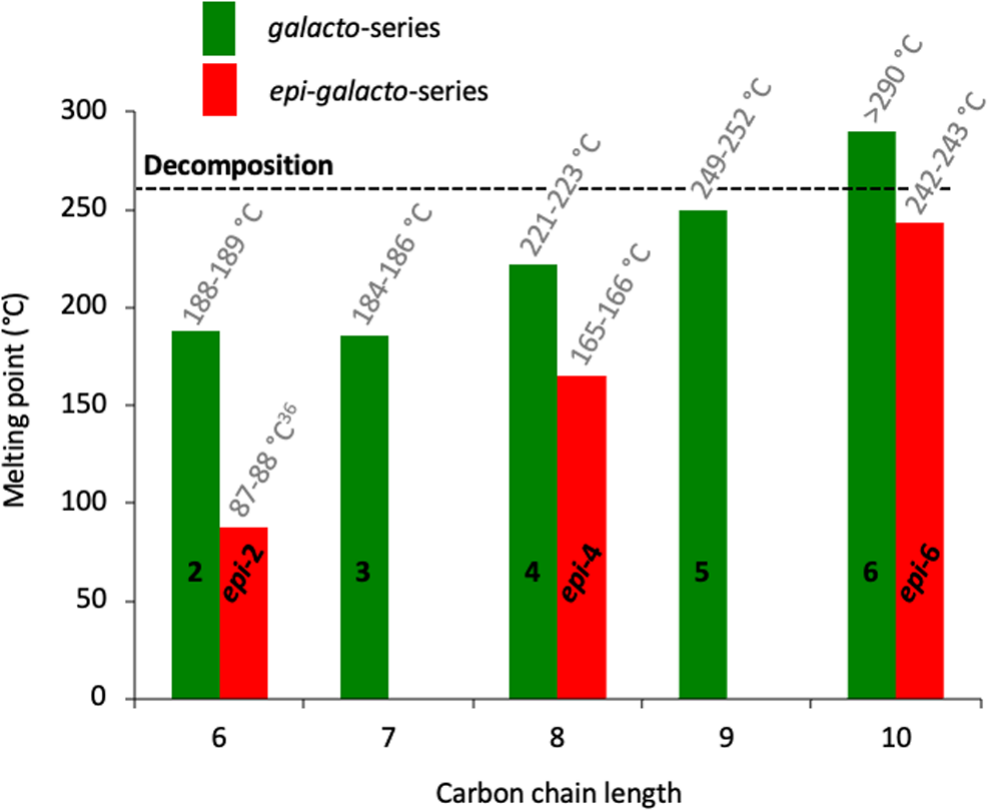
Experimental melting points (°C) determined on a Kofler-type instrument of the synthesized sugar alcohols and the C6 sugar alcohols galactitol and l-altritol (literature value^36^) in comparison. The synthesis of heptitol **3** has been reported in the literature.^28^

For further investigations, all synthesized sugar alcohols were recrystallized from H_2_O or MeOH/H_2_O mixtures since our previous work^28^ revealed a major impact of the degree/quality of crystallinity on the material’s behavior during the heating process regarding sharp melting points and high thermal storage densities. However, despite employing slow cooling rates and variations in the solvent system, we were only able to obtain micro- to nanocrystalline material for all sugar alcohols. This observed crystallization behavior has so far precluded the acquisition and description of X-ray structures within our work. We moved on to investigate their melting behavior using a Kofler-type micro-hot stage microscope. For the sugar alcohols with up to nine carbon atoms, sharp melting points could be observed, matching the data presented in the earlier literature (see the Experimental Section). However, for the *galacto*-decitol **6**, decomposition at a temperature of >290 °C was observed, indicating that the material is not stable at this high temperature anymore. For the *epi*-*galacto*-decitol ***epi*****-6**, melting was observed at 242–243 °C, but at temperatures >260 °C also, decomposition of the material was noticed, giving a dark, “burned” solid on the slide. Nevertheless, the trends of the melting points could be observed in agreement with the proposed criteria of Inagaki and Ishida^18^ for the *manno*-series (Figure 6): first, the perfectly aligned sugar alcohols with all hydroxyl groups in a 1,3-*anti*-relationship, namely, galactitol (**2**, 188–189 °C), *galacto*-octitol **4** (221–223 °C), and *galacto*-decitol **6** (>290 °C), have significantly higher melting points compared to their C2-epimers l-altritol (***epi*****-2**, 87–88 °C^36^), *epi*-*galacto*-octitol ***epi*****-4** (165–166 °C), and *epi*-*galacto*-decitol ***epi*****-6** (242–243 °C). This is expected to be the result of the superior crystallinity of the *galacto*-configured sugar alcohols due to the perfect alignment of all hydroxyl groups. Second, with the length of the carbon chain in the molecule’s backbone also, the melting point increases.

**Figure 7:**
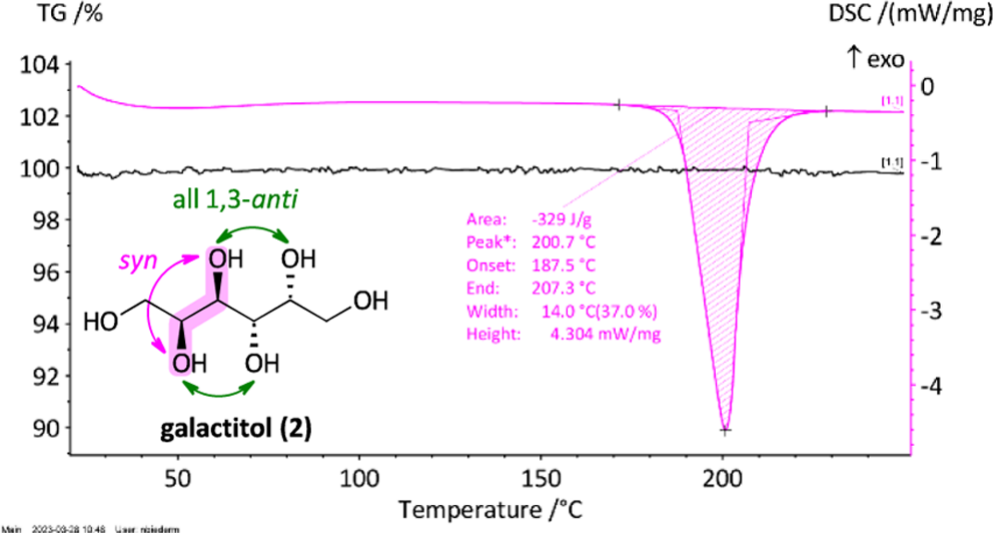
Typical STA measurement exemplified on galactitol (**2**) showing the DSC-curve (magenta) and TG-curve (black).

**Figure 8:**
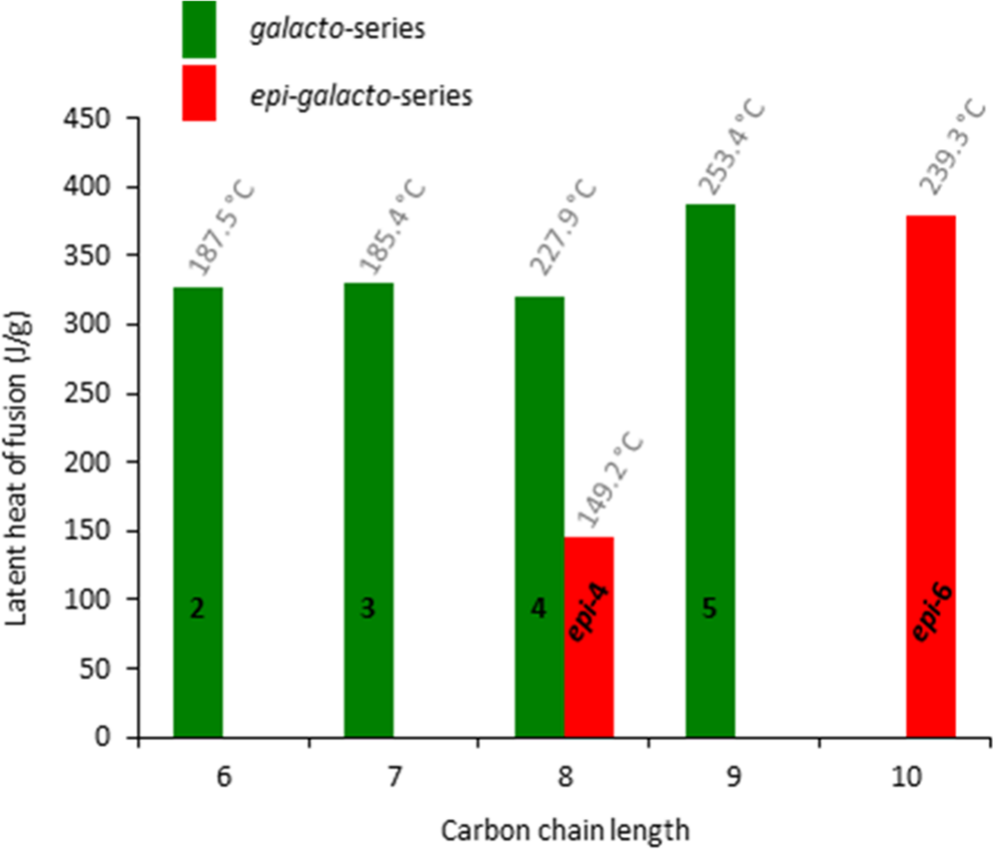
Experimental latent heats of fusion (J/g) of the sugar alcohols and their melting points obtained from the extrapolated onsets of the DSC-curves, displayed on top of the corresponding bar. In the case of the ***epi*****-4**, the measured melting point via DSC was significantly lower than the melting point measured using the Kofler-type micro-hot stage microscope.

Next, we moved on to investigate all compounds in simultaneous thermal analysis (STA) that combines differential scanning calorimetry (DSC) with thermogravimetric analysis (TG), allowing the simultaneous measurement of melting enthalpy and weight losses of the sample. In general, weight losses can be attributed either to residual solvent present in the sample or thermal instability of the material leading to decomposition. In Figure 7, a typical STA measurement is depicted where the DSC-curve (values in mW/mg, magenta) and TG-curve (in %, black) are displayed in dependency of the temperature (in °C, *x*-axis). The melting enthalpy and therefore latent heat of fusion (in J/g) can be determined from the peak area and the melting point (in °C) from the onset of the curve. Detailed information on the measurements can be found in the SI. As stated earlier, crystallinity of the material played a major role in the behavior of the compound during the heating process. Samples that were not recrystallized did not give well-shaped curves. For galactitol **2**, the obtained values were in agreement with the literature.^13^ For *galacto*-heptitol **3** (330 J/g) and *galacto*-octitol **4** (321 J/g), melting enthalpies in the same range were observed (Figure 8), the latter quite in contrast to the findings in the *manno*-family.^28^ Some thermal decomposition during the melting process was observed for the high-melting sugar alcohols *galacto*-nonitol **5** and *epi*-*galacto*-decitol ***epi*****-6**. Still, the measured DSC-curves could be processed to give an approximate value for their melting enthalpies (**5**: 387 J/g, ***epi*****-6**: 146 J/g). For *galacto*-decitol **6**, a high degree of degradation was observed, and the DSC-curve did not show a sharp peak for the melting process (see the SI, Figure S10). The measured latent heats of fusion of the sugar alcohols together with the melting points determined from the onset of the peak in the DSC-curve are displayed in Figure 8, and the corresponding curves can be found in the SI.

In contrast to the findings for *manno*-configured sugar alcohols,^18,28^ for the *galacto*-configured octitol **4**, a latent heat of fusion of only in the same range as for the “parent” sugar alcohol galactitol **2** (∼300 J/g) was observed. In line with the rules of prediction, for the *epi*-*galacto*-octitol ***epi*****-4**, a significantly lower value of ∼150 J/g, compared to the “perfect” *galacto*-octitol **4** was measured; however, this compound also showed thermal degradation during the melting process, which also exhibited a broad melting range and an early onset, which contributes to the lower value observed. Surprisingly, *galacto*-nonitol **5** was found to possess the highest latent heat of fusion of all investigated sugar alcohols with a value of about 390 J/g, but again, decomposition at temperatures above ∼260 °C was observed, indicating a temperature limit as applicable PCMs for structures of the discussed nature. Thermal instability at such high temperatures is not unexpected, especially considering that polyhydroxylated compounds will basically always contain some residual water. At these high temperatures, the equilibrium of H_2_O shifts more to the side of H_3_O^+^ and OH^–^, which might favor the decomposition of an organic molecule by acid catalysis. This was demonstrated by Yamaguchi et al.^37,38^ who investigated the dehydration and further degradation of the C6 sugar alcohols d-mannitol (**1**) and d-sorbitol, the corresponding alcohol from d-glucose, in high-temperature liquid water (250–300 °C) under inert atmosphere (Ar) without any acid catalysts, showing the thermal instability of sugar alcohols in the presence of water.

Nevertheless, our research contributes to a broader understanding of PCMs and their properties, regarding the relationship between structural features (stereochemistry and chain length) and thermal behavior. Additionally, we shed light on the thermal instability of sugar alcohols with melting points exceeding 240 °C, underscoring the inherent challenges associated with utilizing organic compounds as PCMs when facing this temperature window. Since high thermal stability is one of the most important physical properties for a material considered as a PCM, decomposition at temperatures close to the melting point is an undesirable characteristic for further application, despite efforts in the development of encapsulation techniques for natural sugar alcohols^15,39,40^ to overcome thermal stability issues.

## Conclusions

In conclusion, a synthetic route for higher-carbon sugar alcohols with high control of stereoselectivity for the introduced hydroxyl groups was developed, allowing the synthesis of *galacto*-configured sugar alcohols with an even number of carbon atoms in the backbone and their C2-epimers. The IMA was shown to be a useful tool in the synthesis of higher-carbon sugar alcohols or sugar species in general, especially with the implementation of a two-step Grignard-type protocol for the elongation of less reactive sugars that allowed the synthesis of the *galacto*-decitol **6** with 10 carbon atoms. In total, six sugar alcohols were synthesized, two of them fulfilling all of the stated criteria with 8 and 10 carbons in the backbone (*galacto*-octitol **4** and *galacto*-decitol **6**), their C2-epimers that bear one 1,3-*syn*-relationship (*epi*-*galacto*-octitol ***epi*****-4** and *epi*-*galacto*-octitol ***epi*****-6**), and additionally two sugar alcohols with all hydroxyl groups in 1,3-*anti*-relationship but with an odd number of carbon atoms (*galacto*-heptitol **3** and *galacto*-nonitol **5**). Further, their thermal properties were investigated regarding their potential use as PCMs. The influence of the distribution of the hydroxyl groups and length of the carbon chain could be shown with the melting points of the synthesized sugar alcohols. For the *galacto*-configured sugar alcohols, latent heats of fusion above 300 J/g were observed, which is considered as outstandingly high values among organic materials investigated as PCMs. However, especially the sugar alcohols with melting points above 240 °C were found to be thermally unstable in the melting process and are therefore unsuitable in the application as PCMs despite their capability of storing rather high energy amounts in the form of latent heat.

## Experimental Section

### General

The used reagents and solvents were purchased from commercial sources with a purity of >95%, unless noted differently, and used without further purification. Water-free solvents were available from a PureSolv solvent purification system by Innovative Technology or commercial sources that were stated as water-free and stored in bottles with a septum and over molecular sieves. Dowex-H^+^ resin was washed with the respective solvent before use.

Thin-layer chromatography (TLC) for reaction monitoring and fraction analysis from column chromatography was performed with silica gel 60 F254 plates or HPTLC-plates (silica gel 60 F254 with concentration zone 20 × 2.5 cm). Visualization of the spots was done using UV light (254 nm) or via heat staining the plates with anisaldehyde solution (180 mL EtOH, 10 mL anisaldehyde, 10 mL H_2_SO_4_ (conc.), 2 mL AcOH), permanganate solution (3.0 g KMnO_4_, 20.0 g K_2_CO_3_, 250 mg KOH, 300 mL H_2_O), or cerium molybdate (“Mostain”, 21.0 g (NH_4_)_6_Mo_7_O_24_·2H_2_O, 1.0 g Ce(SO_4_), 31 mL H_2_SO_4_ (conc.), 500 mL H_2_O).

For flash column chromatography, columns were packed with silica gel from Merck with a pore size of 40–63 μm. Purification was done either by hand column or on a Büchi Pure C-850 FlashPrep System. Light petroleum is referred to as LP.

Liquid chromatography-mass spectrometry (LC-MS) analysis was performed on a Nexera X2 UHPLC system (Shimadzu, Kyoto, Japan) comprised of LC-30AD pumps, a SIL-30AC autosampler, a CTO-20AC column oven, and a DGU-20A5/3 degasser module. Detection was accomplished by an SPD-M20A photo diode array and an LC-MS-2020 mass spectrometer. Separations were either performed using a Waters XSelect CSH C18 2.5 μm (3.0 × 50 mm) Column XP at 40 °C, a flow rate of 1.7 mL/min, and with UHPLC grade water and acetonitrile containing 0.1% formic acid as the mobile phase, or a Waters XBridge BEH Amide 2.5 μm (3.0 × 50 mm) Column XP at 40 °C, a flow rate of 1.3 mL/min, and with UPLC grade water (pH 8.5, 2.5 mM NH_4_COOH) and acetonitrile as the mobile phase.

Accurate mass analysis was performed on an Agilent 6230 AJS ESI-TOF mass spectrometer with ESI ionization method or Q Exactive Focus, ESI, FIA injection, mobile phase 18% MeCN with 0.1% formic acid.

^1^H NMR and ^13^C NMR spectra were recorded at ambient temperature in the solvent indicated using a Bruker Avance Ultra Shield 400 MHz and an Avance III HD 600 MHz spectrometer. Processing of the data was performed with standard software, and all spectra were calibrated to the solvent residual peak. Chemical shifts (δ) are reported in ppm, coupling constants (*J*) in hertz (Hz), and multiplicities are assigned as s = singlet, d = doublet, t = triplet, q = quartet, m = multiplet, etc. All assignments are based on 2D-spectra (COSY, phase-sensitive HSQC, HMBC— depending on the molecule). Within the assignments, PNB is used as the abbreviation for 4-nitrobenzoyl.

Melting points were recorded using a BÜCHI Melting Point B 545 with a 40%/90% threshold and a heating rate of 1.0 °C/min or a Kofler-type Leica Galen III micro-hot stage microscope.

Ozone-enriched oxygen was generated using a Triogen LAB2B Ozone generator.

Simultaneous thermal analysis (STA) including differential scanning calorimetry (DSC) and thermogravimetric analysis (TG) measurements were performed on a Netzsch STA 449 F1 *Jupiter* under a nitrogen atmosphere with a constant gas flow rate of 40 mL/min and a heating and cooling rate of 10 °C/min if not stated otherwise. Samples were measured using Al pans (25 μL) with a hole in the lid. Samples were heated to approximately 30–40 °C above their melting points to prevent any decomposition at higher temperature. DSC measurements were performed on a Netzsch DSC 204 F1 *Phoenix* with the same measuring parameters but using closed Al pans (25 μL).

Compounds were named according to IUPAC systematic standards, in general. When it comes to higher-carbon sugar species (more than six carbon atoms), names were generated by dividing the sugar species into groups of up to four chiral centers, consequently starting from the chiral center next to the former reducing end (on the right for all displayed structures). To these groups, configurational prefixes were assigned, and the name was built up by putting the prefix of the group that is farthest from the right end (C1) first. This group may contain less than four carbon atoms. Numbering of compounds was performed in the same way, always starting with 1 at the former reducing end as shown in the exemplary structures below.
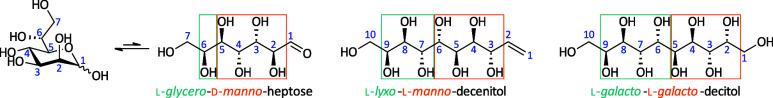


### Toward the Octitols

#### 3,4,5,6,7,8-Hexa-*O*-benzyl-1,2-dideoxy-l-*glycero*-d-*manno*-oct-1-enitol (**13**)

Enitol **7** (1.50 g, 7.20 mmol, 1.00 equiv) was suspended in dry DMF (30 mL), and NaH (60% dispersion in paraffin oil, 4.33 g, 108 mmol, 15.0 equiv) was added portionwise to the stirred mixture under ice-bath cooling (formation of H_2_). The reaction mixture was stirred until no further formation of H_2_ was observed, and then benzyl bromide (10.5 mL, 15.1 g, 86.5 mmol, 12.0 equiv) was added dropwise, which led to the formation of a beige precipitate. After complete addition of BnBr, the cooling bath was removed and *n*-Bu_4_NI (1.4 g, 3.6 mmol, 0.50 equiv) was added and stirring was continued overnight. TLC (LP/EtOAc 2:1) indicated complete conversion, and excessive reagent was quenched by the addition of MeOH (9 mL) and aq. NH_4_Cl (10%, 90 mL). After dilution with Et_2_O (100 mL), phases were separated, and the aq. phase was extracted with Et_2_O (3 × 100 mL). The combined org. phase was washed with H_2_O (3 × 50 mL) and brine. After drying over anhydrous Na_2_SO_4_, the solvent was removed *in vacuo*. The residue was taken up in acetonitrile (130 mL), washed with *n*-hexane (3 × 60 mL), and vaporized again, giving a yellow oil (6.76 g). The crude product was purified via flash column chromatography (250 g SiO_2_, LP/EtOAc 10:1 → 3:1) to yield the desired product as a colorless oil (5.34 g, 99%). ^**1**^**H NMR (600 MHz, CDCl**_**3**_**)** δ 7.33–7.18 (m, 30H, 30 × CH Ph), 5.94 (ddd, *J* = 17.4, 10.4, 8.0 Hz, 1H, H2), 5.40–5.22 (m, 2H, H1a&H1b), 4.75 (d, *J* = 7.4 Hz, 1H, C***H***H-Ph), 4.73 (d, *J* = 8.2 Hz, 1H, C***H***H-Ph), 4.67–4.54 (m, 6H, 6 × C***H***H-Ph), 4.51 (d, *J* = 11.7 Hz, 1H, H8a), 4.45 (d, *J* = 12.0 Hz, 1H, C***H***H-Ph), 4.45 (d, *J* = 11.9 Hz, 1H, C***H***H-Ph), 4.10 (d, *J* = 11.7 Hz, 1H, H8b), 4.08–4.02 (m, 2H, H3, H7), 3.99–3.90 (m, 3H, H5, H6, H4), 3.70 (d, *J* = 5.3 Hz, 2H, C***H***_2_-Ph); ^**13**^**C{**^**1**^**H}-NMR (151 MHz, CDCl**_**3**_**)** δ 139.22, 139.19, 139.0, 138.9, 138.8, 138.4 (6 × PhC1), 136.1 (C2), 128.6–127.2 (30 × PhCH), 119.8 (C1), 81.3 (C3), 81.2 (C4), 78.79 (2 × CH), 78.77 (CH), 74.0, 73.9, 73.34, 73.31, 73.0, 70.9 (6 × ***C***H_2_–Ph), 70.0 (C8) ppm; **HRMS (ESI)***m*/z [M + Na]^+^ calc. for C_50_H_52_NaO_6_: 771.3662, found: 771.3685.

#### 1,2-Dideoxy-3,4:5,6:7,8-tri-*O*-isopropylidene-l-*glycero*-d-*manno*-oct-1-enitol (**14**)

Enitol **7** (800 mg, 3.84 mmol, 1.00 equiv) was suspended in dry DMF (2 mL), and 2,2-dimethoxypropane (2.40 g, 2.83 mL, 23.1 mmol, 6 equiv) and *p*-TSA·H_2_O (15 mg, 0.077 mmol, 2.0 mol %) were added under stirring at rt. Then, the mixture was heated to 50 °C and stirred at this temperature. Reaction monitoring via TLC after 6 h (LP/Et_2_O 2:1) showed full consumption of the starting material but several more nonpolar species. Another portion of *p*-TSA·H_2_O (15 mg) was added, and stirring at 50 °C was continued overnight, but reaction monitoring did not show further conversion to the most nonpolar species (targeted product). The reaction mixture was quenched after 48 h in total by the addition of TEA (39 mg, 53 μL, 0.10 equiv). Coevaporation with toluene (4 × 4 mL) gave a brown oil as a crude material (1.30 g). The pure product **14** was obtained via flash column chromatography (130 SiO_2_, LP/EtOAc 6:1) as a colorless oil (233 mg, 19%). Next to the product, mixed fractions (102 mg) with a second octenitol species with also three isopropylidene groups and also pure fractions of this side product (130 mg) were isolated. The structure of this second isomer could not be fully clarified by NMR analysis (see the SI for analytical data). ^**1**^**H NMR (400 MHz, CDCl**_**3**_**)** δ 5.90 (ddd, *J* = 17.4, 10.7, 5.0 Hz, 1H, H2), 5.33 (dt, *J* = 17.7, 1.7 Hz, 1H, H1a), 5.23–5.17 (m, 1H, H1b), 4.16 (q, *J* = 6.8 Hz, 1H, H7), 4.11 (ddt, *J* = 6.6, 5.0, 1.6 Hz, 1H, H3), 4.00–3.85 (m, 2H, H8a, H8b), 3.87–3.76 (m, 2H, H5, H4), 3.64 (dd, *J* = 8.1, 6.7 Hz, 1H, H6), 1.42 (s, 6H, 2 × CH_3_), 1.40 (s, 3H, CH_3_), 1.38 (s, 3H, CH_3_), 1.34 (s, 3H, CH_3_), 1.32 (s, 3H, CH_3_); ^**13**^**C{**^**1**^**H}-NMR (101 MHz, CDCl**_**3**_**)** δ 135.9 (C2), 116.4 (C1), 109.6 (***C***(CH_3_)_2_), 101.1 (***C***(CH_3_)_2_), 101.0 (***C***(CH_3_)_2_), 76.8 (C7), 71.9 (C4), 70.7 (C6), 69.9 (C3), 68.8 (C5), 65.4 (C8), 26.6, 25.9, 24.7, 24.5, 23.9, 23.8 (6 × CH_3_) ppm. With the available HRMS equipment, no ionization of compound **14** could be achieved.

#### 3,4,5,6,7,8-Hexa-*O*-benzyl-l-*threo*-d-*talo*-octitol (***epi*****-16**)

AD-mix-β (×3) (3.64 g, 1.40 g/mmol starting material) (for preparation, see the SI) was suspended in *t*-BuOH/H_2_O (1:1, 5.0 mL), and MsNH_2_ (742 mg, 7.80 mmol, 3.00 equiv) was added under vigorous stirring. After 1 h, the protected octenitol **13** (2.04 g, 2.72 mmol, 1.00 equiv) was added as a solution in DCM (2.5 mL). After 12 h, TLC (LP/EtOAc 2:1) indicated complete conversion and the reaction mixture was quenched by the addition of Na_2_S_2_O_5_, until further addition of Na_2_S_2_O_5_ no longer resulted in foaming. After dilution with H_2_O (50 mL) and DCM (50 mL), the phases were separated, and the aq. phase was extracted with DCM (3 × 100 mL). The pooled organic phase was washed with brine, dried over Na_2_SO_4_, and vaporized, giving a mixture of *talo* and *galacto*-isomer ***epi*****-16**/**16** (∼20:1 *talo*/*galacto*, diastereomeric ratio calculated by analysis of the corresponding ^13^C NMR spectrum upon deprotection of an analytical sample via catalytic hydrogenation (see the SI)) as a yellow oil (2.01 g, 99%) that was used without further purification. Analytical data for major isomer ***epi*****-16**: ^**1**^**H NMR (600 MHz, CDCl**_**3**_**)** δ 7.33–7.22 (m, 28H, 28 × PhCH), 7.17–7.12 (m, 2H, 2 × PhCH), 4.71 (m, 2H, 2 × C***H***H-Ph), 4.69–4.59 (m, 4H, CH_2_–Ph, 2 × C***H***H-Ph), 4.58 (d, *J* = 11.5 Hz, 2H, CH_2_–Ph), 4.47–4.42 (m, 3H, C***H***H-Ph, CH_2_–Ph), 4.32 (d, *J* = 11.5 Hz, 1H, C***H***H-Ph), 4.08 (dd, *J* = 5.4, 2.9 Hz, 1H, CH), 3.99–3.91 (m, 3H, 3 × CH), 3.77 (dt, *J* = 7.2, 4.3 Hz, 1H, CH), 3.75–3.67 (m, 4H, CH, H8, H1a), 3.59 (dd, *J* = 11.4, 4.7 Hz, 1H, H1b).^**13**^**C{**^**1**^**H}-NMR (151 MHz, CDCl**_**3**_**)** δ 138.7, 138.6, 138.5, 138.24, 138.23, 138.18 (6 × PhC1), 128.6–127.6 (30 × PhCH), 81.0 (CH), 80.0 (CH), 79.5 (CH), 79.3 (CH), 78.8 (CH), 74.6, 73.9, 73.8, 73.5, 73.2, 73.0 (***C***H_2_–Ph), 71.3 (CH), 70.6 (C8), 64.0 (C1) ppm; **HRMS (ESI)***m*/z [M + Na]^+^ calc. for C_50_H_54_NaO_8_: 805.3716, found: 805.3742.

#### 3,4,5,6,7,8-Hexa-*O*-benzyl-1,2-di-*O*-(4-nitrobenzoyl)-l-*threo*-d-*galacto*-octitol (**18**)

PPh_3_ (3.37 g, 12.8 mmol, 5.00 equiv) was weighed in a flame-dried Schlenk flask, and Schlenk technique was applied. Dry THF (12.5 mL) was added, the mixture was cooled to 0 °C using an ice-bath, and DIAD (2.69 mL, 12.8 mmol, 5.00 equiv) was added dropwise, which led to the formation of a beige precipitate. Additional dry THF (5 mL) was added to guarantee efficient stirring. The starting material ***epi*****-16**/**16** (∼20:1, 2.01 g, 2.57 mmol, 1.00 equiv., predried by azeotropic evaporation with dry toluene (3 × 6 mL)) was added to the reaction mixture as a solution in THF (10 mL). Then, 4-nitrobenzoic acid (2.15 g, 12.8 mmol, 5.00 equiv) was added and the reaction mixture was allowed to slowly warm up to rt and further heated to 45 °C. TLC analysis (LP/EtOAc) after 24 h indicated complete conversion of the starting material. The solvent was evaporated, and the residue was taken up in cold Et_2_O. Upon stirring under ice-bath cooling, a white precipitate was formed that was removed by filtration. The filtrate was diluted with further Et_2_O, and the org. phase (in total 150 mL) was washed with sat. aq. NaHCO_3_ (3 × 80 mL) and brine (30 mL). After drying over Na_2_SO_4_, the solvent was removed *in vacuo*, giving a yellow oil as a crude material (8.79 g). This was purified via flash column chromatography (LP/EtOAc 10:1 → 5:1), giving the targeted product (1.69 g, 61%) as a mixture of the two diastereomers (∼20:1 *galacto*/*talo*, diastereomeric ratio calculated by analysis of ^13^C NMR spectrum upon deprotection of an analytical sample via ester hydrolysis and catalytic hydrogenation—see compound **4**, Strategy A) as a slightly yellow oil. Analytical data for major isomer **18**: ^**1**^**H NMR (600 MHz, CDCl**_**3**_**)** δ 8.18–8.13 (m, 2H, 2 × PNBCH), 8.02–7.94 (m, 6H, 6 × PNBCH), 7.33–7.04 (m, 30H, 30 × PhCH), 5.95 (ddd, *J* = 8.3, 3.7, 2.6 Hz, 1H, H2), 4.75 (d, *J* = 11.7 Hz, 1H, C***H***H-Ph), 4.72–4.65 (m, 3H, 3 × C***H***H-Ph), 4.63 (d, *J* = 11.5 Hz, 1H, CH_2_–Ph), 4.55 (d, *J* = 11.4 Hz, 1H, C***H***H-Ph), 4.54–4.48 (m, 3H, CH_2_–Ph, H1a), 4.47–4.40 (m, 2H, CH_2_–Ph), 4.34 (d, *J* = 11.6 Hz, 1H, C***H***H-Ph), 4.17 (dd, *J* = 11.6, 3.7 Hz, 1H, H1b), 4.12–4.08 (m, 1H, CH), 4.06–3.99 (m, 4H, 3 × CH, H3), 3.70 (h, *J* = 5.5, 5.1 Hz, 2H, H8); ^**13**^**C{**^**1**^**H}-NMR (151 MHz, CDCl**_**3**_**)** δ 164.25, 164.19 (2 × C=O), 150.6, 150.4 (2 × PNBC4), 138.8, 138.6, 138.4, 138.19, 138.17, 137.7 (6 × PhC1), 135.4, 135.1 (2 × PNBC1), 130.82, 130.80 (2 × PNBCH), 128.5–127.5 (30 × PhCH), 123.6, 123.5 (2 × PNBCH), 79.7, 79.5, 79.0, 78.3 (4 × CH), 77.1 (C3), 74.6, 74.1, 73.5, 73.42, 73.41, 72.6 (6 × ***C***H_2_–Ph), 71.9 (C2), 70.5 (C8), 65.4 (C1) ppm; **HRMS (ESI)***m*/z [M + Na]^+^ calc. for C_64_H_60_N_2_NaO_14_: 1103.3942, found: 1103.3975.

#### 3,4,5,6,7,8-Hexa-*O*-benzyl-l-*threo*-d-*galacto*-octitol (**16**)

The isomer mixture **18**/***epi*****-18** from the Mitsunobu reaction (1.18 g, 1.09 mmol, 1.00 equiv) was dissolved in THF (18 mL), and LiOH (aq., 1 M, 18 mL, 1.7 equiv) was added under stirring at rt. After 1.5 h, TLC analysis (LP/EtOAc 2:1) indicated full conversion to a single spot. The solvent was evaporated, and the residue was taken up in DCM (50 mL) and water (30 mL). The phases were separated, and the aq. phase was extracted with DCM (3 × 30 mL). The pooled organic phases were washed with sat. aq. NaHCO_3_ (2 × 30 mL) and brine. After drying over Na_2_SO_4_, the solvent was removed *in vacuo*, giving the product mixture **16**/***epi*****-16** (∼20:1) as a yellow oil (759 mg, 89%). This material was used in the next step without further purification. Analytical data for major isomer **16**: ^**1**^**H NMR (600 MHz, CDCl**_**3**_**)** δ 7.33–7.22 (m, 28H, 28 × PhCH), 7.14–7.12 (m, 2H, 2 × PhCH), 4.76–4.58 (m, 8H, 4 × CH_2_–Ph), 4.44 (s, 2H, C***H***_2_-Ph), 4.35 (d, *J* = 11.6 Hz, 1H, C***H***H-Ph), 4.18 (d, *J* = 11.6 Hz, 1H, C***H***H-Ph), 4.13 (dd, *J* = 6.2, 4.0 Hz, 1H, H4), 3.94 (dd, *J* = 6.2, 3.3 Hz, 1H, H5), 3.92–3.89 (m, 2H, H6, H7), 3.80 (td, *J* = 5.7, 2.2 Hz, 1H, H2), 3.76–3.71 (m, 2H, H8), 3.65 (dd, *J* = 4.0, 2.2 Hz, 1H, H3), 3.56 (dd, *J* = 11.1, 6.1 Hz, 1H, H1a), 3.41 (dd, *J* = 11.1, 5.2 Hz, 1H, H1b); ^**13**^**C{**^**1**^**H}-NMR (151 MHz, CDCl**_**3**_**)** δ 138.8, 138.6, 138.4, 138.3, 138.2, 138.1 (6 × PhC1), 128.6–127.5 (30 × PhCH), 80.6 (C4), 79.6 (C5), 79.2 (C6/C7), 78.9 (C6/C7), 77.5 (C3), 75.4, 73.9, 73.6, 73.5, 73.4, 72.3 (6 × ***C***H_2_–Ph), 71.4 (C2), 70.9 (C8), 64.0 (C1). ppm; **HRMS (ESI)***m*/z [M + Na]^+^ calc. for C_50_H_54_NaO_8_: 805.3716, found: 805.3736.

#### 1,2,3,4,5,6,7,8-Octa-*O*-benzyl-l-*threo*-d-*galacto*-octitol (**19**)

The diastereomeric mixture **16**/***epi*****-16** (∼20:1, 759 mg, 0.969 mmol, 1.00 equiv) was suspended in dry DMF (4 mL), and NaH (60% dispersion in paraffin oil, 194 mg, 4.85 mmol, 5.00 equiv) was added portionwise to the stirring mixture under ice-bath cooling (formation of H_2_). The reaction mixture was stirred until no further formation of H_2_ was observed; then, benzyl bromide (675 mg, 3.87 mmol, 4.00 equiv) was added dropwise, which led to the formation of a beige precipitate. After complete addition of BnBr, the cooling bath was removed and *n*-Bu_4_NI (0.18 g, 0.49 mmol, 0.50 equiv) was added. After 2 h, TLC analysis (LP/EtOAc 2:1) indicated complete conversion and excessive reagent was quenched by the addition of MeOH (0.6 mL) and aq. NH_4_Cl (10%, 30 mL). After dilution with Et_2_O (50 mL), phases were separated, and the aq. phase was extracted with Et_2_O (3 × 70 mL). The combined org. phase was washed with H_2_O (3 × 20 mL) and brine (20 mL). After drying over anhydrous Na_2_SO_4_, the solvent was removed *in vacuo*. The residue was taken up in acetonitrile (100 mL), washed with *n*-hexane (2 × 40 mL), and evaporated again, giving a yellow oil (865 mg). The crude product was purified via column chromatography (180 g SiO_2_, LP/EtOAc 10:1 → 3:1 (slow gradient)) to yield the desired product **19** as a colorless oil (671 mg, 72%). ^**1**^**H NMR (400 MHz, CDCl**_**3**_**)** δ 7.36–7.15 (m, 40H, 40 × PhCH), 4.70 (dd, *J* = 11.7, 1.4 Hz, 4H, 4 × C***H***H-Ph), 4.66–4.56 (m, 4H, 4 × C***H***H-Ph), 4.53 (d, *J* = 2.0 Hz, 4H, 2 × CH_2_–Ph), 4.46–4.34 (m, 4H, 2 × CH_2_–Ph), 4.18 (d, *J* = 5.1 Hz, 2H, 2 × CH), 4.05–3.98 (m, 4H, 4 × CH), 3.70 (d, *J* = 5.1 Hz, 4H, H1a&H1b, H8a&H8b); ^**13**^**C{**^**1**^**H}-NMR (101 MHz, CDCl**_**3**_**)** δ 139.4, 139.1, 138.9, 138.5 (8 × PhC1), 128.5–127.2 (40 × PhCH), 78.71 (2 × CH), 78.68 (2 × CH), 78.44 (2 × CH), 73.3, 73.1, 73.0 (8 × ***C***H_2_–Ph), 71.0 (C1, C8) ppm; **HRMS (ESI)***m*/z [M + H]^+^ calc. for C_64_H_67_O_8_: 963.4836, found: 963.4855.

#### l-*Threo*-d-*galacto*-octitol (**4**): Strategy A

Step 1: ester cleavage: the diastereomeric mixture after the Mitsunobu reaction **18**/***epi*****-18** (164 mg, 0.152 mmol, 1.00 equiv) was taken up in MeOH (HPLC grade, 2.0 mL), and NaOMe (30% in MeOH) was added dropwise under stirring at rt until pH was about 10. After 2 h, LC-MS indicated complete conversion and the reaction mixture was neutralized by the addition of Dowex-H^+^. After filtration and evaporation of the filtrate, the residue was taken up in DCM (40 mL) and extracted with sat. aq. NaHCO_3_ (2 × 10 mL) and brine. After drying over anhydrous Na_2_SO_4_, the solvent was removed *in vacuo*, giving the crude product (120 mg) as a yellow oil. Step 2: catalytic hydrogenation: the observed material was taken up in MeOH (HPLC grade, 1.0 mL), and Pd/C (10 wt %, 12 mg, 7 mol %) was added under an Ar atmosphere followed by a drop of 2 N HCl. The atmosphere was changed to hydrogen using a balloon, and the reaction mixture was stirred at rt for 14 h, as LC-MS indicated complete conversion. The mixture was diluted with H_2_O, filtered over celite (H_2_O washed), and the filter pad was washed with H_2_O. The filtrate was evaporated to dryness, giving a colorless solid (27 mg, 73% over 2 steps). According to ^13^C NMR, the ratio **4**/***epi*****-4** was 20:1. Attempts to obtain pure *galacto*-octitol **4** via recrystallization from MeOH, H_2_O, or MeOH/H_2_O mixtures failed.

#### l-*Threo*-d-*galacto*-octitol (**4**): Strategy B

Octabenzyl octitol **19** (215 mg, 0.223 mmol, 1.00 equiv) was placed under an Ar atmosphere and dissolved in MeOH/EtOAc (1:1, 2 mL). Pd/C (10 wt %, 45 mg, 19 mol %) was added, followed by a drop of acetic acid. The atmosphere was changed to H_2_ using a balloon, and the reaction mixture was stirred at 50 °C overnight. LC-MS analysis indicated complete conversion to the desired product. The mixture was filtered over celite (H_2_O washed), and the filter pad was washed with H_2_O. The filtrate was evaporated to dryness, giving an off-white solid. This was again dissolved in H_2_O (70 mL), syringe-filtered, and lyophilized, giving the targeted product **4** (50 mg, 93%) as a colorless solid, pure according to ^1^H NMR. For STA-measurements, small samples (∼10–15 mg) were recrystallized from MeOH/H_2_O (4:1). **mp** 221–223 °C (H_2_O) (lit.^20^ 233–236 °C (H_2_O)); ^**1**^**H NMR (600 MHz, D**_**2**_**O)** δ 4.01–3.98 (m, 2H, H2, H7), 3.95 (d, *J* = 9.3 Hz, 2H, H4, H5), 3.72–3.68 (m, 6H, H1a&H1b, H8a&H8b, H3, H6); ^**13**^**C{**^**1**^**H}-NMR (151 MHz, D**_**2**_**O)** δ 70.3 (C2, C7), 69.3 (C3, C6), 68.2 (C4, C5), 63.3 (C1, C8) ppm; **HRMS (ESI)***m*/z [M – H]^−^ calc. for C_8_H_17_O_8_: 241.0923, found: 241.0932.

#### 1,2,3,4,5,6,7,8-Octa-*O*-benzyl-l-*threo*-d-*talo*-octitol (***epi*****-19**)

The diastereomeric mixture ***epi*****-16**/**16** (∼20:1, 1.03 mg, 1.31 mmol, 1.00 equiv) was suspended in dry DMF (5 mL), and NaH (60% dispersion in paraffin oil, 262 mg, 6.55 mmol, 5.00 equiv) was added portionwise to the stirring mixture under ice-bath cooling (formation of H_2_). The reaction mixture was stirred until no further formation of H_2_ was observed; then, benzyl bromide (913 mg, 5.23 mmol, 4.00 equiv) was added dropwise, which led to the formation of a beige precipitate. After complete addition of BnBr, the cooling bath was removed and *n*-Bu_4_NI (0.25 g, 0.66 mmol, 0.50 equiv) was added and the reaction mixture was stirred overnight. TLC analysis (LP/EtOAc 2:1) indicated complete conversion, and excessive reagent was quenched by the addition of MeOH (0.6 mL) and aq. NH_4_Cl (10%, 30 mL). After dilution with Et_2_O (50 mL), phases were separated, and the aq. phase was extracted with Et_2_O (3 × 70 mL). The combined org. phase was washed with H_2_O (3 × 20 mL) and brine (20 mL). After drying over anhydrous Na_2_SO_4_, the solvent was removed *in vacuo*. The residue was taken up in acetonitrile (100 mL), washed with *n*-hexane (2 × 40 mL), and vaporized again, giving a yellow oil (1.17 g). The crude product was purified via column chromatography (180 g SiO_2_, LP/EtOAc 10:1 → 3:1, slow gradient) to yield the desired product ***epi*****-19** as a colorless oil (869 mg, 69%). ^**1**^**H NMR (600 MHz, CDCl**_**3**_**)** δ 7.32–7.20 (m, 38H, 38 × PhCH), 7.10 (dd, *J* = 6.6, 3.0 Hz, 2H, 2× PhCH), 4.78–4.49 (m, 11H, CH_2_–Ph), 4.46–4.39 (m, 3H, CH_2_–Ph), 4.33–4.28 (m, 2H, CH_2_–Ph), 4.24 (d, *J* = 12.0 Hz, 1H, C***H***H-Ph), 4.15 (dd, *J* = 5.5, 4.0 Hz, 1H, CH), 4.07–3.98 (m, 4H, 4 × CH), 3.87 (td, *J* = 5.4, 3.9 Hz, 1H, CH), 3.73 (dd, *J* = 10.5, 2.7 Hz, 1H, H1a/H8a), 3.66 (dd, *J* = 10.5, 5.4 Hz, 1H, H1b/H8b), 3.58 (qd, *J* = 10.4, 4.7 Hz, 2H, H1/H8); ^**13**^**C{**^**1**^**H}-NMR (151 MHz, CDCl**_**3**_**)** δ 139.43, 139.42, 139.1, 139.0, 138.9, 138.8, 138.7, 138.5 (8 × PhC1), 128.5–127.2 (40 × PhCH), 80.0, 79.8, 79.5, 79.2, 79.1, 78.7 (6 × CH), 74.3, 73.6, 73.4, 73.3, 73.1, 73.0, 72.8, 72.3 (8 × ***C***H_2_–Ph), 70.7 (C1/C8), 70.5 (C1/C8) ppm; **HRMS (ESI):***m*/z [M + H]^+^ calc. for C_64_H_67_O_8_: 963.4836, found: 963.3852.

#### l-*Threo*-d-*talo*-octitol (***epi*****-4**)

Octabenzyl octitol ***epi*****-19** (220 mg, 0.228 mmol, 1.00 equiv) was placed under an Ar atmosphere and dissolved in MeOH/EtOAc/H_2_O (4:2:1, 2 mL). Pd/C (10 wt %, 46 mg, 19 mol %) was added, followed by a drop of acetic acid. The atmosphere was changed to H_2_ using a balloon, and the reaction mixture was stirred at 50 °C overnight. LC-MS analysis indicated complete conversion to the desired product. The mixture was filtered over celite (H_2_O washed), and the filter pad was washed with H_2_O. The filtrate was evaporated to dryness, giving an off-white solid. This was triturated with *i*-PrOH and MeOH (separation via centrifugation) giving ***epi*****-4** (40 mg, 71%) as a colorless solid, pure according to ^1^H NMR. For STA-measurements, small samples (∼10–15 mg) were recrystallized from MeOH/H_2_O (4:1). **mp** 165–166 °C (H_2_O) (lit.^20^ 153–154.5 °C (EtOH/H_2_O)); ^**1**^**H NMR (600 MHz, D**_**2**_**O)** δ 4.00–3.90 (m, 4H, 4 × CH), 3.85–3.81 (m, 2H, CH, H1a/H8a), 3.72–3.66 (m, 4H, CH, H1a/H8a, H1b, H8b); ^**13**^**C{**^**1**^**H}-NMR (151 MHz, D**_**2**_**O)** δ 72.8, 71.5, 70.2, 69.4, 69.1, 68.5 (6 × CH), 63.3 (C1/C8), 61.9 (C1/C8) ppm; **HRMS (ESI)***m*/z [M – H]^−^ calc. for C_8_H_17_O_8_: 241.0923, found: 241.0940.

### Toward the Decitols

#### 1,2-Dideoxy-l-*lyxo*-l-*manno*-dec-1-enitol (**10**)

Step 1: IMA: based on a literature protocol,^34^ indium (1.85 g, 16.1 mmol, 2.00 equiv) was weighed in a flame-dried Schlenk flask and Schlenk technique was applied. Anhydrous THF (21.0 mL) was added, and the mixture was cooled to 0 °C using an ice-bath. 3-Bromopropenyl pivalate (**21**) (5.34 g, 28.5 mmol, 3.00 equiv) was added dropwise to the vigorously stirred mixture. After 15 min, the ice-bath was removed, and the suspension was allowed to warm to room temperature and stirred for another 30 min. Then, l-*glycero*-d-*manno*-heptose (**11**) (1.69 g, 8.05 mmol, 1.00 equiv) was added to the preformed reagent as a solution in acidic phthalate buffer (pH 3, 3.5 mL). For the buffer, in a 200 mL volumetric flask, 50.0 mL of 0.2 M potassium hydrogen phthalate and 22.3 mL of 0.2 M HCl were combined and diluted with water to the desired volume. Then, pH was adjusted to 3.0 using a pH-electrode. To guarantee efficient stirring during the reaction, a further THF (5.0 mL) was added 30 min after the addition of the starting material. As TLC (CHCl_3_/MeOH/H_2_O 14:7:1) indicated full conversion of the starting material (after 45 min), the reaction mixture was diluted with MeOH and H_2_O and filtered. The filtrate was concentrated in vacuo, giving an off-white solid. Step 2: acetylation: this material was taken up in pyridine (45 mL), and acetic anhydride (26.9 mL, 29.1 g, 282 mmol, 35 equiv) was added to the stirring mixture under ice-bath cooling. After 10 min, the ice-bath was removed, and a spatula of DMAP was added. After 4 h, LC-MS indicated complete conversion to the fully protected decenitol. Excessive reagent was quenched by the addition of MeOH (12 mL) under ice-bath cooling, and the mixture was stirred for further 30 min. After diluting with EtOAc (200 mL), the organic phase was extracted with ice-cold 2 N HCl (1 × 150 mL, 2 × 100 mL—until aq. phase remained acidic) and combined HCl-phase once extracted with EtOAc (50 mL). The pooled organic phase was washed with H_2_O (30 mL), aq. sat. NaHCO_3_ (2 × 30 mL), and brine. After drying over anhydrous Na_2_SO_4_, the solution was evaporated to dryness. Step 3: deacetylation: the residue was taken up in MeOH (HPLC grade, 40 mL), and NaOMe (30% in MeOH, ∼3 mL) was added dropwise under stirring at rt until pH was about 10. Reaction monitoring via LC-MS after 3 h showed full conversion. The reaction mixture was neutralized by the addition of Dowex-H^+^ resin (MeOH washed), H_2_O was added to dissolve the formed enitol, and the solution was filtered. Evaporation of the solvent gave a beige solid matter (2.5 g) that was a mixture of diastereomers in the ratio *lyxo*/*xylo/ribo* = 71:19:10 (^1^H NMR, tentative assignment in analogy to isolated isomers from the analogous elongation of lyxose^26^, see the SI). The solid was then triturated with *i*-PrOH (30 mL), and the remaining solid was isolated by centrifugation. The residue was washed twice with MeOH (2 × 20 mL), giving a light beige solid (950 mg) that was finally recrystallized from H_2_O (10 mL). The formed crystalline solid was separated via centrifugation, giving pure l-*lyxo*-l-*manno*-decenitol **10** (730 mg, 34%). **mp** 220.4–224.2 °C (H_2_O); ^**1**^**H NMR (600 MHz, D**_**2**_**O)** δ 6.03 (ddd, *J* = 17.3, 10.5, 6.9 Hz, 1H, H2), 5.39 (dt, *J* = 17.3, 1.2 Hz, 1H, H1a), 5.32 (dt, *J* = 10.5, 1.1 Hz, 1H, H1b), 4.23–4.17 (m, 1H, H3), 4.00 (td, *J* = 6.5, 6.0, 1.4 Hz, 1H, H9), 3.96 (d, *J* = 9.1 Hz, 3H, H5, H6, H7), 3.78 (d, *J* = 8.1 Hz, 1H, H4), 3.73–3.68 (m, 3H, H10a&H10b, H8); ^**13**^**C{**^**1**^**H}-NMR (151 MHz, D**_**2**_**O)** δ 137.7 (C2), 117.7 (C1), 72.5 (C3), 71.7 (C4), 70.3 (C9), 69.4 (C8), 68.4 (C5/C6/C7), 68.3 (C5/C6/C7), 68.2 (C5/C6/C7), 63.3 (C10) ppm; **HRMS (ESI)***m*/*z* [M + H]^+^ calc. for C_10_H_21_O_8_: 269.1236, found: 269.1240.

#### 3,4,5,6,7,8,9,10-Octa-*O*-benzyl-1,2-dideoxy-l-*lyxo*-l-*manno*-dec-1-enitol (**22**)

Enitol **10** (1.20 g, 4.47 mmol, 1.00 equiv) was dissolved in dry DMF (35 mL) under an Ar atmosphere, and NaH (60% dispersion in paraffin oil, 3.97 g, 89.4 mmol, 20.0 equiv) was added portionwise to the stirring mixture under ice-bath cooling (formation of H_2_). The reaction mixture was allowed to warm up to rt and stirred until no further formation of H_2_ was observed (30 min). Then, benzyl bromide (8.68 mL, 12.5 g, 71.5 mmol, 16.0 equiv) was added dropwise again under ice-bath cooling, which led to the formation of a beige precipitate. After complete addition of BnBr, the cooling bath was removed and *n*-Bu_4_NI (0.84 g, 2.2 mmol, 0.50 equiv) was added and stirring was continued overnight. TLC (LP/EtOAc 3:1) indicated complete conversion, and excessive reagent was quenched by the addition of MeOH (20 mL). After dilution with Et_2_O (150 mL), extraction with H_2_O (3 × 50 mL) was performed and the combined aq. phase was extracted with Et_2_O (2 × 30 mL). The pooled organic phase was washed with sat. aq. NH_4_Cl and brine. After drying over anhydrous Na_2_SO_4_, the solvent was removed *in vacuo*. The residue was taken up in acetonitrile (150 mL), washed with *n*-hexane (2 × 50 mL), and vaporized again, giving a yellow oil (7.46 g). The crude product was purified via flash column chromatography (450 g SiO_2_, LP/EtOAc 10:1 → 4:1) to yield the desired product **22** as a colorless oil (3.75 g, 85%). ^**1**^**H NMR (400 MHz, CDCl**_**3**_**)** δ 7.33–7.17 (m, 40H, 40 × PhCH), 5.90 (ddd, *J* = 16.5, 11.0, 7.9 Hz, 1H, H2), 5.35–5.27 (m, 2H, H1a&H1b), 4.75–4.50 (m, 13H, 6 × CH_2_–Ph, C***H***H-Ph), 4.44–4.35 (m, 2H, CH_2_–Ph), 4.23–4.15 (m, 4H, 3 × CH, C***H***H-Ph), 4.12–4.02 (m, 3H, H3, 2 × CH), 3.96 (dt, *J* = 6.8, 1.5 Hz, 1H, H4), 3.73–3.63 (m, H10a&H10b); ^**13**^**C{**^**1**^**H}-NMR (151 MHz, CDCl**_**3**_**)** δ 139.3, 139.2, 139.15, 139.14, 139.09, 138.9, 138.7, 138.4 (8 × PhC1), 136.4 (C2), 128.5–127.3 (40 × PhCH), 119.7 (C1), 81.2 (C4), 81.1 (C3), 78.7, 78.4, 78.3, 77.8, 77.6 (5 × CH), 73.8 73.4, 73.3, 73.2, 72.9, 72.8, 72.4 (7 × ***C***H_2_–Ph), 70.8 (C10), 70.1 (***C***H_2_–Ph) ppm; **LC-MS (DUIS)***m*/z [M + H]^+^ calc. for C_66_H_69_O_8_: 989.50, found: 989.65; *m*/z [M + NH_4_]^+^ calc. for C_66_H_72_NO_8_: 1006.53, found: 1006.70. With the available HRMS equipment, no ionization of compound **22** could be achieved.

#### 3,4,5,6,7,8,9,10-Octa-*O*-benzyl-l-*galacto*-l-*talo*-decitol (***epi*****-23**)

AD-mix-β (×3) (2.14 g, 1.40 g/mmol starting material) (for preparation, see the SI) was suspended in *t*-BuOH/H_2_O (1:1, 3.0 mL), and MsNH_2_ (295 mg, 3.04 mmol, 2.00 equiv) was added under vigorous stirring. After 30 min, the protected octenitol **22** (1.50 g, 1.52 mmol, 1.00 equiv) was added as a solution in DCM (1.5 mL). After 2 days, TLC (LP/EtOAc 3:1) indicated complete conversion and the reaction mixture was quenched by the addition of Na_2_S_2_O_5_, until further addition of Na_2_S_2_O_5_ no longer resulted in foaming. After dilution with H_2_O (40 mL) and DCM (60 mL), the phases were separated, and the aq. phase was extracted with DCM (3 × 50 mL). The pooled organic phase was washed with brine (10 mL), dried over Na_2_SO_4_, and vaporized, giving the crude material (1.70 g) as a yellow oil (*talo*-isomer as a major product). This was purified via column chromatography (170 g SiO_2_, LP/EtOAc 95:5 → 70:30 (slow gradient)), giving compound ***epi*****-23** (1.39 g, 89%) as a colorless solid. **mp** 66–67 °C (EtOAc); ^**1**^**H NMR (600 MHz, CDCl**_**3**_**)** δ 7.31–7.19 (m, 38H, 38 × PhCH), 7.17–7.14 (m, 2H, 2 × PhCH), 4.74–4.64 (m, 5H, CH_2_–Ph), 4.61–4.52 (m, 7H, C***H***H-Ph, 3 × CH_2_–Ph), 4.50 (d, *J* = 11.4 Hz, 1H, C***H***H-Ph), 4.45–4.37 (m, 3H, C***H***H-Ph, CH_2_–Ph), 4.19–4.14 (m, 2H, 2 × CH), 4.10–4.05 (m, 2H, 2 × CH), 4.04–4.01 (m, 2H, 2 × CH), 3.80–3.67 (m, 4H, 2 × CH, H10a&H10b), 3.63 (dd, *J* = 11.4, 3.4 Hz, 1H, H1a), 3.53 (dd, *J* = 11.4, 4.6 Hz, 1H, H1b); ^**13**^**C{**^**1**^**H}-NMR (151 MHz, CDCl**_**3**_**)** δ 138.93, 138.88, 138.7, 138.6, 138.43, 138.41, 138.3, 138.2 (8 × PhC1), 128.5–127.4 (40 × PhCH), 80.9, 80.0, 78.7, 78.3, 78.21, 78.16, 77.5 (7 × CH), 74.4, 73.6, 73.54, 73.46, 73.4, 72.9, 72.79, 72.77 (8 × ***C***H_2_–Ph), 71.3 (C2), 70.6 (C10), 63.9 (C1) ppm; **HRMS (ESI)***m*/z [M + H]^+^ calc. for C_66_H_71_O_10_: 1023.5047, found: 1023.5077.

#### 3,4,5,6,7,8,9,10-Octa-*O*-benzyl-1,2-di-*O*-(4-nitrobenzoyl)-l-*galacto*-l-*galacto*-decitol (**24**)

PPh_3_ (641 mg, 2.45 mmol, 5.00 equiv) was weighed in a flame-dried Schlenk flask, and Schlenk technique was applied. Dry THF (3 mL) was added, the mixture was cooled to 0 °C using an ice-bath, and DIAD (526 μL, 2.45 mmol, 5.00 equiv) was added dropwise, which led to the formation of a beige precipitate. Additional dry THF (1 mL) was added to guarantee efficient stirring. After 30 min, the starting material **23** (500 mg, 0.489 mmol, 1.00 equiv., predried by azeotropic evaporation with dry toluene (3 × 2 mL)) was added to the reaction mixture as a solution in THF (2 mL). Then, 4-nitrobenzoic acid (409 mg, 2.45 mmol, 5.00 equiv) was added and the reaction mixture was allowed to slowly warm up to rt and further heated to 45 °C. TLC analysis (LP/EtOAc) after 18 h indicated complete conversion of the starting material. The solvent was evaporated, and the residue was taken up in cold Et_2_O (5 mL). Upon stirring under ice-bath cooling, a white precipitate (PPh_3_=O) was formed that was removed by filtration over a pad of celite. The filter pad was washed with cold Et_2_O, and the filtrate was diluted with further Et_2_O. The org. phase (100 mL in total) was washed with sat. aq. NaHCO_3_ (2 × 40 mL) and brine (10 mL). After drying over Na_2_SO_4_, the solvent was removed *in vacuo*, giving a yellow oil as a crude material (1.92 g). This was purified via flash column chromatography (hexane/EtOAc 25:1 → 5:1), giving the targeted product **24** (320 mg, 51%) as a slightly yellow oil. ^**1**^**H NMR (600 MHz, CDCl**_**3**_**)** δ 8.19–8.14 (m, 2H, 2 × PNBCH), 8.06–7.97 (m, 6H, 6 × PNBCH), 7.32–7.06 (m, 40H, 40 × PhCH), 6.09–5.96 (m, 1H, H2), 4.87 (d, *J* = 11.6 Hz, 1H, C***H***H-Ph), 4.79 (d, *J* = 11.1 Hz, 1H, C***H***H-Ph), 4.75 (d, *J* = 11.7 Hz, 1H, C***H***H-Ph), 4.74–4.48 (m, 10H, 5 × C***H***H-Ph, 2 × CH_2_–Ph, H1a), 4.44 (d, *J* = 5.6 Hz, 2H, CH_2_–Ph), 4.42–4.34 (m, 4H, 2 × CH, 2 × C***H***H-Ph), 4.27–4.22 (m, 1H, CH), 4.11 (dd, *J* = 5.8, 3.8 Hz, 1H, CH), 4.08–4.02 (m, 4H, H9, H1b, 2 × CH), 3.81 (dd, *J* = 10.2, 6.1 Hz, 1H, H10a), 3.77 (dd, *J* = 10.2, 4.8 Hz, 1H, H10b); ^**13**^**C{**^**1**^**H}-NMR (151 MHz, CDCl**_**3**_**)** δ 164.2, 164.1 (2 × C=O), 150.6, 150.5 (2 × PNBC4), 139.2, 138.8, 138.70, 138.67, 138.6, 138.3, 137.9, 137.5 (8 × PhC1), 135.3, 135.1 (2 × PNBC1), 130.85, 130.80 (2 × PNBCH), 128.7–127.3 (40 × PhCH), 123.64, 123.63 (2 × PNBCH), 79.1, 78.9, 78.8, 78.7, 77.4*, 76.9, 76.5 (7 × CH), 74.6, 74.4, 73.4, 73.3, 73.2, 72.9 (7 × ***C***H_2_–Ph), 72.1 (C2), 71.5 (***C***H_2_–Ph), 70.9 (C10), 65.4 (C1) ppm; **LC-MS (DUIS)***m*/z [M + NH_4_]^+^ calc. for C_80_H_80_N_3_O_16_: 1338.5539, found: 1338.85. With the available HRMS equipment, no ionization of compound **24** could be achieved. *Shift determined from HSQC due to overlap with the residual solvent signal.

#### 3,4,5,6,7,8,9,10-Octa-*O*-benzyl-l-*galacto*-l-*galacto*-decitol (**23**)

The Mitsunobu product **24** (0.30 g, 0.23 mmol, 1.00 equiv) was dissolved in THF (4.5 mL), and aq. LiOH (0.5 M, 2.3 mL, 1.1 mmol, 5.0 equiv) was added under ice-bath cooling. The reaction mixture was stirred at rt overnight when TLC analysis (LP/EtOAc 3:1) indicated complete conversion. The solution was concentrated and taken up in H_2_O (50 mL) and DCM (50 mL). After separation of the phases, the aq. phase was extracted with DCM (3 × 30 mL) and the pooled organic phase was washed with sat. aq. NaHCO_3_ (30 mL) and brine. After drying over Na_2_SO_4_, the solvent was removed *in vacuo*, giving 0.26 g of crude material. This was purified via column chromatography (26 g SiO_2_, hexane/EtOAc 8:1 → 2:1), giving the targeted product **23** (166 mg, 72%) as a colorless solid. **mp** 84–86 °C (hexane/EtOAc); ^**1**^**H NMR (400 MHz, CDCl**_**3**_**)** δ 7.35–7.20 (m, 38H, 38 × PhCH), 7.17 (dd, *J* = 7.7, 1.7 Hz, 2H, 2 × PhCH), 4.86–4.52 (m, 13H, 6 × CH_2_–Ph, C***H***H-Ph), 4.46 (d, *J* = 2.6 Hz, 2H, CH_2_–Ph), 4.36–4.31 (m, 2H, C***H***H-Ph, H6), 4.27–4.20 (m, 2H, H5, H7), 4.15 (dd, *J* = 5.6, 4.3 Hz, 1H, H4), 4.13–4.09 (m, 2H, H9), 4.07 (dd, *J* = 7.3, 3.2 Hz, 1H, H8), 3.92 (t, *J* = 4.6 Hz, 1H, H2), 3.75–3.70 (m, 3H, H10a&H10b, H3), 3.59 (dd, *J* = 11.1, 6.7 Hz, 1H, H1a), 3.45 (dd, *J* = 11.1, 4.9 Hz, 1H, H1b); ^**13**^**C NMR (101 MHz, CDCl**_**3**_**)** δ 139.0, 138.9, 138.8 (2 × ), 138.6, 138.32, 138.26, 137.9 (8 × PhC1), 128.5–127.4 (40 × PhCH), 79.9 (C4), 78.9 (C8), 78.3 (C7), 78.20 (C9), 78.17 (C3), 77.1 (C6), 76.8 (C5), 74.9, 73.79, 73.76, 73.4, 73.3, 72.7, 72.1, 71.9 (8 × ***C***H_2_–Ph), 71.3 (C2), 70.6 (C10), 64.3 (C1) ppm; **HRMS (ESI)***m*/z [M + H]^+^ calc. for C_66_H_71_O_10_: 1023.5047, found: 1023.5079.

#### meso-l-Galacto-l-galacto-decitol (**6**)

The protected *galacto*-decitol **23** (50.6 mg, 49.4 μmol, 1.00 equiv) was dissolved in a mixture of EtOAc/MeOH (4:1, 5 mL), and a drop of acetic acid was added. Pd/C (10 wt %, 10.5 mg, 20 mol %) was added under an Ar atmosphere, and the vial was placed in a steel autoclave. The atmosphere was changed to H_2_ (50 bar), and the reaction mixture was stirred at rt for 7 days. LC-MS analysis indicated complete conversion to the desired product. The mixture was filtered over celite (H_2_O washed), and the filter pad was washed with hot H_2_O. The filtrate was concentrated *in vacuo* and lyophilized, giving an off-white solid. This was triturated with EtOH (5 mL) and washed with H_2_O (2 × 1 mL). After drying *in vacuo* at 50 °C, product **6** was obtained as a colorless solid (14 mg, 94%), pure according to ^1^H NMR. For STA-measurements, small samples (∼10–15 mg) were recrystallized from H_2_O. **mp** > 290 °C (decomposition) (H_2_O) (lit.^21^ > 280 °C (decomposition) (H_2_O)); ^**1**^**H NMR (600 MHz, D**_**2**_**O)** δ 4.00–3.97 (m, 2H), 3.96–3.93 (m, 4H), 3.71–3.66 (m, 6H); ^**13**^**C{**^**1**^**H}-NMR (151 MHz, D**_**2**_**O)** δ 70.3*, 69.4*, 68.2*, 63.3* ppm; **HRMS (ESI)***m*/z [M – H]^−^ calc. for C_10_H_21_O_10_: 301.1135, found: 301.1141. *Shifts were determined from the HSQC spectrum since very long measurement times for a ^13^C NMR spectrum with satisfying S/N-ratio would have been necessary because of the low solubility of compound **6** even in D_2_O.

#### l-*Galacto*-l-*talo*-decitol (***epi*****-6**)

The protected decitol ***epi*****-23** (102 mg, 0.100 mmol, 1.00 equiv) was dissolved in EtOAc/MeOH (4:1, 10 mL), and a drop of acetic acid was added. Pd/C (10 wt %, 20 mg, 19 mol %) was added under an Ar atmosphere, and the vial was placed in a steel autoclave. The atmosphere was changed to H_2_ (50 bar), and the reaction mixture was stirred at rt for 4 days. LC-MS analysis indicated complete conversion to the desired product. The mixture was filtered over celite (H_2_O washed), and the filter pad was washed with hot H_2_O. The filtrate was concentrated *in vacuo* and lyophilized, giving an off-white solid. This was triturated with EtOH (5 mL) and washed with H_2_O (2 × 1 mL). After drying *in vacuo* at 50 °C, product ***epi*****-6** was obtained as a colorless solid (23 mg, 78%), pure according to ^1^H NMR. For STA-measurements, small samples (∼10–15 mg) were recrystallized from H_2_O. **mp** 242–243 °C (H_2_O) (decomposition >260 °C) (lit.^23^ 246–247.5 °C (EtOH/H_2_O)); ^**1**^**H NMR (600 MHz, D**_**2**_**O)** δ 3.98 (td, *J* = 6.5, 6.1, 1.4 Hz, 1H), 3.96–3.91 (m, 5H), 3.84–3.80 (m, 2H), 3.71–3.66 (m, 4H); ^**13**^**C{**^**1**^**H}-NMR (151 MHz, D**_**2**_**O)** δ 72.8, 71.6, 70.3, 69.6, 69.3, 68.5, 68.2, 68.0, 63.3, 61.9 ppm; **HRMS (ESI)***m*/z [M – H]^−^ calc. for C_10_H_21_O_10_: 301.1135, found: 301.1153.

### Toward the Nonitol

#### 3,4,5,6,7,8,9,10-Octa-*O*-acetyl-1,2-dideoxy-l-*lyxo*-l-*manno*-dec-1-enitol (**25**)

Decenitol **10** (0.10 mg, 0.38 mmol, 1.0 equiv) was taken up in pyridine (1.1 mL), and acetic anhydride (0.88 mL, 9.2 mmol, 24 equiv) was added dropwise under ice-bath cooling. After 10 min, a spatula of DMAP was added and the reaction mixture was stirred at rt for 2 h when TLC (LP/EtOAc 2:1) indicated full conversion of the starting material to a single spot. Excessive reagent was quenched by the addition of MeOH (0.5 mL), and the reaction mixture was diluted with DCM (20 mL). The org. phase was extracted with ice-cold 2 N HCl (2 × 10 mL, until aq. phase remained acidic), and the combined HCl-phase was extracted with DCM (10 mL). The pooled organic phase was washed with H_2_O (10 mL), sat. aq. NaHCO_3_ (10 mL), and brine (10 mL). After drying over Na_2_SO_4_, the solvent was evaporated *in vacuo*, giving a yellow, oily crude product (0.25 g). Pure product **25** (214 mg, 92%) was obtained via flash column chromatography (12 g SiO_2_, LP/EtOAc 3:1) as a colorless solid. **mp** 140–141.5 °C (EtOAc); ^**1**^**H NMR (400 MHz, CDCl**_**3**_**)** δ 5.70 (ddd, *J* = 17.2, 10.3, 7.6 Hz, 1H, H2), 5.39–5.24 (m, 5H, 2 × CH, H3, H1), 5.15 (dd, *J* = 9.0, 2.5 Hz, 1H, CH), 5.13–5.07 (m, 2H, H9, CH), 5.05 (dd, *J* = 7.3, 1.7 Hz, 1H, CH), 4.25 (dd, *J* = 11.8, 4.9 Hz, 1H, H10a), 3.84 (dd, *J* = 11.8, 6.7 Hz, 1H, H10b), 2.08, 2.06, 2.05, 2.04, 2.02, 2.02 (8 × s, 24H, 8 × COC***H***_3_). ^**13**^**C{**^**1**^**H}-NMR (101 MHz, CDCl**_**3**_**)** δ 170.6, 170.3, 170.2, 170.11, 170.0, 169.9, 169.8, 169.7 (8 × ***C***OCH_3_), 132.2 (C2), 121.2 (C1), 72.8, 69.7, 68.2, 68.1, 67.0, 66.9, 66.7 (7 × CH), 62.4 (C10), 21.2, 21.1, 21.0 (2 × ), 20.9, 20.83, 20.82, 20.79 (8 × CO***C***H_3_) ppm; **HRMS (ESI)***m*/z [M + Na]^+^ calc. for C_26_H_36_NaO_16_: 627.1901, found: 627.1907.

#### 1,2,3,4,5,6,7,8,9-Nona-*O*-acetyl-l-*lyxo*-l-*manno*-nonitol (**26**)

According to the literature,^28^ the decenitol peracetate **26** (478 mg, 0.790 mmol, 1.00 equiv) was dissolved in a DCM/MeOH mixture (3:1, 30 mL) and the solution was cooled to −78 °C using a liquid N_2_/acetone bath. Ozone was bubbled through until the solution turned dark blue. The ozone generator was turned off, and TLC (LP/EtOAc 3:1) was carried out after 30 min of stirring at −78 °C, showing complete conversion of the starting material. Then, NaBH_4_ (92 mg, 2.4 mmol, 3.0 equiv) was added and the reaction mixture was allowed to warm up to rt. Stirring was continued overnight until TLC indicated complete conversion. Acetic acid (5 mL) was added dropwise, and the solvent was evaporated subsequently. The residue was taken up in pyridine (1.7 mL), and acetic anhydride (0.23 mL, 2.4 mmol, 3.0 equiv) was added dropwise under ice-bath cooling. After 15 min, a spatula tip of DMAP was added and the reaction mixture was further stirred at rt. After 2 h, LC-MS indicated complete conversion to the desired product and excessive reagent was quenched by the addition of MeOH (0.2 mL). After dilution with EtOAc (20 mL), extraction with 1 N HCl (3 × 10 mL—until aq. phase remained acidic) was performed. The pooled organic phase was washed with sat. aq. NaHCO_3_ (5 mL) and brine (5 mL). After drying over Na_2_SO_4_, the solvent was removed *in vacuo*, giving a brown oil. This was purified via flash column chromatography (90 g SiO_2_, LP/EtOAc 4:1 → 1:1), and product **26** was obtained as a colorless solid (406 mg, 79%). **mp** 147.5–149 °C (EtOAc) (lit.^41^ 143–145 °C (for enantiomer)); ^**1**^**H NMR (400 MHz, CDCl**_**3**_**)** δ 5.35–5.29 (m, 3H, 3 × CH), 5.19–5.14 (m, 2H, 2 × CH), 5.11 (ddd, *J* = 7.3, 4.9, 2.5 Hz, 1H, CH), 5.00 (ddd, *J* = 7.7, 5.8, 3.2 Hz, 1H, CH), 4.27–4.22 (m, 2H, H1a, H9a), 4.00 (dd, *J* = 12.4, 5.8 Hz, 1H, H1b/H9b), 3.85 (dd, *J* = 11.8, 6.7 Hz, 1H, H1b/H9b), 2.09, 2.08, 2.07, 2.06, 2.06, 2.06, 2.03, 2.02 (9 × s, 27H, 9 × COC***H***_3_); ^**13**^**C{**^**1**^**H}-NMR (101 MHz, CDCl**_**3**_**)** δ 170.7, 170.6, 170.3, 170.1, 170.03, 169.99, 169.96, 169.88, 169.87 (9 × ***C***OCH_3_), 68.8, 68.2, 68.1, 67.9, 67.3, 67.0, 66.8 (7 × CH), 62.4 (C1/C9), 62.0 (C1/C9), 21.0, 20.94, 20.91, 20.87 (2 × ), 20.84, 20.82 (2 × ), 20.79 (9 × CO***C***H_3_) ppm; **HRMS (ESI)***m*/z [M + Na]^+^ calc. for C_27_H_38_NaO_18_: 673.1956, found: 673.1969.

#### l-*Lyxo*-l-*manno*-nonitol (**5**)

The nonitol peracetate **26** (397 mg, 0.610 mmol, 1.00 equiv) was taken up in MeOH (HPLC grade, 8 mL), and NaOMe (30% in MeOH) was added dropwise until pH was about 10. After 2 h, TLC analysis (LP/EtOAc 2:1) indicated complete conversion to a very polar spot. The reaction mixture was diluted with water, neutralized by the addition of Dowex-H^+^ resin (MeOH washed), and filtered. The filtrate was lyophilized giving product **6** as a colorless solid (146 mg, 88%), pure according to ^1^H NMR. For STA-measurements, small samples (∼10–15 mg) were recrystallized from MeOH/H_2_O (4:1). **mp** 249–252 °C (H_2_O) (lit.^41^ 250–255 °C (for enantiomer)); ^**1**^**H NMR (600 MHz, D**_**2**_**O)** δ 3.98 (ddd, *J* = 7.3, 6.0, 1.5 Hz, 1H, CH), 3.94 (d, *J* = 9.6 Hz, 1H, CH), 3.93–3.91 (m, 2H, 2 × CH), 3.86 (dd, *J* = 11.8, 2.9 Hz, 1H, H1a/H9a), 3.82 (d, *J* = 9.0 Hz, 1H, CH), 3.76 (ddd, *J* = 8.9, 6.3, 2.8 Hz, 1H, CH), 3.70–3.65 (m, 4H, CH, H1a/H9a, H1b, H9b); ^**13**^**C{**^**1**^**H}-NMR (151 MHz, CDCl**_**3**_**)** δ 70.9, 70.3, 69.3, 69.2, 68.3, 68.2, 68.0, 63.3 (C1/C9), 63.2 (C1/C9) ppm; **HRMS (ESI)***m*/z [M – H]^−^ calc. for C_9_H_19_O_9_: 271.1029, found: 271.1040 (1.95 ppm); **HRMS (ESI)***m*/z [M + Na]^+^ calc. for C_9_H_20_NaO_9_: 295.1005, found: 295.1009.

## Data Availability

The data underlying this study are available in the published article and its Supporting Information.

## Supporting Information Available

Additional experimental details; compound characterization data; curves of STA and DSC measurements; and ^1^H NMR and ^13^C{^1^H} NMR spectra (PDF)
