# Supplementary material for: Indium-Mediated Acyloxyallylation-Based Synthesis of Galacto-Configured Higher-Carbon Sugar Alcohols as Potential Phase Change Materials
Source: J Org Chem. 2024 Apr 5;89(8):5573–88. doi: 10.1021/acs.joc.4c00067 (PMC11040715; doi:10.1021/acs.joc.4c00067)
Supplement: Supplementary file 1 — jo4c00067_si_001.pdf [file jo4c00067_si_001.pdf]

Supporting Information

Indium-Mediated Acyloxyallylation-Based Synthesis of *Galacto*-Configured Higher-Carbon Sugar Alcohols as Potential Phase Change Materials

Nina Biedermann†, Julian Schnizer†, Daniel Lager‡, Michael Schnürch*,†, Christian Stanetty*,†

† Institute of Applied Synthetic Chemistry, TU Wien, Getreidemarkt 9/163, 1060 Vienna, Austria

‡ Energy Department, AIT Austrian Institute of Technology GmbH, Giefinggasse 2, 1210 Vienna, Austria

E-Mail: michael.schnuerch@tuwien.ac.at, christian.stanetty@tuwien.ac.at

Table of content

General information.......... S2

Synthesis of the elongation reagents used in the IMA S4

3-Bromoprop-1-en-1-yl acetate (**9**) (Mixture of (*E*)- and (*Z*)-isomer) S4

3-Bromoprop-1-en-1-yl benzoate (**20**) (Mixture of (*E*)- and (*Z*)-isomer) S4

3-Bromoprop-1-en-1-yl pivalate (**21**) (Mixture of (*E*)- and (*Z*)-isomer).......... S5

Side product in the synthesis of the acetonide-protected octenitol **14**.......... S6

Screening of dihydroxylation conditions and octenitol substrates S7

General procedure for the Upjohn dihydroxylation conditions.......... S7

General procedure for the Sharpless dihydroxylation conditions.......... S7

General procedure for the deacetylation of octitol mixture **15** / ***epi*-15**.......... S8

General procedure for the catalytic hydrogenation of octitol mixture **16** / ***epi*-16** S8

General procedure for the cleavage of acetonide protecting groups of crude octitol mixture **17** / ***epi*-17** .. S8

Analysis of crude entiol mixture toward decenitol **10**.......... S9

Attempted synthesis toward the L-*glycero*-D-*manno*-D-*manno*-dodecenitol **SI-3**.......... S10

L-*Lyxo*-L-*manno*-nonose (**SI-1**) S11

1,2,3,4,6,7,8,9-Octa-*O*-acetyl-L-*lyxo*-L-*manno*-nonose (**SI-2**) S12

1,2-Dideoxy-L-*glycero*-D-*manno*-D-*manno*-dodecenitol (**SI-3**) S12

The open-chain contents of heptose **11** and nonose **SI-1** S14

General procedure for the ABAO assay used for the determination of the OCCs S14

STA / DSC-measurements S15

Spectra S19

References S44

General information

The used reagents and solvents were purchased from commercial sources with a purity of >95%, unless noted different, and used without further purification. Water-free solvents were available from a PureSolv solvent purification system by Innovative Technology, or commercial sources that were stated as water-free and stored in bottles with septum and over molecular sieve. Dowex H^+ was washed with respective solvent before use.

Thin-layer chromatography (TLC) for reaction monitoring and fraction analysis from column chromatography was performed with silica gel 60 F_{254} plates or HPTLC-plates (silica gel 60 F_{254} with concentration zone 20×2.5 cm). Visualization of the spots was done using UV light (254 nm) or via heat staining the plates with anisaldehyde solution (180 mL EtOH, 10 mL anisaldehyde, 10 mL H_2SO_4 (conc.), 2 mL AcOH), permanganate solution (3.0 g $KMnO_4$, 20.0 g K_2CO_3, 250 mg KOH, 300 mL H_2O) or cerium molybdate ("Mostain", 21.0 g $(NH_4)_6Mo_7O_{24}\cdot 2H_2O$, 1.0 g $Ce(SO_4)$, 31 mL H_2SO_4 (conc.), 500 mL H_2O).

For flash column chromatography, columns were packed with silica gel from Merck with a pore size of 40-63 µm. Purification was either done by hand column or on a Büchi Pure C-850 FlashPrep System. Light petroleum is referred to as LP.

LC-MS analysis was performed on a Nexera X2® UHPLC system (Shimadzu®, Kyoto, Japan) comprised of LC-30AD pumps, a SIL-30AC autosampler, CTO-20AC column oven, DGU-20A5/3 degasser module. Detection was accomplished by an SPD-M20A photo diode array and a LCMS-2020 mass spectrometer. Separations were either performed using a Waters® XSelect® CSH™ C18 2.5 µm (3.0×50 mm) Column XP at 40 °C, a flowrate of 1.7 mL/min and with UHPLC grade water and acetonitrile containing 0.1% formic acid as the mobile phase, or a Waters® XBridge® BEH Amide 2.5 µm (3.0×50 mm) Column XP at 40 °C, a flowrate of 1.3 mL/min and with UPLC grade water (pH 8.5, 2.5 mM NH_4COOH) and acetonitrile.

Accurate mass analysis was performed on an Agilent 6230 AJS ESI-TOF mass spectrometer with ESI ionisation method or Q Exactive Focus, ESI, FIA injection, mobile phase 18% MeCN with 0.1% formic acid.

^{1}H-NMR and ^{13}C-NMR spectra were recored at ambient temperature in the solvent indicated using a Bruker Avance Ultra Shield 400 MHz and an Avance III HD 600 MHz spectrometer with TMS as internal standard. Processing of the data was performed with standard software and all spectra were calibrated to the solvent residual peak. Chemical shifts (δ) are reported in ppm, coupling constants (*J*) in hertz (Hz) and multiplicities are assigned as s = singlet, d = doublet, t = triplet, q = quartet, m = multiplet etc. All assignments are based on 2D-sepctra (COSY, phase sensitive HSQC, HMBC – depending on the molecule).

Melting points were recorded using a BÜCHI Melting Point B 545 with a 40%/90% threshold and a heating rate of 1.0 °C/min or a Kofler-type Leica Galen III micro hot stage microscope.

Ozone enriched oxygen was generated using a Triogen LAB2B Ozone generator.

Simultaneous thermal analysis (STA) including differential scanning calorimetry (DSC) and thermogravimetric analysis (TG) measurements were performed on a Netzsch STA 449 F1 *Jupiter* under nitrogen atmosphere with a constant gas flow rate of 40 mL/min and a heating and cooling rate of 10 °C/min if not stated otherwise. Samples were measured using Al pans (25 µL) with a hole in the lid. Samples were heated to approximately 30-40 °C above their melting points to prevent any decomposition at higher

temperature. DSC-measurements were performed on a Netzsch DSC 204 F1 *Phoenix* with the same measuring parameters but using closed Al pans (25 µL).

Compounds were named according to IUPAC systematic standards, in general. When it comes to higher-carbon sugar species (more than six carbon atoms), names were generated by dividing the sugar species into groups of up to four chiral centers consequently starting from the chiral center next to the former reducing end (on the right for all displayed structures). To these groups, configurational prefixes were assigned, and the name was built up by putting the prefix of the group that is farthest from the right end (C1) first. This group may contain less than four carbon atoms. Numbering of compounds was performed in the same way, always starting with 1 at the former reducing end as shown in the exemplary structures below.

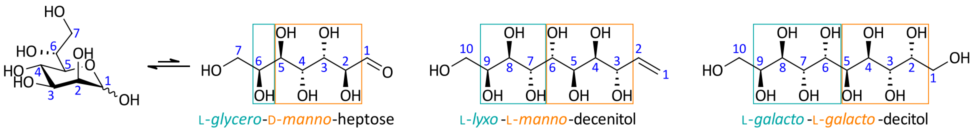

Synthesis of the elongation reagents used in the IMA

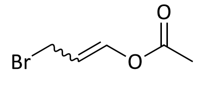
9

3-Bromoprop-1-en-1-yl acetate (9) (Mixture of (*E*)- and (*Z*)-isomer)

According to a literature protocol[1], acrolein (90%, 4.7 g, 5.6 mL, 84 mmol, 1.0 equiv) was dissolved in dry DCM (65 mL), cooled to −20 °C using an acetone/liquid N2 cooling bath. Acetyl bromide (9.8 g, 6.0 mL, 80 mmol, 0.95 equiv) was added to the solution under stirring followed by anhydrous $ZnCl_2$ (0.11 g, 0.84 mmol, 1.0 mol%). The reaction mixture was allowed to warm up to −15 °C by lowering the cooling bath as the exothermic reaction started and a temperature jump to +12 °C could be observed. The flask was then re-immersed in the cooling bath, and the reaction mixture was stirred for 1 hour, keeping the temperature below −10 °C. As ^{1}H-NMR analysis (micro work-up with Et_2O and aq. sat. $NaHCO_3$, drying over Na_2SO_4) confirmed full conversion of the starting material to the targeted product, H_2O (30 mL) was added under cooling which led to the formation of a white precipitate and a temperature rise to −10 °C. Layers were separated and the organic layer was washed with water (30 mL) followed by sat. aq. $NaHCO_3$ (2×40 mL – until pH remained basic). The greenish organic phase was further washed with brine, dried over anhydrous Na_2SO_4 and evaporated, giving a brown, oily liquid. The pure product **9** was obtained by distillation *in vacuo* (**bp** 85-92 °C, 26-30 mbar) as a colorless to slightly yellow liquid (9.9 g, 73%) in a ratio of E/Z = ~1:2.6 (according to ^{1}H-NMR). Spectral data is in accordance with literature[1].

(*E*)-isomer: **^{1}H-NMR (400 MHz, $CDCl_3$)** δ 7.42 (dt, *J* = 12.4, 1.1 Hz, 1H, =CH-O), 5.70 (dt, *J* = 12.4, 8.4 Hz, 1H, CH_2-C***H***=), 3.97 (d, *J* = 8.4 Hz, 2H, CH_2-Br), 2.15 (s, 3H, CH_3); **^{13}C{^{1}H}-NMR (101 MHz, $CDCl_3$)** δ 167.7 (C=O), 139.3 (=CH-O), 111.4 (CH_2-***C***H=), 28.6 (CH_2-Br), 20.7 (CH_3) ppm.
(*Z*)-isomer: **^{1}H-NMR (200 MHz, $CDCl_3$)** δ 7.18 (dt, *J* = 6.3, 0.9 Hz, 1H, =CH-O), 5.23 (td, *J* = 8.4, 6.3 Hz, 1H, CH_2-C***H***=), 4.08 (dd, *J* = 8.4, 0.9 Hz, 2H, CH_2-Br), 2.20 (s, 3H, CH_3); **^{13}C{^{1}H}-NMR (101 MHz, $CDCl_3$)** δ 167.3 (C=O), 137.2 (=CH-O), 109.6 (CH_2-***C***H=), 23.7 (CH_2-Br), 20.8 (CH_3) ppm.

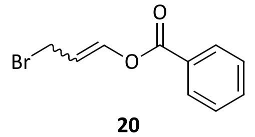
20

3-Bromoprop-1-en-1-yl benzoate (20) (Mixture of (*E*)- and (*Z*)-isomer)

According to a literature protocol[2-3], acrolein (90%, 1.50 g, 1.79 mL, 26.8 mmol, 1.00 eq.) was dissolved in dry DCM (60 mL), cooled to −20 °C using an acetone/liquid N_2 cooling bath. Then, benzoyl bromide (4.70 g, 25.4 mmol, 0.95 equiv) was added to the solution under stirring followed by anhydrous $ZnCl_2$ (36 mg, 0.27 mmol, 1.0 mol%). The reaction mixture was allowed to warm up to −15 °C by lowering the cooling bath, and stirring was continued for one hour, keeping the temperature below −10 °C. As ^{1}H-NMR-analysis (micro work-up with Et_2O and aq. sat. $NaHCO_3$, drying over Na_2SO_4) did not show any formed product but just starting material the stirring mixture was allowed to warm up to 0 °C and kept as this temperature for 2 hours. Within this time, the solution turned orange and ^{1}H-NMR-analysis confirmed full conversion of the starting material to the targeted product. Aq. sat. $NaHCO_3$ (40 mL) was slowly added to the mixture, followed by further DCM (200 mL). The layers were separated, and the aqueous phase was extracted with another portion of DCM (150 mL). The combined organic phase was dried over anhydrous Na_2SO_4 and evaporated giving a brown, oily liquid (5.5 g). The pure product **20** was

obtained by flash column chromatography (100 g SiO_2, LP/EtOAc 9:1, fraction size 150 mL) as a colorless liquid (4.2 g, 72%) in a ratio of *E*/*Z* = ~0.4:0.6 (according to ^{1}H-NMR) that solidified in the freezer (−18 °C) giving colorless needles. Spectral data is in accordance with literature[3].

mp 68.8-70.5 °C (EtOAc) (lit.[2] 74-76 °C (pentane)); **^{1}H-NMR (400 MHz, $CDCl_3$)** δ 8.15 – 8.08 (m, 2H, C***H*** Ph (E/Z)), 7.68 (dt, *J* = 12.4, 1.0 Hz, 0.6H, =CH-O (*Z*)), 7.65 – 7.59 (m, 1H, C***H*** Ph *(E*/*Z*)), 7.54 – 7.46 (m, 2H, C***H*** PH (*E*/*Z*)), 7.44 (dt, *J* = 6.2, 0.7 Hz, 0.4H, =CH-O (*E*)), 5.90 (dt, *J* = 12.3, 8.4 Hz, 0.4H, =CH-C (*E*)), 5.39 (td, *J* = 8.4, 6.2 Hz, 0.6H, =CH-C (*Z*)), 4.24 – 4.20 (m, 1.2H, CH_2-Br (*Z*)), 4.07 (dd, *J* = 8.4, 1.0 Hz, 0.8H, CH_2-Br (*E*)); **^{13}C{^{1}H}-NMR (101 MHz, $CDCl_3$)** δ 163.3 (C=O (*Z*)), 162.9 3 (C=O (*E*)), 139.6 (=CH-O (*Z*)), 137.7 (=CH-O (*E*)), 134.1, 134.0, 130.3, 130.2, 128.9, 128.8 (2×***C***H Ph), 128.7, 128.6 (2×qC Ph), 112.0 (=***C***H-C (*Z*)), 110.2 (=***C***H-C (*E*)), 28.7 (CH_2-Br (*Z*)), 23.7 (CH_2-Br (*E*)) ppm.

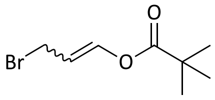

21

3-Bromoprop-1-en-1-yl pivalate (21) (Mixture of (*E*)- and (*Z*)-isomer)

According to a literature protocol[2-3]: <u>Step 1 – synthesis of pivaloyl bromide:</u> PPh_3 (89.9 g, 343 mmol, 1.00 equiv) was dissolved in dry DCM (170 mL) and cooled to −10 °C, using an ice/NaCl-mixture (3:1). Then, Br_2 (54.8 g, 17.6 mL, 343 mmol, 1.00 equiv) was added dropwise as a solution in dry DCM (170 mL) in a way that the temperature stayed below 0 °C. The formation of a white precipitate (PPh_3Br_2) was obtained. Further, a solution of pivalic acid (35.0 g, 343 mmol, 1.00 equiv) in dry DCM (170 mL) was prepared that was quickly added to the reaction mixture after complete addition of Br_2. The mixture turned into a clear, orange solution and was stirred at rt for 1.5 hours. Then, the solvent was removed, and the precipitate was taken up in dry Et_2O that led to the formation of more precipitate. The solid was removed via filtration, and the filtrate was concentrated *in vacuo*. Pivaolyl was obtained by distillation *in vacuo* as a colorless liquid (29.4 g, 52%). **bp** 27-29 °C, 10 mbar (lit.[4] 65 °C, 15 Torr); **^{1}H-NMR (400 MHz, $CDCl_3$)** δ 1.31 (s, 9H, 3×CH_3) ppm. <u>Step 2 – synthesis of 3-bromoprop-1-en-1-yl pivalate:</u> acrolein (90%, 11.1 g, 13.2 mL, 198 mmol, 1.00 equiv) was dissolved in dry DCM (100 mL), cooled to −20 °C using an acetone/liquid N_2 cooling bath. Pivaloyl bromide (29.3 g, 178 mmol, 0.90 equiv) was added to the solution under stirring followed by anhydrous $ZnCl_2$ (269 mg, 1.98 mmol, 0.01 equiv). The reaction mixture was allowed to warm up to −10 °C by lowering the cooling bath as the exothermic reaction started and a temperature jump to +10 °C could be observed. The flask was then re-immersed in the cooling bath, and the reaction mixture was stirred for one hour, keeping the temperature below −10 °C. As ^{1}H-NMR-analysis (micro work-up with Et_2O and aq. sat. $NaHCO_3$, drying over Na_2SO_4) confirmed full conversion of the starting material to the targeted product, H_2O (40 mL) was added under cooling which led to the formation of a white precipitate and the temperature rose to −10 °C. The layers were then separated, and the organic layer was washed with sat. aq. $NaHCO_3$ (2×30 mL – until pH remained basic). The greenish organic phase was further washed with brine, dried over anhydrous Na_2SO_4 and evaporated giving a brown, oily liquid. Pure product **21** was obtained by distillation *in vacuo* (**bp** 73 °C, 3.2 mbar; lit.[5] 38 °C, 0.2 mbar) as a colorless to slightly yellow liquid (25.6 g, 65%) in a ratio of *E*/*Z* = ~1:1.7 (according to ^{1}H-NMR). Spectral data is in accordance with literature[3].

(E)-isomer: **^{1}H-NMR (400 MHz, $CDCl_3$)** δ 7.42 (d, J = 12.4 Hz, 1H, =C***H***-O), 5.71 (dt, J = 12.4, 8.4 Hz, 1H, CH_2-C***H***=), 4.00 (dd, J = 8.4, 0.8 Hz, 2H, CH_2-Br), 1.24 (s, 9H, 3×CH_3); **^{13}C{^{1}H}-NMR (101 MHz, $CDCl_3$)** δ 175.2 (C=O), 139.7 (=CH-O), 111.1 (CH_2-***C***H=), 38.9 (***C***(CH_3)$_3$), 28.9 (CH_2–Br), 27.0 (3×C(***C***H_3)$_3$) ppm.

(Z-isomer): **^{1}H-NMR (400 MHz, $CDCl_3$)** δ 7.18 (d, J = 6.2 Hz, 1H, =C***H***-O), 5.26 (td, J = 8.4, 6.2 Hz, 1H, CH_2-C***H***=), 4.07 (d, J = 8.4 Hz, 2H, CH_2-Br), 1.29 (s, 9H, 3×CH_3); **^{13}C{^{1}H}-NMR (101 MHz, $CDCl_3$)** δ 174.7 (C=O), 137.9 (=CH-O), 109.6 (=CH_2-***C***H=), 39.2 (***C***(CH_3)$_3$), 27.1 (3×C(***C***H_3)$_3$), 23.6 (CH_2–Br) ppm.

Side product in the synthesis of the acetonide-protected octenitol 14

Additional to the targeted 3,4:5,6:7,8-O-triacetonide **14**, that was isolated in 19% yield, a main side product with similar polarity was separated via column chromatography. The structure of this side product could not be fully clarified by NMR analysis (^{1}H-NMR, ^{13}C-NMR, COSY, HSQC, HMBC). It could be confirmed that it is an octenitol species with three isopropylidene groups present, but the spectral data did not proved information on the very constitution. However, in the HBMC spectrum, a correlation between the H5 and H7 with the same quaternary carbon atom from an isopropylidene group was observed, an indication for the formation of a species with 6-membered instead of 5-membered rings. Since no further signals with information on the position of the isopropylidene groups were detected, no structure could be proposed.

^{1}H-NMR (400 MHz, $CDCl_3$) δ 6.04 (ddd, J = 17.9, 10.2, 8.1 Hz, 1H, H2), 5.37 (d, J = 17.4 Hz, 1H, H1a), 5.28 (d, J = 10.3 Hz, 1H, H1b), 4.66 (t, J = 7.5 Hz, 1H, H3), 4.21 (dd, J = 7.0, 1.6 Hz, 1H, H4), 4.18 (dd, J = 7.1, 3.6 Hz, 1H, H6), 3.99 (dd, J = 11.9, 5.1 Hz, 1H, H8a), 3.82 – 3.77 (m, 1H, H7), 3.73 (dd, J = 11.9, 5.0 Hz, 1H, H8b), 3.53 (dd, J = 7.0, 1.6 Hz, 1H, H5), 1.51 (s, 3H, CH_3), 1.42 (s, 3H, CH_3), 1.40 – 1.36 (m, 9H, 3×CH_3), 1.35 (s, 3H, CH_3); **^{13}C{^{1}H}-NMR (151 MHz, $CDCl_3$)** δ 134.0 (C2), 119.8 (C1), 109.3 (***C***(CH_3)$_2$), 101.0 (***C***(CH_3)$_2$), 99.0 (***C***(CH_3)$_2$), 79.4 (C3), 78.2 (C4), 70.3 (C5), 68.4 (C6), 64.2 (C7), 61.0 (C8), 27.2, 26.6, 26.0, 24.8, 24.4, 21.5 (6×CH_3) ppm.

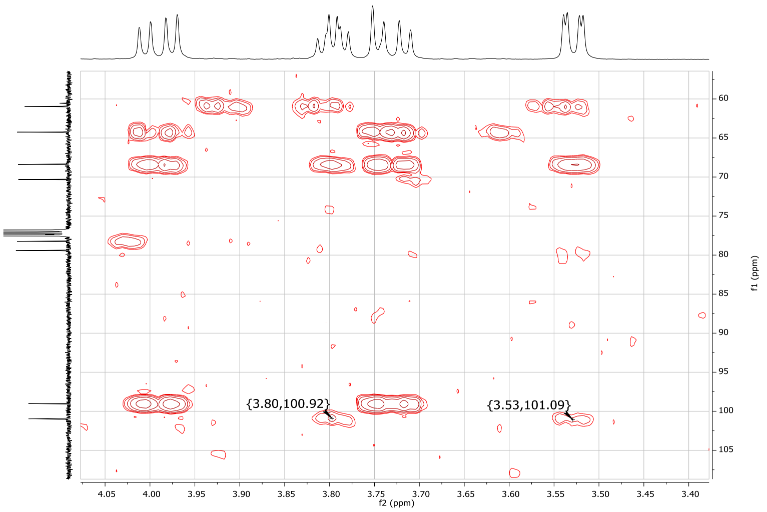

Figure S1. ^{1}H,^{13}C-HMBC spectrum (101 MHz, $CDCl_3$) of the isolated side product showing the coupling of H5 and H7 with the same quaternary carbon from an isopropylidene group.

Screening of dihydroxylation conditions and octenitol substrates

Scheme S1. Dihydroxylation of the octenitol substrates.

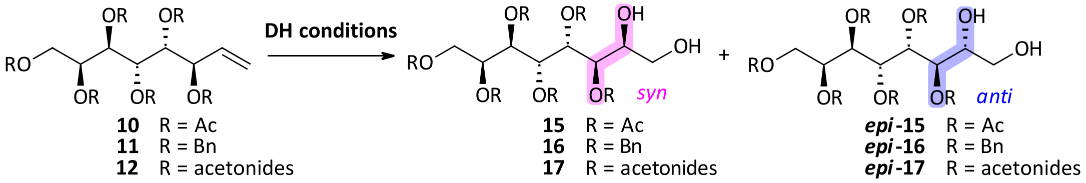

General procedure for the Upjohn dihydroxylation conditions

The protected enitol (0.10-0.22 mmol) was suspended in *t*-BuOH/H_2O (1:1, 0.1-0.3 M) at rt. Then, NMO·H_2O (1.0 equiv) was added, followed by $K_2[OsO_2(OH)_4]$ (1.0 mol%). For octenitol **11** and **12**, DCM (final solvent ratio 1:1:1) was added, too. Reaction monitoring was performed via TLC (LP/Et_2O) and LC-MS. As soon as complete consumption of the starting material was observed (1-3 days), the reaction mixture was quenched by the addition of solid $Na_2S_2O_5$. After further stirring for 1 h, the mixture was diluted with H_2O and the aq. phase was extracted with DCM or EtOAc. The pooled organic phase was washed with brine, dried over anhydrous Na_2SO_4 and evaporated to dryness. The partly protected sugar alcohols were obtained in yields >95% and directly submitted to the deprotection step.

General procedure for the Sharpless dihydroxylation conditions

Preparation of AD-mix-α (×3) or AD-mix-β (×3):
The three times concentrated AD-mix was prepared as follows. In a mortar, $K_3Fe^{III}(CN)_6$ (1.50 g, 3.00 equiv), K_2CO_3 (631 mg, 3.00 equiv), $K_2[OsO_2(OH)_4]$ (3.4 mg, 0.6 mol%) and $(DHQ)_2PAL$ (**α**, 36 mg, 3.0 mol%) or $(DHQD)_2PHAL$ (**β**, 36 mg, 3.0 mol%) were grinded until a homogeneous orange powder was obtained. The catalyst enriched AD-mixes are stated as AD-mix-α (×3) and AD-mix-β (×3), respectively.

AD-mix-α (×3) or AD-mix-β (×3) (1.4 g/mmol substrate) was suspended in *t*-BuOH/H_2O (1:1, 0.4 M (with respect to the substrate)) and $MsNH_2$ (1.0 equiv) was added at rt. After stirring for 1 h, the protected enitol was added as a solution in DCM (0.8 M). Reaction monitoring was performed via TLC (LP/Et_2O) and LC-MS. As soon as complete consumption of the starting material was observed (days), the reaction mixture was quenched by the addition of solid $Na_2S_2O_5$. After further stirring for 1 h, the mixture was diluted with H_2O and the aq. phase was extracted with DCM. The pooled organic phase was washed with brine, dried over anhydrous Na_2SO_4 and evaporated to dryness. The partly protected sugar alcohols were obtained in yields >95% and directly submitted to the deprotection step.

Scheme S2. Deprotection of the partly protected octitol mixtures toward the free octitols.

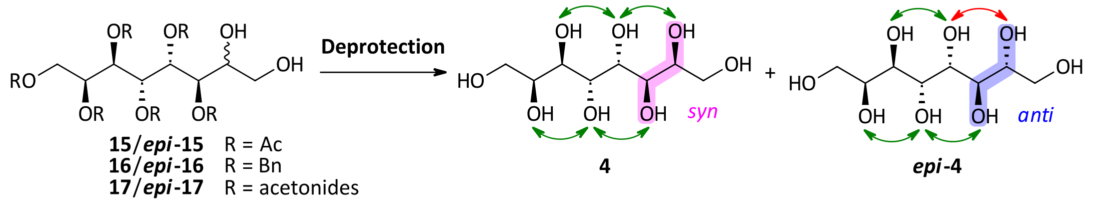

General procedure for the deacetylation of octitol mixture 15 / *epi*-15

The crude mixture **15** / ***epi*-15** from the individual dihydroxylation reaction was taken up in MeOH (HPLC grade, 0.1 M) and NaOMe (30% in MeOH) was added dropwise at rt until pH was about 9-10. Upon complete conversion to fully unprotected octitols (monitoring via LC-MS), water was added to dissolve precipitated product and the reaction mixture was neutralized by the addition of Dowex-H^+ resin (MeOH washed). After filtration, the solution was vaporized, giving a colorless to beige solid. An NMR-sample (~5-10 mg) was prepared in D_2O (~0.6 mL) and ^{13}C-NMR analysis was performed to determine the ratio of the two isomers **4** and ***epi*-4** via integration of the detected signals.

General procedure for the catalytic hydrogenation of octitol mixture 16 / *epi*-16

The crude mixture **16** / ***epi*-16** from the dihydroxylation reaction was taken up in MeOH (HPLC grade, 0.1 M) and atmosphere was changed to Ar. Then, Pd/C (10 *w*%, 10% *w*:*w* with respect to the starting material) and the reaction mixture was stirred under hydrogen atmosphere (1 atm) using a balloon. Upon complete conversion to fully unprotected octitols (monitoring via LC-MS), water was added to dissolve precipitated product and the reaction mixture was filtered over a pad of Celite®. The filtrate was vaporized, giving a colorless to beige solid. An NMR-sample (~5-10 mg) was prepared in D_2O (~0.6 mL) and ^{13}C-NMR analysis was performed to determine the ratio of the two isomers **4** and ***epi*-4** via integration of the detected signals.

General procedure for the cleavage of acetonide protecting groups of crude octitol mixture 17 / *epi*-17

The crude mixture **17** / ***epi*-17** from the dihydroxylation reaction was taken up in MeOH (0.3 M) and Dowex-H^+ resin (MeOH washed, 20% *w*:*w* with respect to the starting material) was added. The reaction mixture was stirred at 80 °C until LC-MS indicated complete conversion to fully unprotected octitols. The mixture was filtered over a pad of Celite®, washed with water, and the filtrate was vaporized, giving a colorless to beige solid. An NMR-sample (~5-10 mg) was prepared in D_2O (~0.6 mL) and ^{13}C-NMR analysis was performed to determine the ratio of the two isomers **4** and ***epi*-4** via integration of the detected signals.

Analysis of crude entiol mixture toward decenitol 10

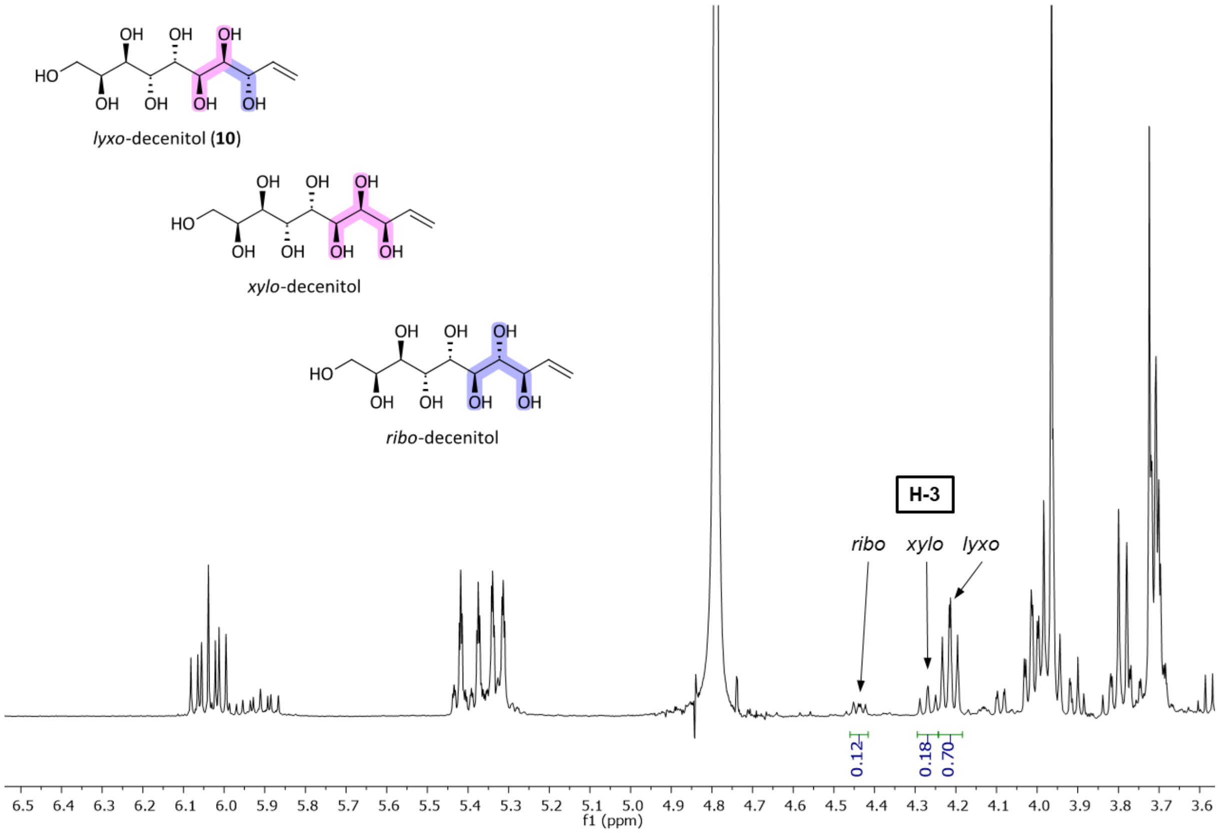

Figure S2. ^{1}H-NMR (400 MHz, D_2O) of crude enitol fraction from optimized IMA of heptose **11**.

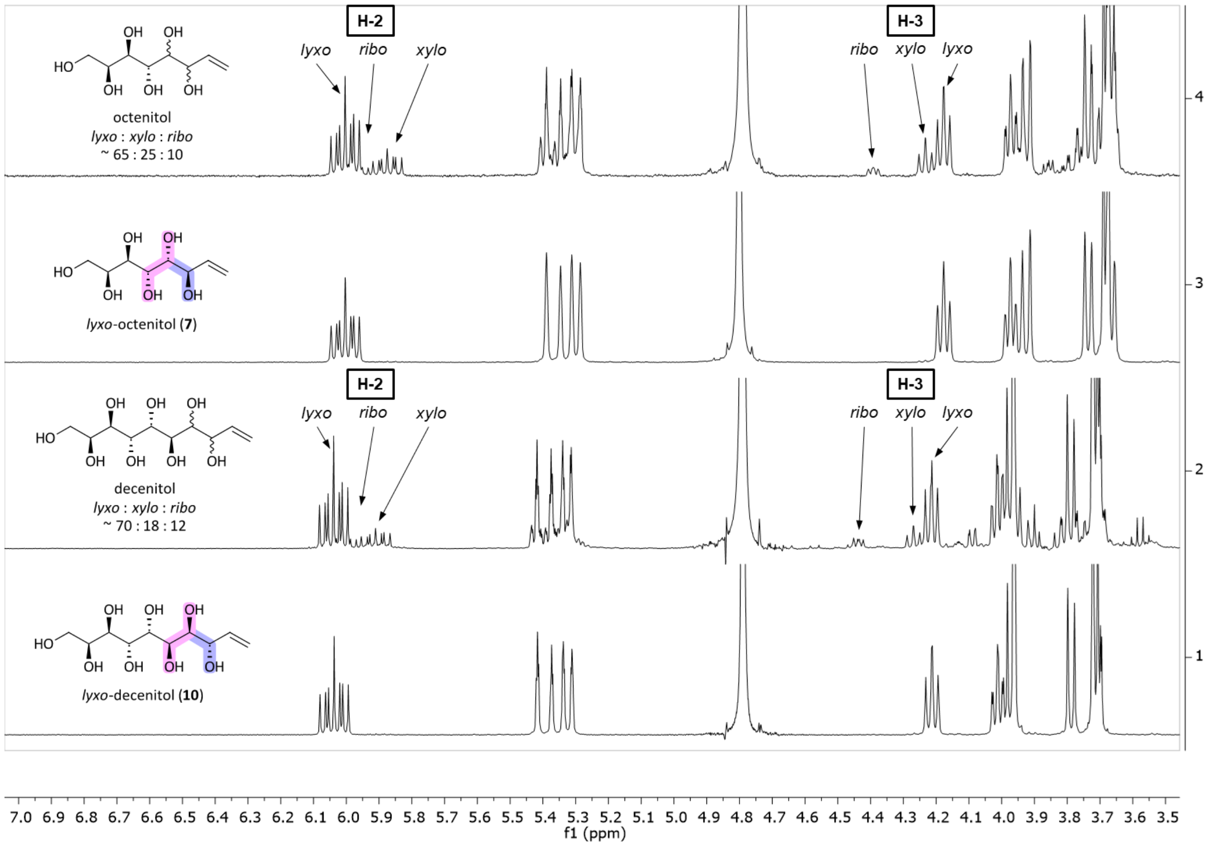

Figure S3. Comparison of ^{1}H-NMR (400 MHz) of crude octenitol fraction and crude decenitol fraction.

Attempted synthesis toward the L-*glycero*-D-*manno*-D-*manno*-dodecenitol SI-3

The newly developed protocol for the IMA of less reactive aldoses was applied in the attempted elongation of the C9-sugar L-*lyxo*-L-*manno*-nonose (**SI-1**) that was obtained from the corresponding L-*lyxo*-L-*manno*-decenitol **10** via ozonolysis. Nevertheless, the IMA of the nonose was not successful in respect to the obtained yields (only 11% for the desired isomer) using the Grignard-type protocol, even with increased amounts of reagents. Further, the reaction could not be boosted to higher conversions even after purification of the nonose **SI-1** by acetylation (**SI-2**) and deacetylation. The IMA was carried out on a scale of 0.18 mmol using the two-step protocol with an excess of indium (4 equiv) and pivalate reagent **21** (6 equiv). Upon acetylation of the crude mixture (Ac_2O, pyridine) the protected enitol species (mixture of isomers) was isolated in 26% yield via column chromatography. Unreacted aldose was recovered in the peracetylated form **SI-2** in 58% yield. Upon deprotection, the tentative *lyxo*-isomer **SI-3** was found to again be the major component (~70:30 *dr lyxo*:other isomers, according to ^{1}H-NMR) and was isolated upon trituration with an overall yield of 11%, starting from the nonose **SI-1**.

Scheme S3. Synthetic route toward the L-*glycero*-D-*manno*-D-*manno*-dodecenitol **SI-3**.

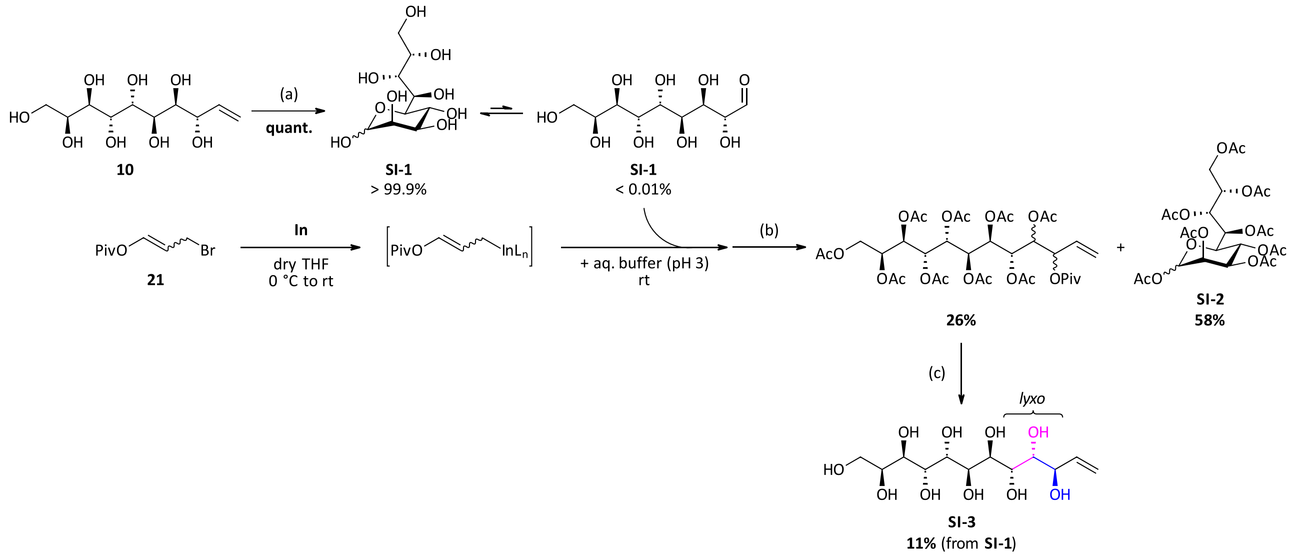

(a) O_3, H_2O/acetone (3:2), Sudan red III (cat.), 0 °C, 10 min; then PPh_3, rt, 12 h; (b) Ac_2O, DMAP, pyridine, rt, 16 h; (c) NaOMe (30% in MeOH), MeOH, rt, 3 h; trituration (MeOH, then H_2O).

From the dodecenitol **SI-3**, the reaction sequence developed for the *galacto*-octitol **4** and *galacto*-decitol **6** with the dihydroxylation, giving **SI-4**, and Mitsunobu reaction as the two key-steps could in theory be applied to obtain the C12 sugar alcohol *galacto*-dodecitol **SI-5**. However, due to the observed low amounts and the observed instability of the decitol in the STA measurements these steps were not conducted within this work. Alternative synthetic strategy toward higher carbon sugar alcohols is currently under development.

Scheme S4. Theoretical synthesis of the *galacto*-dodecitol **SI-5** following the developed reaction sequence.

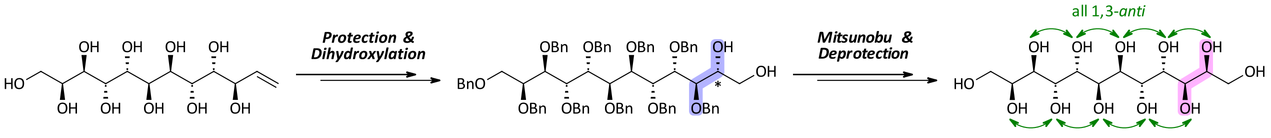

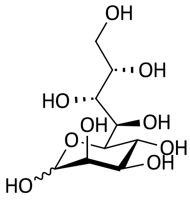

SI-1

L-*Lyxo*-L-*manno*-nonose (SI-1)

Following the protocol for the synthesis of the LD-heptose **11**, the L-*lyxo*-L-*manno*-decenitol **10** (307 mg, 1.14 mmol, 1.00 equiv) was dissolved in a water/acetone mixture (4:1, 25 mL) and a small amount of Sudan red (II) in acetone was added, staining the reaction mixture pink (for indication). The solution was cooled to 0 °C using an ice-bath, and ozone was bubbled through using a gas inlet tube. At the outlet, the gas was passed through a gas washing bottle with aq. KI (10% w/w) solution. Bubbling of ozone was performed until the pink color diminished and TLC analysis ($CHCl_3$/MeOH/H_2O 14:7:1) indicated full conversion of the starting material to a more polar spot. The ozone generator was switched off and oxygen was bubbled through the solution for ~15 min, followed by bubbling with Ar (10 min). PPh_3 (600 mg, 2.29 mmol, 2.00 equiv) was added followed by further acetone (to dissolve PPh_3) and stirring was continued at rt overnight. The next day, the reduction of all peroxides and H_2O_2 was confirmed (peroxide test stripe) and the reaction mixture was concentrated. The remaining aqueous layer was washed with DCM (2×50 mL), EtOAc (50 mL) and Et_2O, concentrated *in vacuo* and co-evaporated from MeOH twice. The nonose **SI-1** (344 mg, quant.) was obtained as a colorless, viscous oil as an anomeric mixture with a ratio of α/β = 1.8:1 (^{1}H-NMR).

^{1}H-NMR (600 MHz, D_2O) δ 5.19 (d, J = 1.6 Hz, 1.8H, H1α), 4.90 (d, J = 1.1 Hz, 1H, H1β), 4.03 – 3.97 (m, 6.4H, H6α, H7α, H8α, H6β), 3.96 – 3.94 (m, 1H, H8β), 3.94 (d, J = 1.1 Hz, 1H, H2β), 3.93 (dd, J = 2.8, 1.6 Hz, 1.8H, H2α), 3.90 – 3.86 (m, 3.6H, H4α, H3α), 3.82 (t, J = 9.8 Hz, 1H, H4β), 3.76 (dd, J = 9.6, 1.5 Hz, 1H, H7β), 3.74 – 3.65 (m, 8.4H, H9α, H5α, H9β, H3β), 3.55 (dd, J = 9.8, 1.3 Hz, 1H, H5β); **^{13}C{^{1}H}-NMR (101 MHz, D_2O)** δ 94.8 (C1α), 94.5 (C1β), 75.2 (C5β), 73.9 (C3β), 71.8 (C2β), 71.6 (C5α), 71.3 (C2α), 71.2 (C3α), 69.4 (C6α), 69.3 (C6β), 66.8 (C4α), 66.5 (C4β), 63.6 (C7α), 63.4 (C7β) ppm; **HRMS (ESI)** *m/z* [M-H]$^-$ calc. for $C_9H_{17}O_9$: 269.0873, found:269.0881.

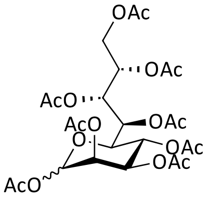

SI-2

1,2,3,4,6,7,8,9-Octa-*O*-acetyl-L-*lyxo*-L-*manno*-nonose (SI-2)

For further purification, the nonose **SI-1** (400 mg, 1.48 mmol, 1.00 equiv) that was obtained from the ozonolysis was taken up in pyridine (5.0 mL) and acetic anhydride (3,63 g, 3.36 mL, 35.5 mmol, 24.0 equiv) was added to the stirring mixture under ice-bath cooling. After 15 min a spatula tip of DMAP was added. TLC after 2 h ($CHCl_3$/MeOH/H_2O 14:7:1, LP/EtOAc 1:1) indicated complete conversion to a very nonpolar species. Excessive reagent was quenched by the addition of MeOH (1.5 mL) under ice-bath cooling, and stirring was continued for 30 min. The mixture was diluted with EtOAc (50 mL) and extracted with 1N HCl (3×20 mL – until pH remained acidic). The organic phase was further washed with water (20 mL), aq. sat. $NaHCO_3$ (40 mL) and brine and dried over anhydrous Na_2SO_4. The solution was evaporated, giving a yellow, oily crude material (0.7 g). This was purified via flash column chromatography (45.5 g SiO_2, LP/EtOAc 5:1 → 1:1) giving the product **SI-2** as a white crystalline solid (675 mg, 75%) as an anomeric mixture with a ratio of α/β = 2:1 (^{1}H-NMR).

^{1}H-NMR (600 MHz, $CDCl_3$) δ 6.15 (d, *J* = 1.7 Hz, 2H, H1α), 5.77 (d, *J* = 1.1 Hz, 1H, H1β), 5.59 (dd, *J* = 9.4, 1.9 Hz, 2H, H6α), 5.52 (dd, *J* = 9.9, 1.9 Hz, 1H, H6β), 5.44 (dd, *J* = 3.3, 1.0 Hz, 1H, H2β), 5.42 (dd, *J* = 9.9, 2.4 Hz, 1H, H7β), 5.32 – 5.29 (m, 2H, H3α), 5.27 – 5.23 (m, 4H, H7α, H8α), 5.22 (dd, *J* = 3.5, 1.8 Hz, 2H, H2α), 5.19 – 5.12 (m, 2H, H4β, H8β), 5.12 – 5.07 (m, 3H, H4α, H3β), 4.25 (dd, *J* = 11.7, 5.2 Hz, 1H, H9a (β)), 4.20 (dd, *J* = 11.4, 6.0 Hz, 2H, H9a (α)), 3.94 (dd, *J* = 10.3, 1.3 Hz, 2H, H5α), 3.89 – 3.82 (m, 3H, H9b (α), H9b (β)), 3.77 (dd, *J* = 10.0, 2.4 Hz, 1H, H5β), 2.23 (s, 3H), 2.17 (s, 6H), 2.16 (s, 3H), 2.15 (s, 6H), 2.13 (s, 6H), 2.11 (s, 3H), 2.09 – 2.08 (m, 9H), 2.06 (s, 3H), 2.05 – 2.04 (m, 12H), 2.01 (s, 9H), 2.00 (s, 3H), 1.98 (s, 9H); **$^{13}C\{^1H\}$-NMR (151 MHz, $CDCl_3$)** δ 170.9 – 167.5 (***C***OCH$_3$), 90.5 (C1α), 90.4 (C1β), 73.0 (C5β), 71.5 (C3β), 69.9 (C5α), 69.5 (C3α), 68.8 (C2α), 68.4 (C2β), 68.1 (C8β), 67.9 (C8α), 67.8 (C6β), 67.7 (C6α), 65.9 (C7α), 65.5 (C7β), 64.7 (C4α), 64.3 (C4β), 62.3 (C9β), 61.6 (C9α), 21.0 – 20.6 (CO***C***H$_3$) ppm; **HRMS (ESI)** *m*/*z* $[M+Na]^+$ calc. for $C_{25}H_{34}NaO_{17}$: 629.1694, found:629.1700.

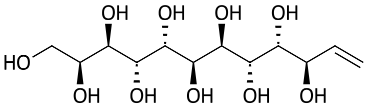

SI-3

1,2-Dideoxy-L-*glycero*-D-*manno*-D-*manno*-dodecenitol (SI-3)

Step 1 – IMA: indium (85 mg, 0.74 mmol, 4.0 equiv) was weighed in a flame dried Schlenk flask and Schlenk technique was applied. Anhydrous THF (1.0 mL) was added, and the mixture was cooled to 0 °C using an ice-bath. 3-Bromopropenyl pivalate (**21**) (0.25 g, 1.1 mmol, 6.0 equiv) was added dropwise to the vigorously stirring mixture. After 15 min, the ice-bath was removed, and the suspension was allowed to warm up to room temperature and stirred for another 45 min. Then, L-*lyxo*-L-*manno*-nonose (**SI-1**) (50 mg, 0.19 mmol, 1.0 equiv) was added to the pre-formed reagent as a solution in phthalate buffer (pH ~2.9, 0.2 mL). TLC ($CHCl_3$/MeOH/H_2O 14:7:1) indicated conversion to the enitol species but also showed unreacted starting material (after 45 min). The reaction mixture was diluted with MeOH and H_2O and filtered. The filtrate was concentrated *in vacuo*, giving a colourless solid.

Step 2 – acetylation: this material was taken up in pyridine (99%, 3 mL), and Ac_2O (0.85 g, 0.79 mL, 8.3 mmol, 45 eq.) was added to the stirring mixture under ice-bath cooling. After 10 min, the ice-bath was removed, and a spatula of DMAP was added. Stirring at rt was continued overnight. The next day, LC-MS indicated complete conversion to the fully protected dodecenitol and nonose, so excessive reagent was quenched by the addition of MeOH (0.5 mL) under ice-bath cooling, and the mixture was stirred for further 15 min. After diluting with EtOAc (50 mL), the organic phase was extracted with ice-cold 1N HCl (2×25 mL – until pH remained acidic). After back extraction (2×20 mL EtOAc), the pooled organic phase was washed with water (20 mL), aq. sat. $NaHCO_3$ (20 mL) and brine. After drying over anhydrous Na_2SO_4, the solution was evaporated to dryness (124 mg). From this, the protected dodecenitol mixture could be isolated via column chromatography (12.5 g SiO_2, LP/EtOAc 3:1 → 1:1) (38 mg, 26%) as a yellow oil and the nonose peracetate **SI-2** was recovered (65 mg, 58%).
Step 3 – deacetylation: the dodecenitol peracetate was taken up in MeOH (HPLC grade, 0.5 mL) and NaOMe (30% in MeOH) was added under stirring at rt until pH was about 11. Reaction monitoring via LC-MS indicated full conversion after 3 hours. The reaction mixture was neutralized by the addition of Dowex-H^+ (MeOH washed), H_2O was added to dissolve the precipitated enitols, and the solution was filtered. Evaporation of the solvent gave a beige solid matter (20 mg) that is a mixture of dodecenitol diastereomers with a ratio of *lyxo* : xylo : ribo = 71 : 22 : 7. The solid was then triturated in MeOH (1 mL), and the remaining solid was isolated via centrifugation. The residue was again washed with MeOH (2×1 mL), and further triturated in H_2O (2×1 mL). After washing with MeOH (1 mL) and Et_2O (1 mL), the remaining white solid was dried at 50 °C *in vacuo*, giving L-*glycero*-D-*manno*-D-*manno*-dodecenitol **SI-3** (7 mg, 85% purity (15% *xylo*-isomer), 11% yield from **SI-1**) with small amounts of remaining tentative *xylo*-isomer.

^{1}H-NMR (600 MHz, D_2O) δ 6.03 (ddd, *J* = 17.3, 10.5, 6.9 Hz, 1H, H2), 5.39 (dt, *J* = 17.1, 1.4 Hz, 1H, H1a), 5.32 (dt, *J* = 10.5, 1.3 Hz, 1H, H1b), 4.21 (dd, *J* = 8.1, 6.9 Hz, 1H, H3), 4.03 – 4.00 (m, 1H, H11), 3.99 – 3.95 (m, 5H, H5, H6, H7, H8, H9), 3.79 (d, *J* = 8.1 Hz, 1H, H4), 3.72 – 3.70 (m, 3H, H12, H10) ppm;. **HRMS (ESI)** *m/z* $[M-H]^-$ calc. for $C_{12}H_{23}O_{10}$: 327.1291, found:327.1296.
Due to the low solubility of the final product in the used solvent (D_2O), no ^{13}C-NMR spectrum could be recorded.

The open-chain contents of heptose 11 and nonose SI-1

The investigated heptose **11** and even more so nonose **SI-1** showed substantially reduced reactivity in the IMA reaction compared to shorter analogues. We were interested in the open-chain content of these sugars since the availability of the free aldehyde moiety is assumed to impact the reactivity of the sugar. The OCC-values were determined using a kinetic photometric assay, developed by our lab, in which the formation of an adduct between the aldose and ABAO is followed.[6] The results of the measurements are shown in Table S1 together with the OCC of D-lyxose for comparison, also determined using the same assay.

Table S1. Measured OCCs using the reported ABAO assay[6].

Entry	Name	OCC (%)
**1**	D-lyxose (***ent*-8**)	0.11
**2**	D-mannose	0.032
**3**	L-*glycero*-D-*manno*-heptose (**11**)	0.0344
**4**	L-*lyxo*-L-*manno*-nonose (**SI-1**)	0.0343

Surprisingly, the OCCs of the heptose **11** (Entry 3) and nonose **SI-1** (Entry 4) were quite similar and in the range of the value of D-mannose but, as expected, significantly lower than the OCC of D-lyxose. However, as the nonose **SI-1** was shown to be less reactive in the IMA than the heptose **11** the OCC cannot be the only limiting effect on the IMA performance that is observed with increasing carbon chain length.

General procedure for the ABAO assay used for the determination of the OCCs

Scheme S5. Formation of the ABAO-adduct with a reducing sugar that is UV-active.

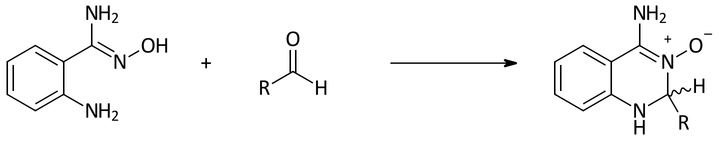

To a solution of 2-aminobenzamidoxime (ABAO, **SI-6**, 42.1 mM) in NH_4OAc buffer (190 µL, 100 mM, pH 4.5) in a 96-well plate (Greiner, 96-well microplate, PS, F-bottom, clear) with lid (Greiner, with condensation rings, PS, high profile, clear), an aqueous solution of the respective sugar (10 µL, 80 mM) in H_2O was added. The plate was shaken in the plate reader, and measurements were conducted at 405 nm for 3 days at 25 °C.

Blank samples with pure water (10 µL) instead of the sugar solution were performed and considered, too. All reactions and the blank samples were performed in triplicates.

The determination of the OCC values was performed according to Kalaus et al.[6] by fitting the curves of formation observed in the UV-measurement to pseudo-first order kinetics and calibrating against reference tetroses with high and simply measurable OCCs.

STA / DSC-measurements

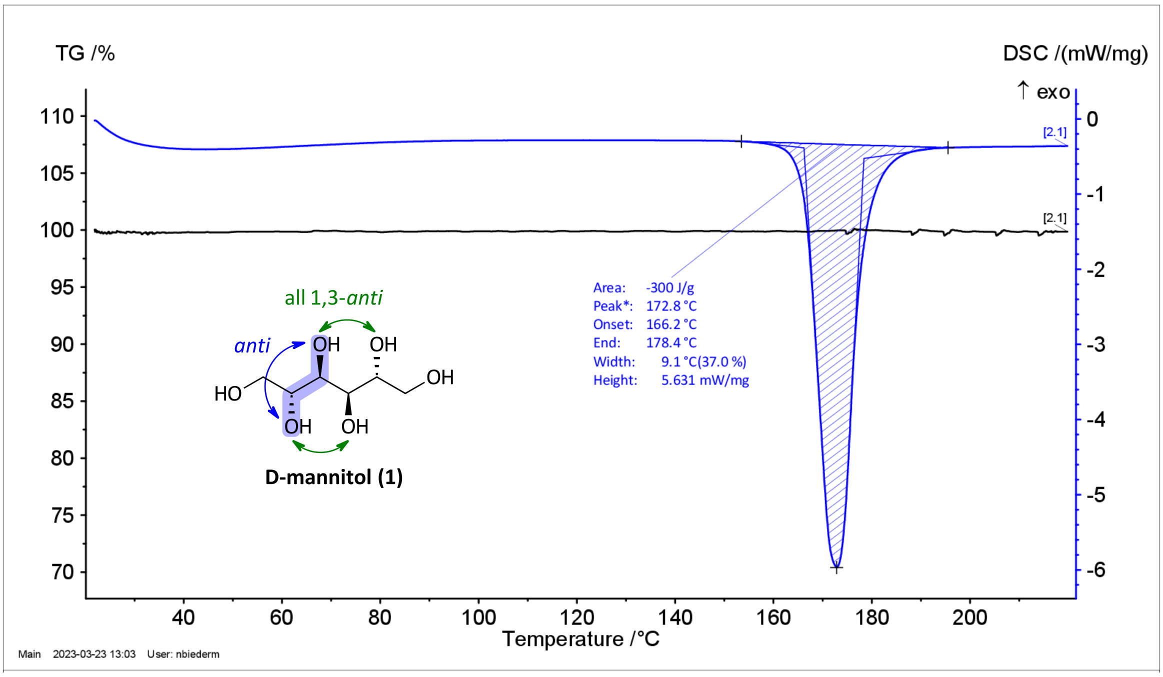

Figure S4. STA measurement of D-mannitol (**1**), that was recrystallized from H_2O.

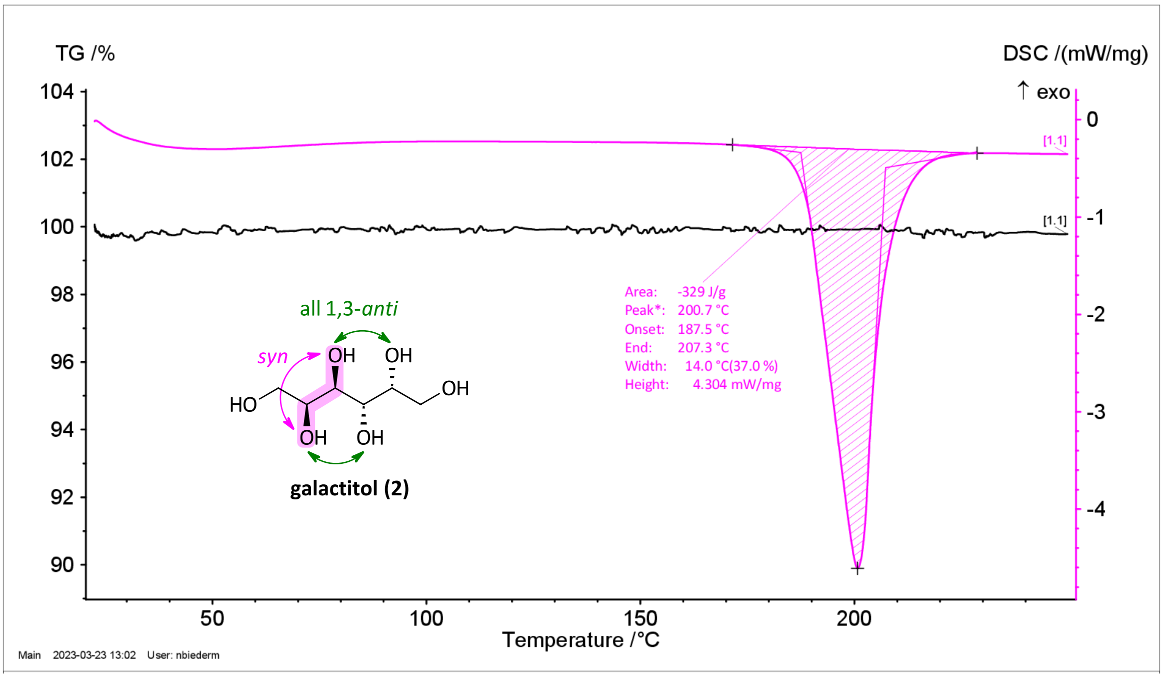

Figure S5. STA measurement of galactitol (**2**), that was recrystallized from H_2O.

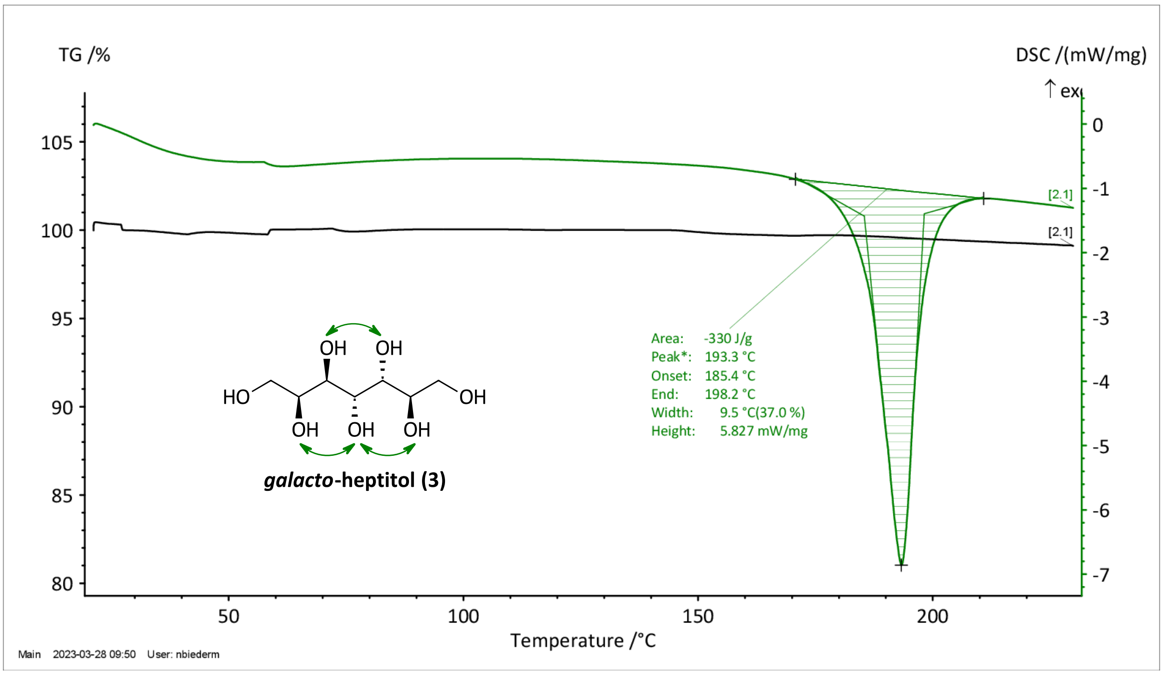

Figure S 6. STA measurement of *galacto*-heptitol **3**, that was recrystallized from MeOH/H_2O (4:1).

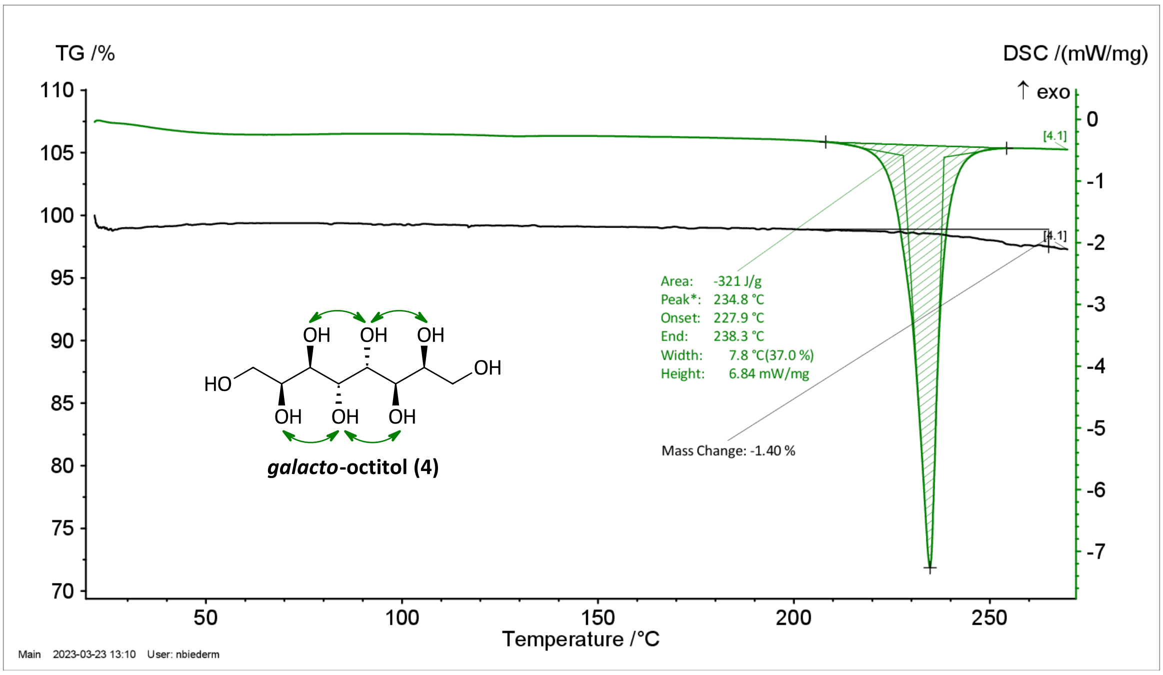

Figure S7. STA measurement of *galacto*-octitol **4**, that was recrystallized from MeOH/H_2O (4:1).

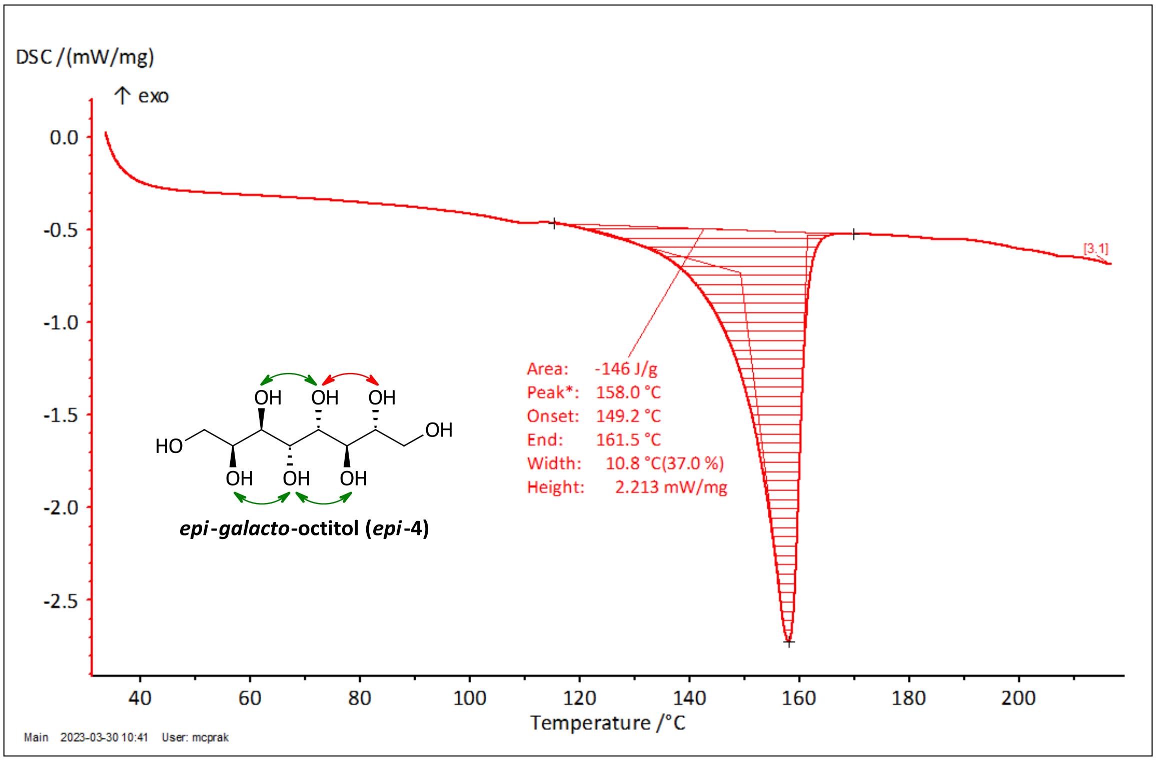

Figure S8. DSC measurement of *epi*-*galacto*-octitol ***epi*-4**, that was recrystallized from MeOH/H_2O (4:1).

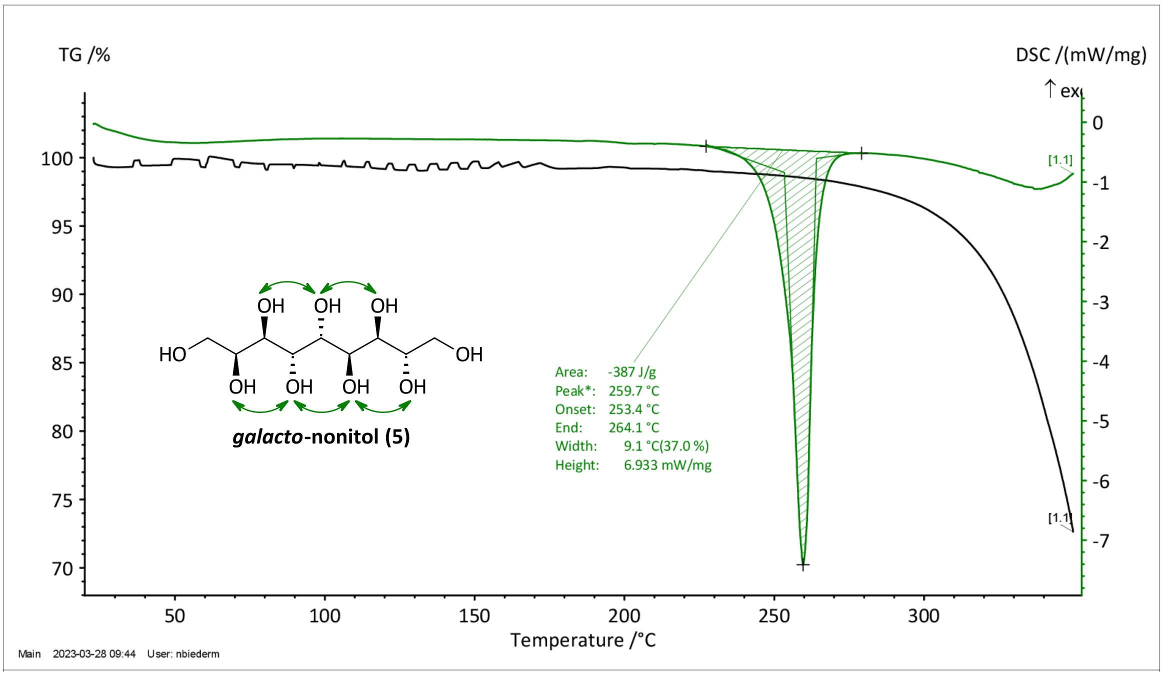

Figure S9. STA measurement of *galacto*-nonitol **5**, that was recrystallized from H_2O.

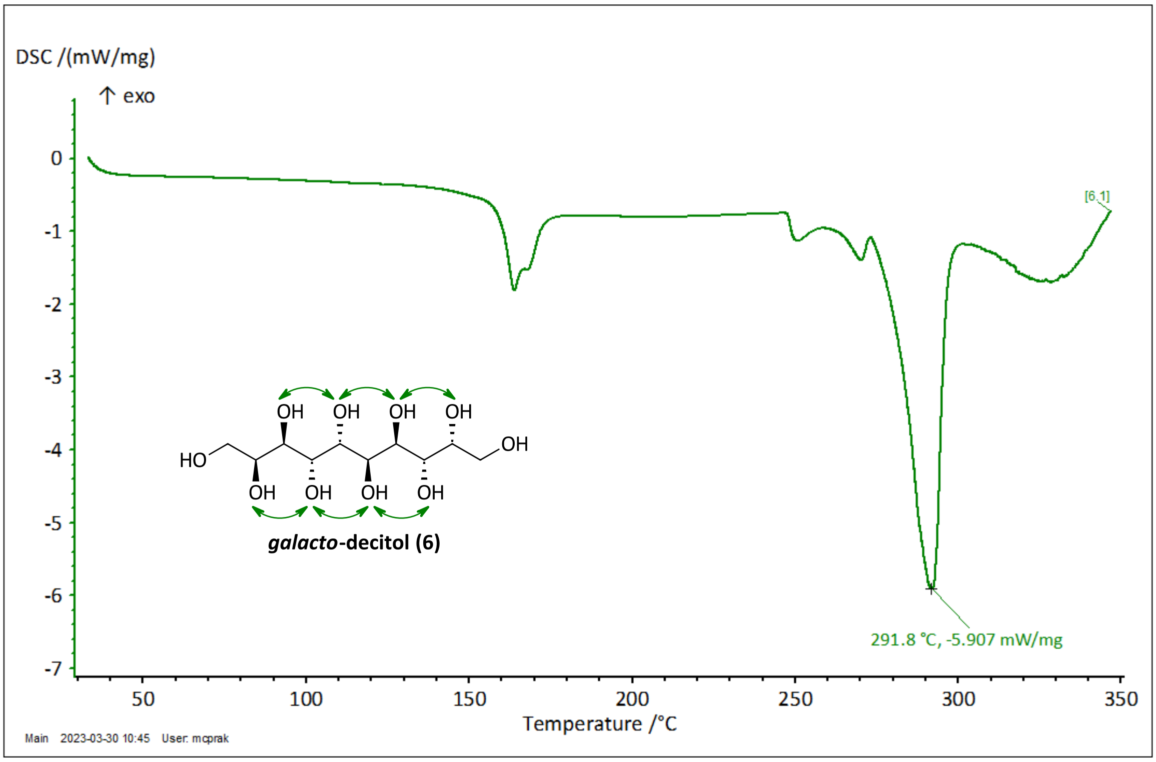

Figure S10. DSC measurement of *galacto*-decitol **6**, that was recrystallized from H_2O.

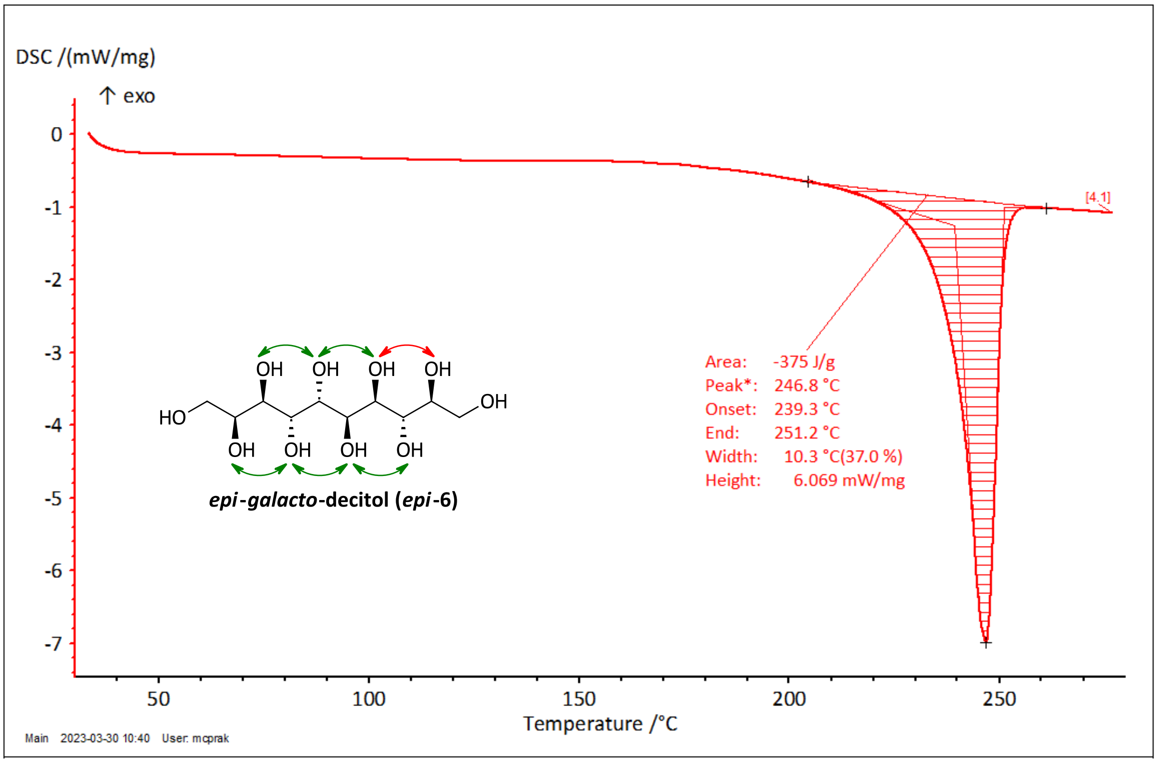

Figure S11. DSC measurement of *epi*-*galacto*-decitol ***epi*-6**, that was recrystallized from H_2O.

Spectra

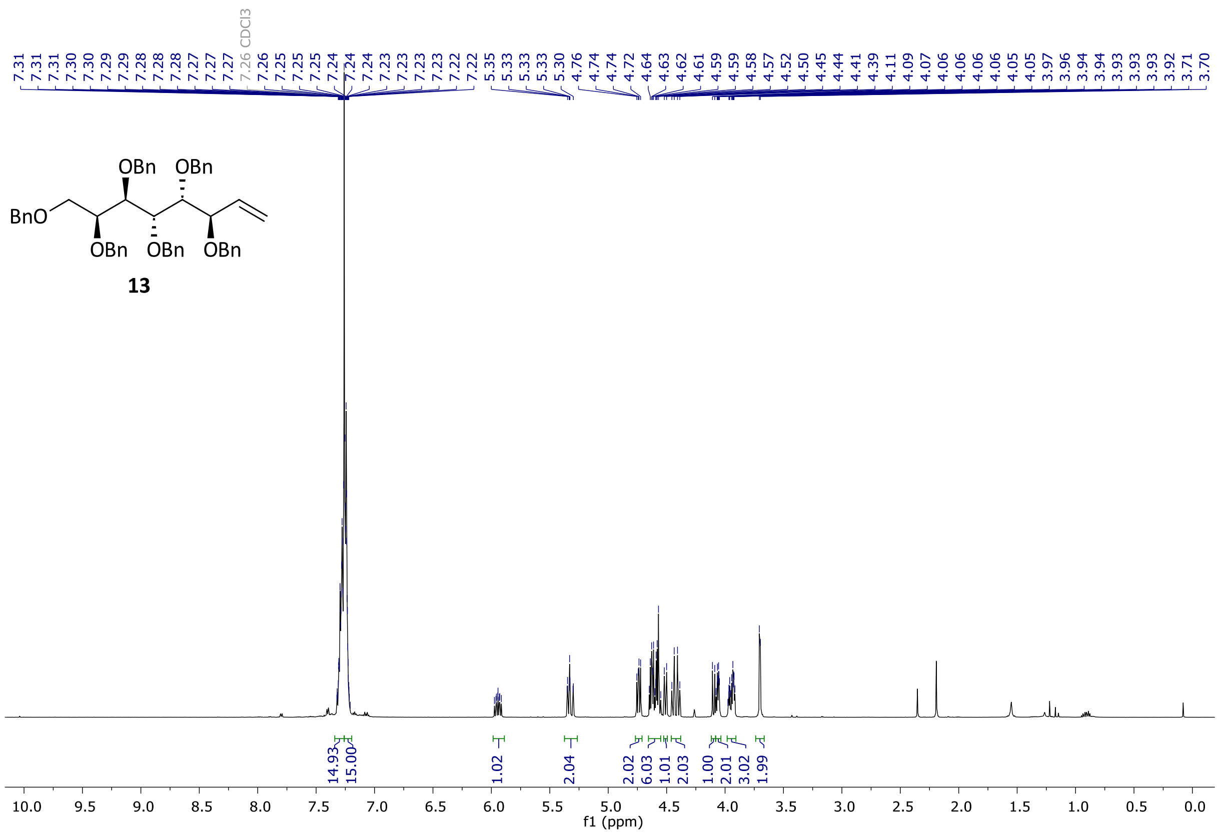

Figure S12. ^{1}H-NMR (600 MHz, $CDCl_3$) of 3,4,5,6,7,8-hexa-*O*-benzyl-1,2-dideoxy-L-*glycero*-D-*manno*-oct-1-enitol (**13**).

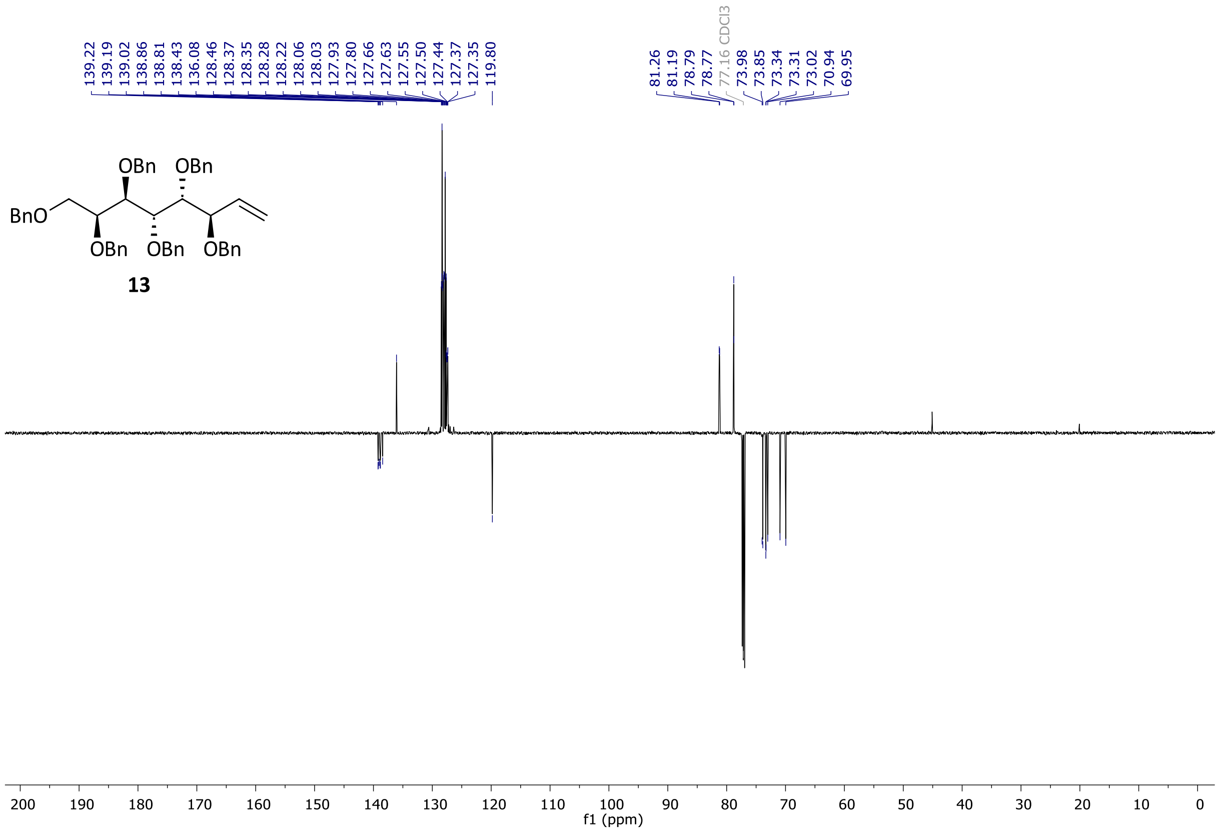

Figure S13. $^{13}C\{^{1}H\}$-NMR (151 MHz, $CDCl_3$) of 3,4,5,6,7,8-hexa-*O*-benzyl-1,2-dideoxy-L-*glycero*-D-*manno*-oct-1-enitol (**13**).

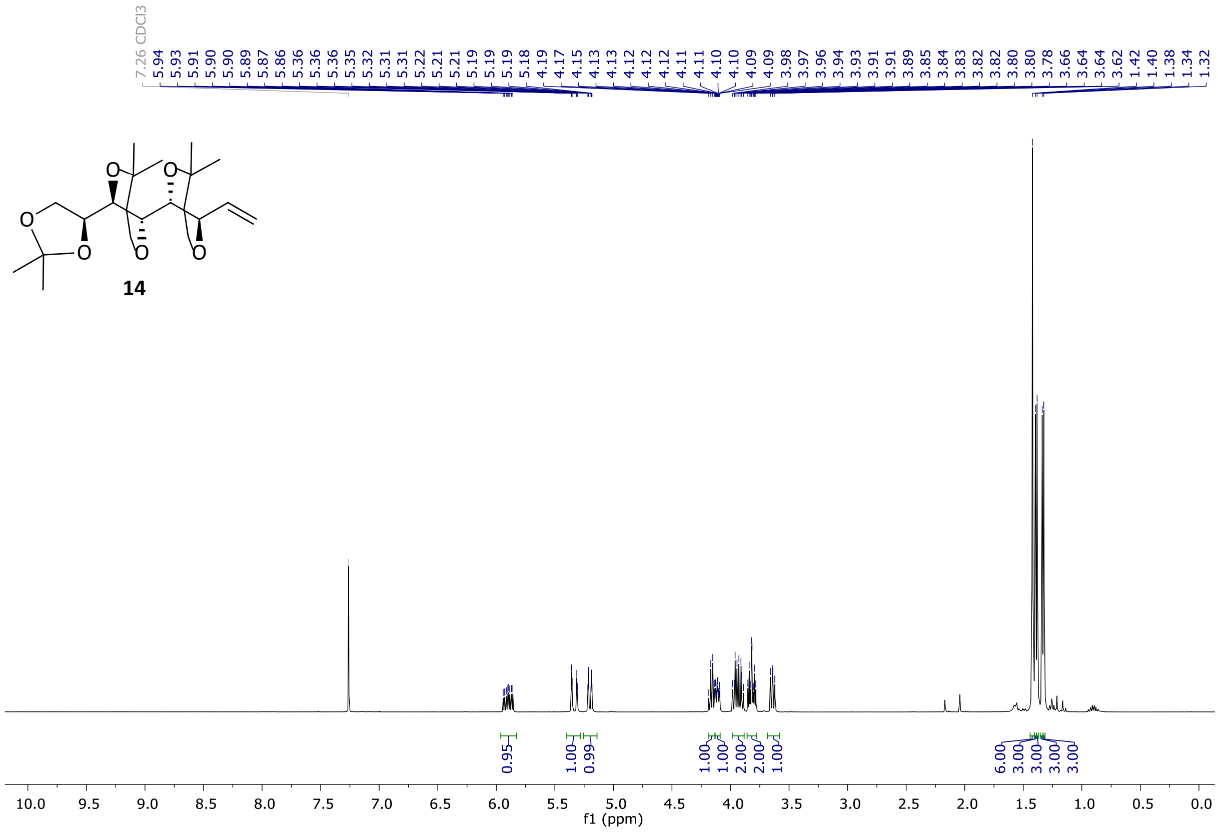

Figure S14. ^{1}H-NMR (400 MHz, $CDCl_3$) of 1,2-dideoxy-3,4:5,6:7,8-tri-*O*-isopropylidene-L-*glycero*-D-*manno*-oct-1-enitol (**14**).

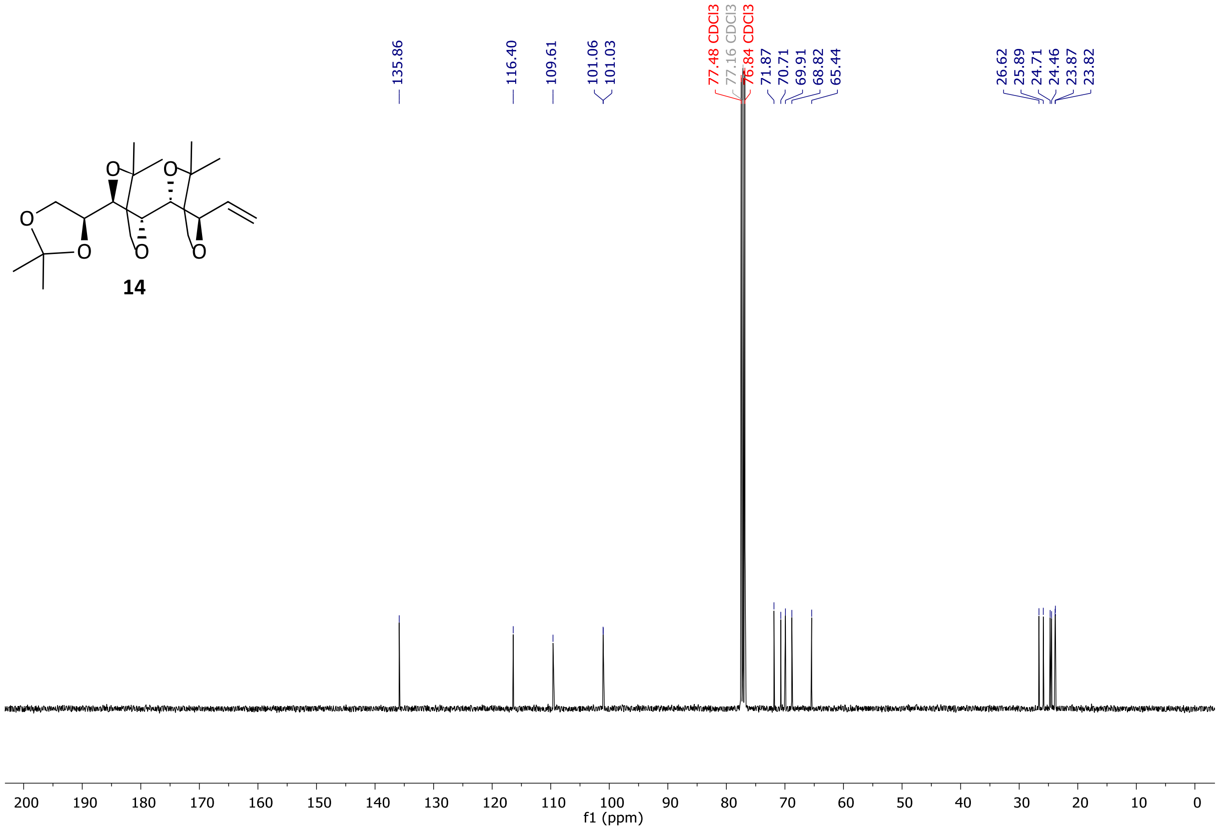

Figure S15. ^{13}C{^{1}H}-NMR (101 MHz, $CDCl_3$) of 1,2-dideoxy-3,4:5,6:7,8-tri-*O*-isopropylidene-L-*glycero*-D-*manno*-oct-1-enitol (**14**).

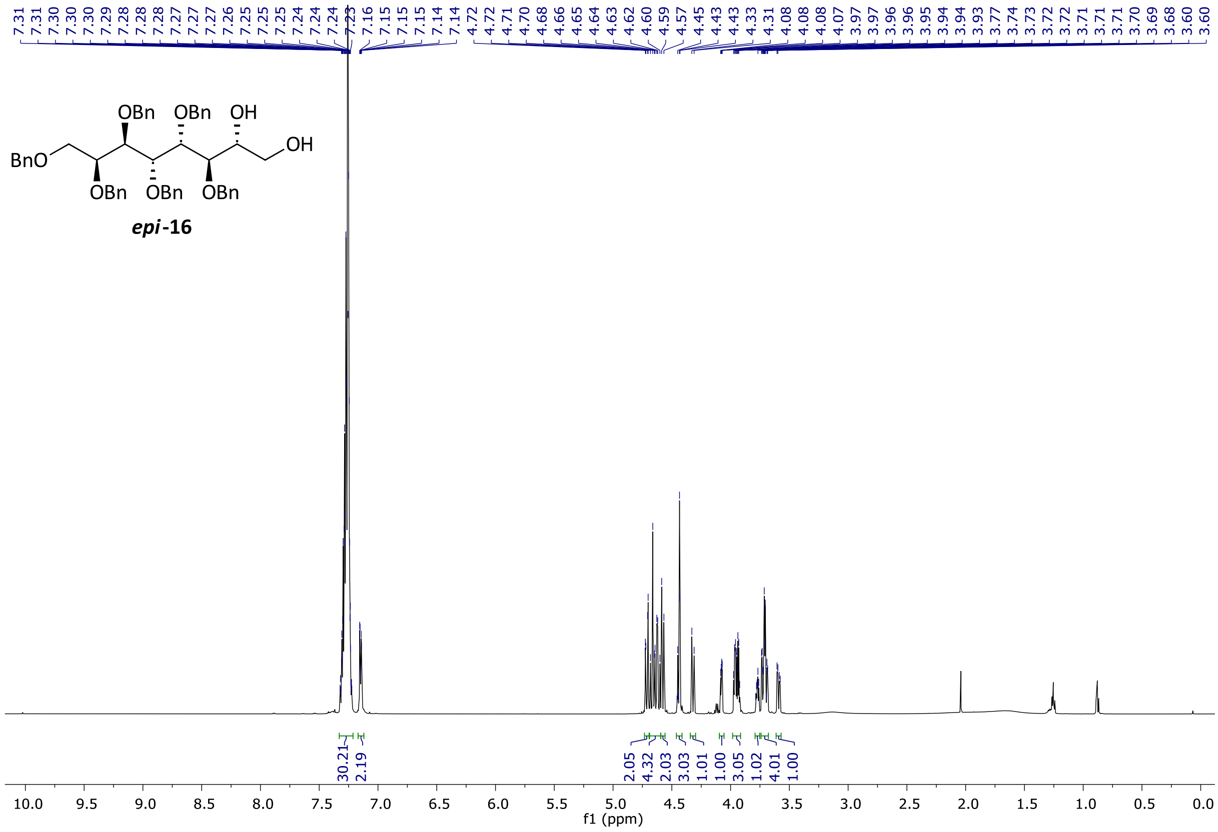

Figure S16. ^{1}H-NMR (600 MHz, $CDCl_3$) of 3,4,5,6,7,8-hexa-*O*-benzyl-L-*threo*-D-*talo*-octitol (***epi*-16**).

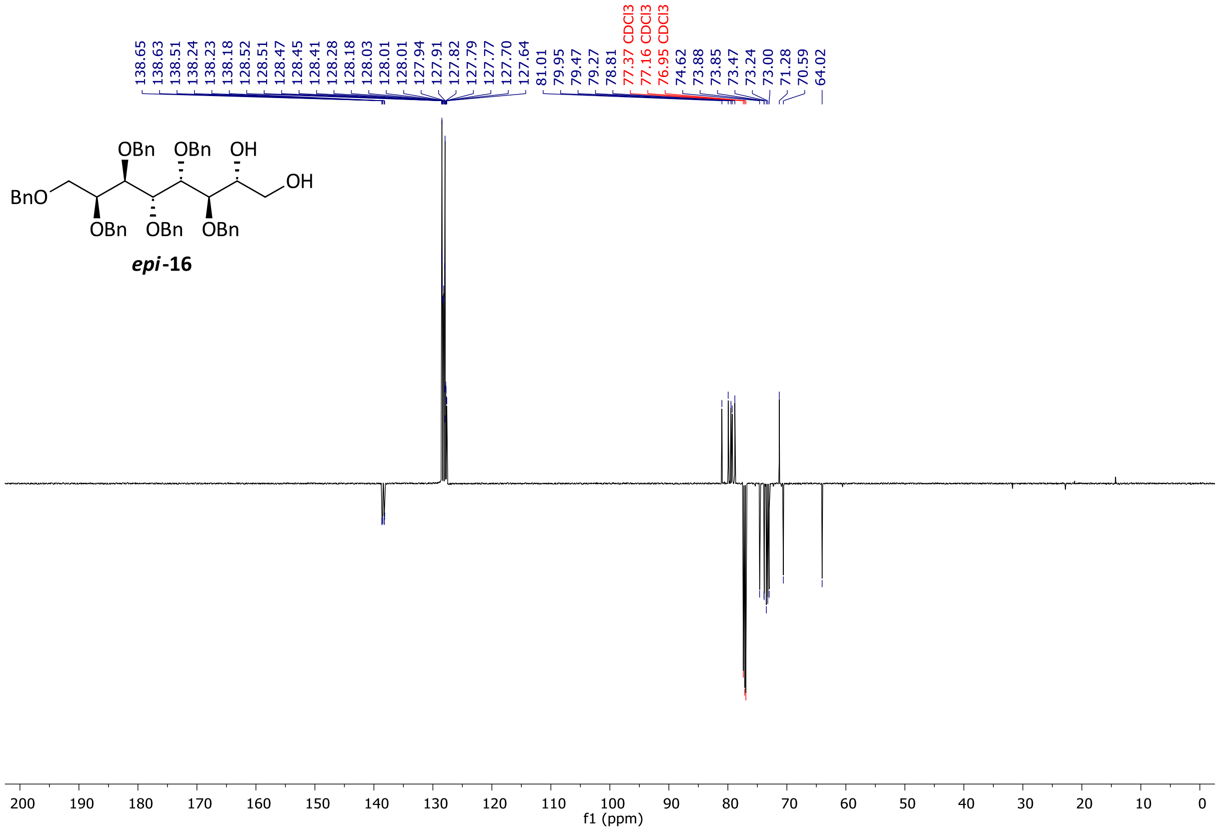

Figure S17. $^{13}C\{^{1}H\}$-NMR (151 MHz, $CDCl_3$) of 3,4,5,6,7,8-hexa-*O*-benzyl-L-*threo*-D-*talo*-octitol (***epi*-16**).

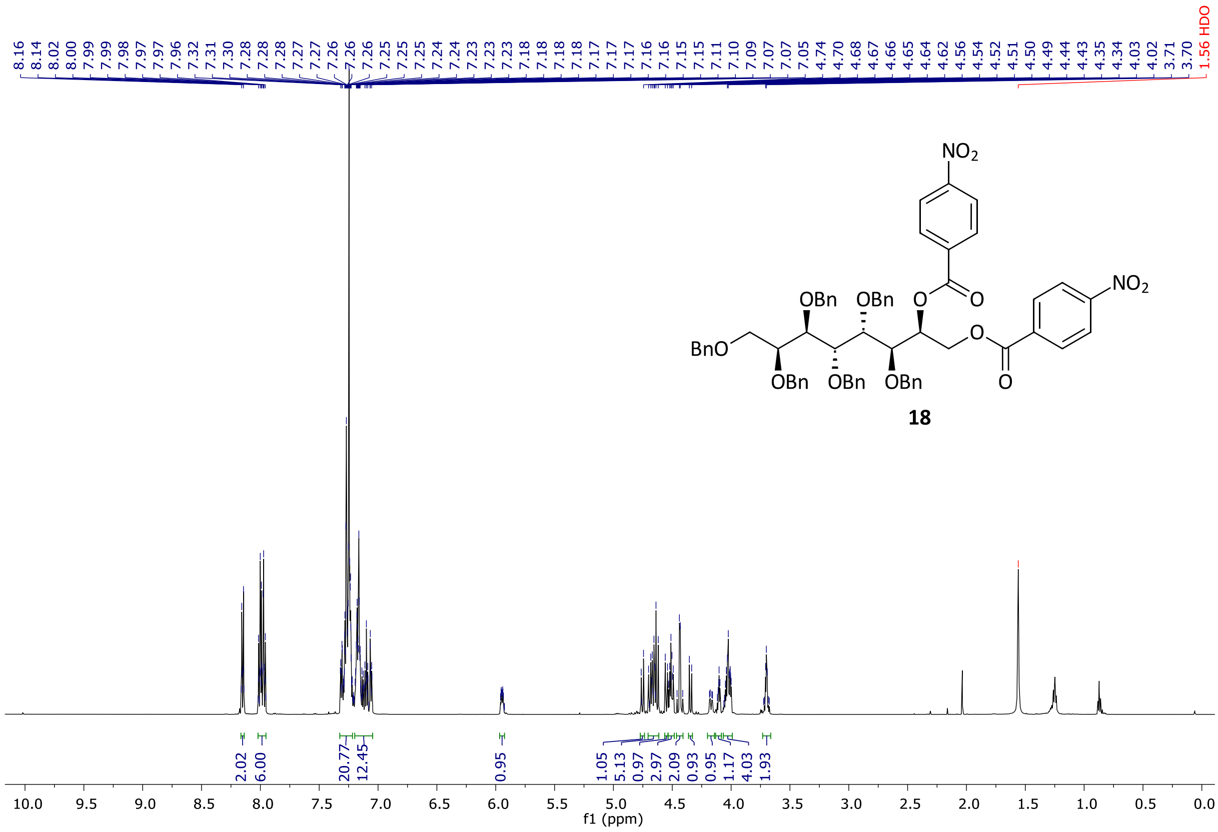

Figure S18. ^{1}H-NMR (600 MHz, $CDCl_3$) of 3,4,5,6,7,8-hexa-*O*-benzyl-1,2-di-*O*-(4-nitrobenzoyl)-L-*threo*-D-*galacto*-octitol (**18**).

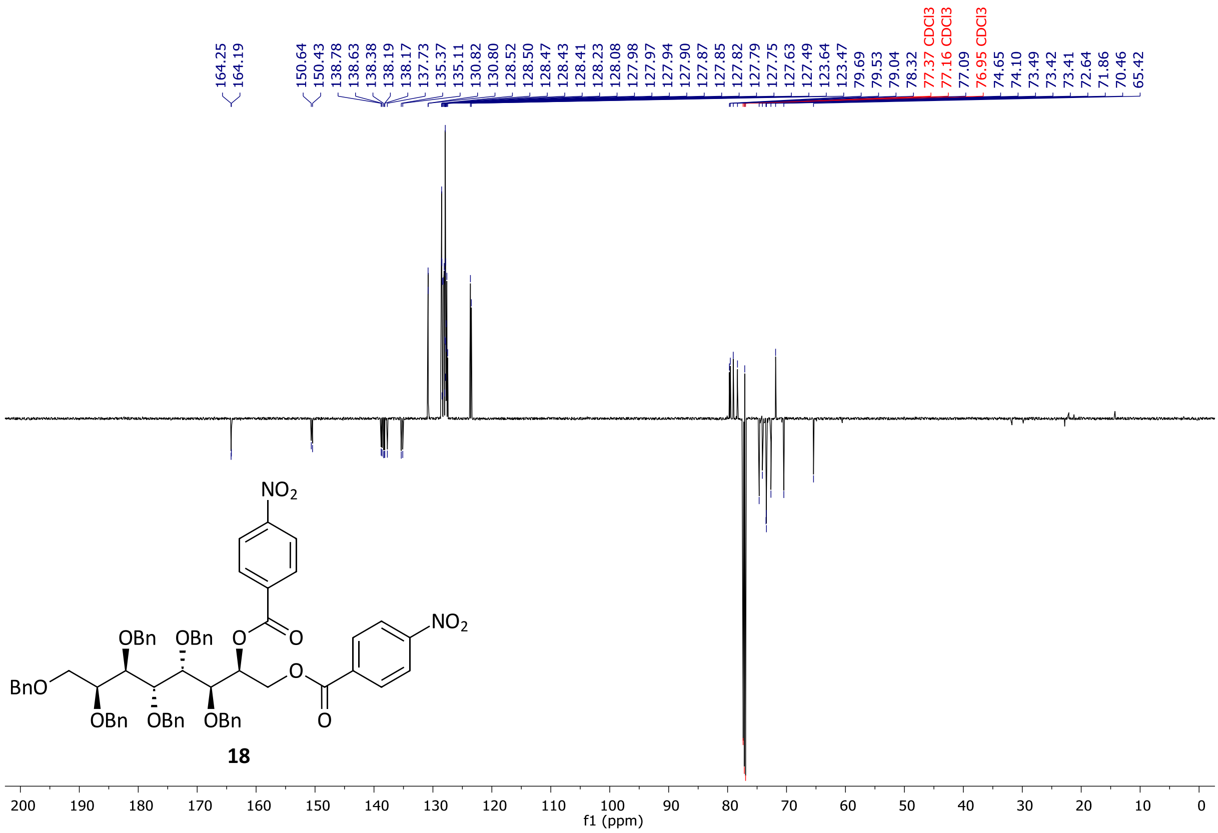

Figure S19. $^{13}C\{^{1}H\}$-NMR (151 MHz, $CDCl_3$) of 3,4,5,6,7,8-hexa-*O*-benzyl-1,2-di-*O*-(4-nitrobenzoyl)-L-*threo*-D-*galacto*-octitol (**18**).

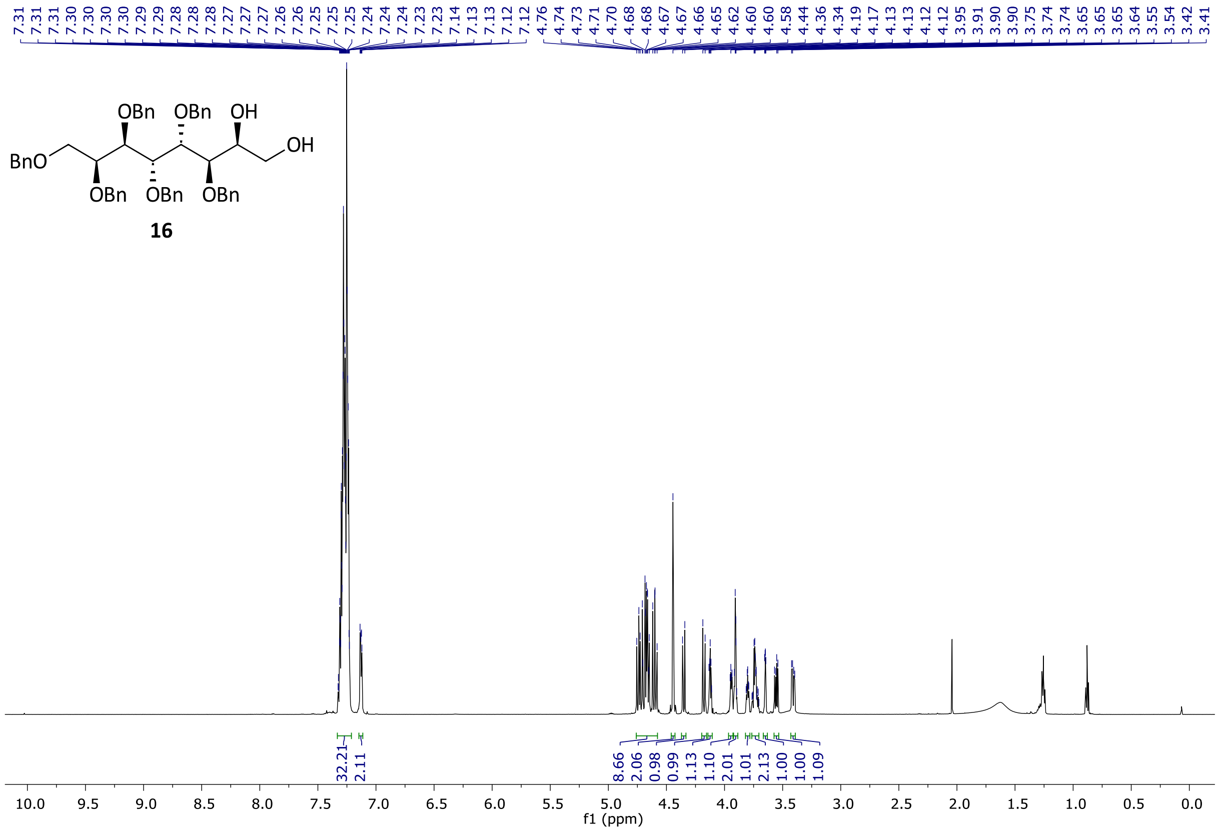

Figure S20. ^{1}H-NMR (600 MHz, $CDCl_3$) of 3,4,5,6,7,8-hexa-*O*-benzyl-L-*threo*-D-*galacto*-octitol (**16**).

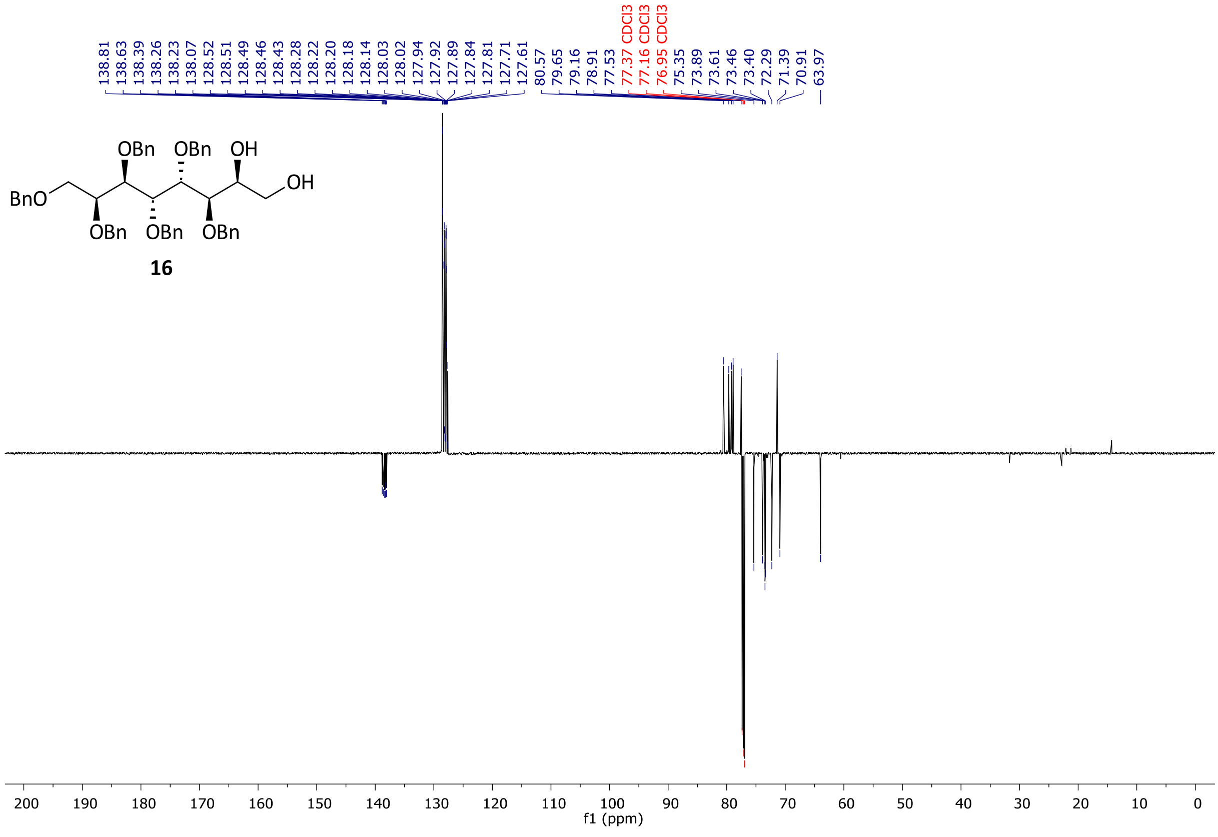

Figure S21. $^{13}C\{^{1}H\}$-NMR (151 MHz, $CDCl_3$) of 3,4,5,6,7,8-hexa-*O*-benzyl-L-*threo*-D-*galacto*-octitol (**16**).

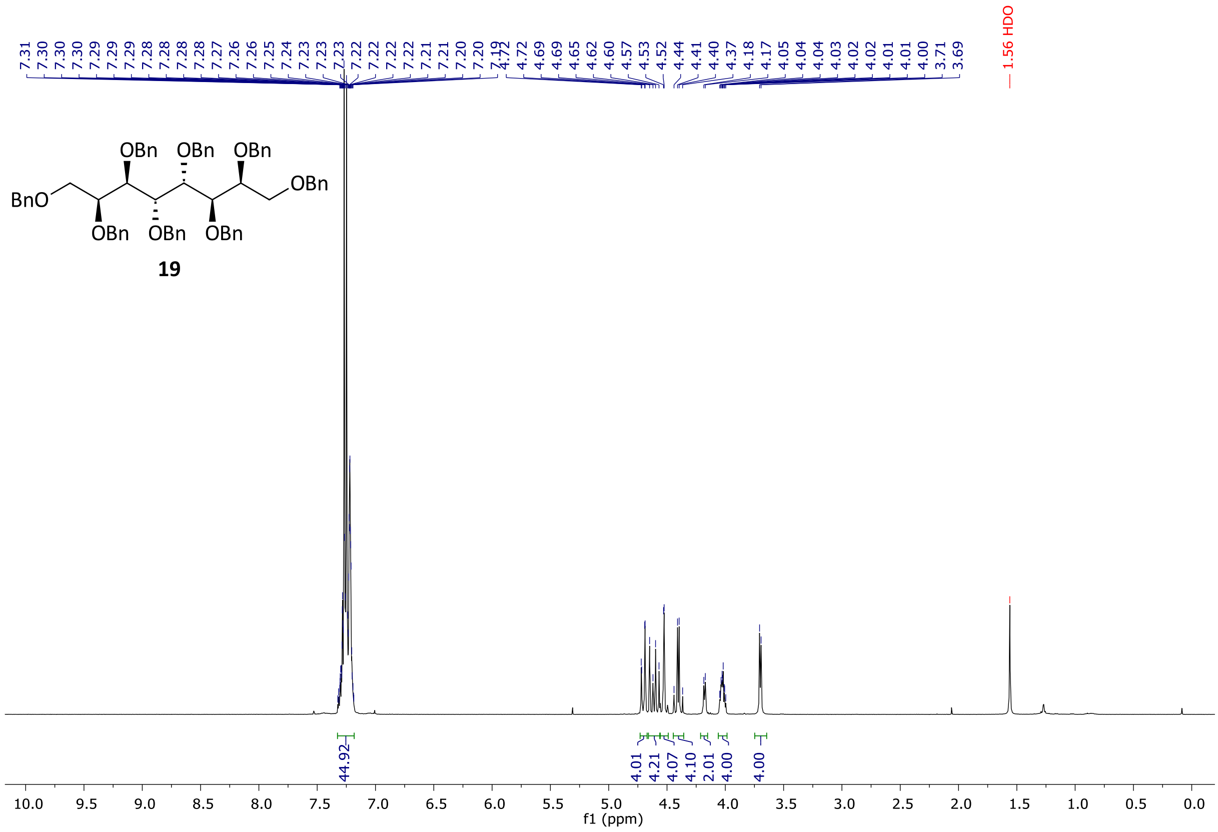

Figure S22. ^{1}H-NMR (400 MHz, $CDCl_3$) of 1,2,3,4,5,6,7,8-octa-*O*-benzyl-L-*threo*-D-*galacto*-octitol (**19**).

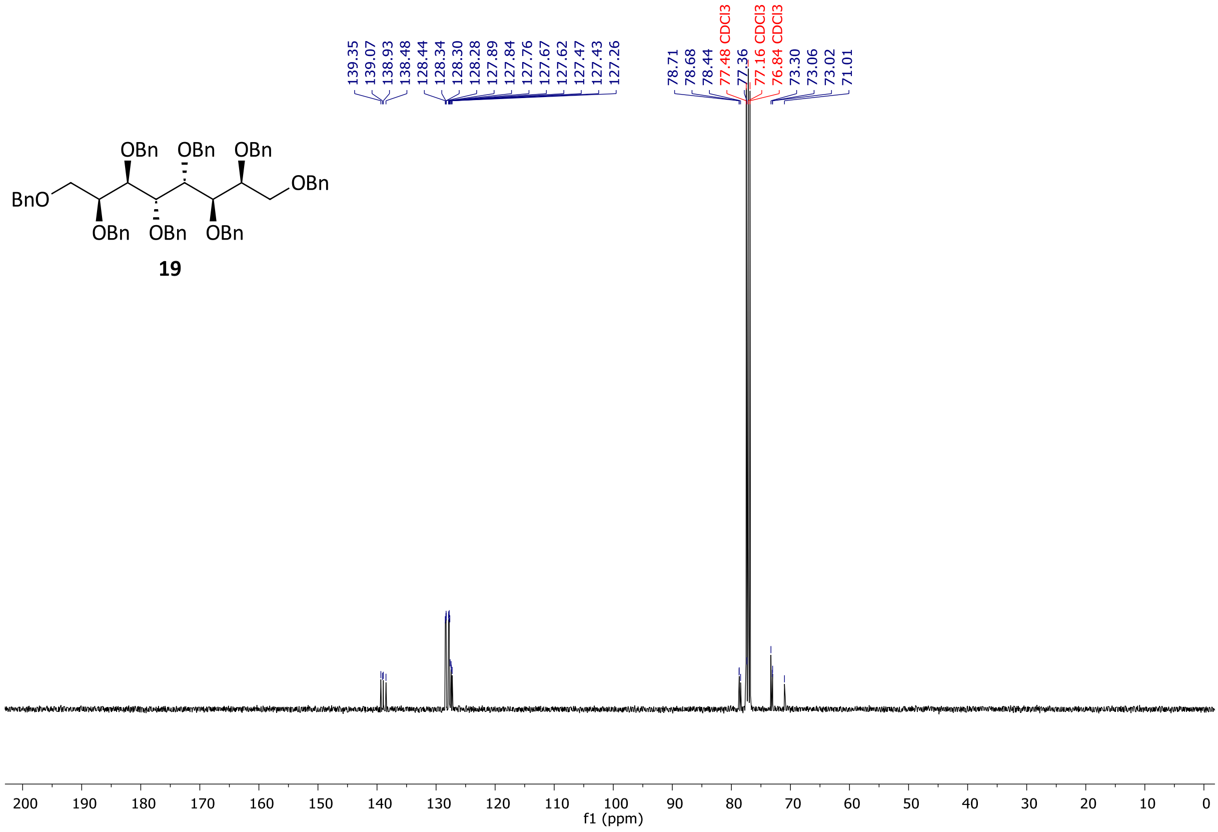

Figure S23. $^{13}C\{^{1}H\}$-NMR (101 MHz, $CDCl_3$) of 1,2,3,4,5,6,7,8-octa-*O*-benzyl-L-*threo*-D-*galacto*-octitol (**19**).

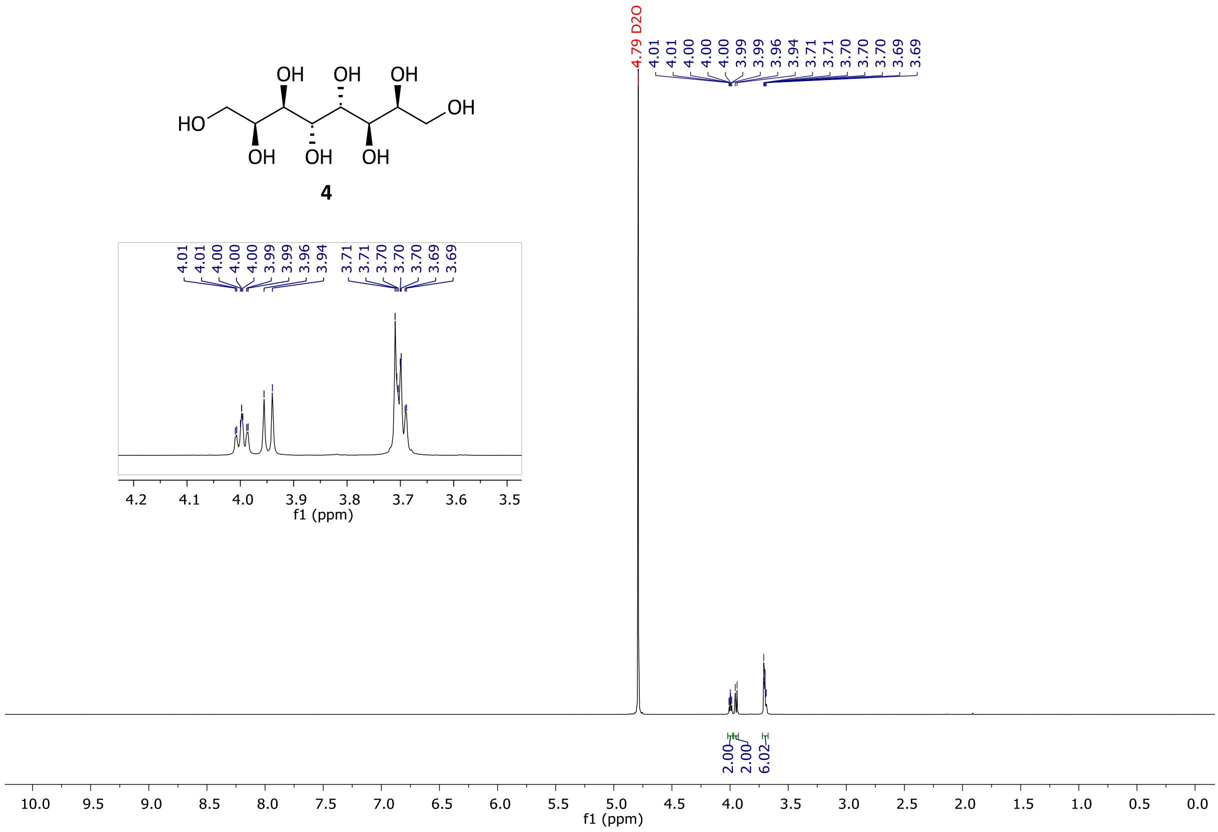

Figure S24. ^{1}H-NMR (600 MHz, D_2O) of L-*threo*-D-*galacto*-octitol (**4**).

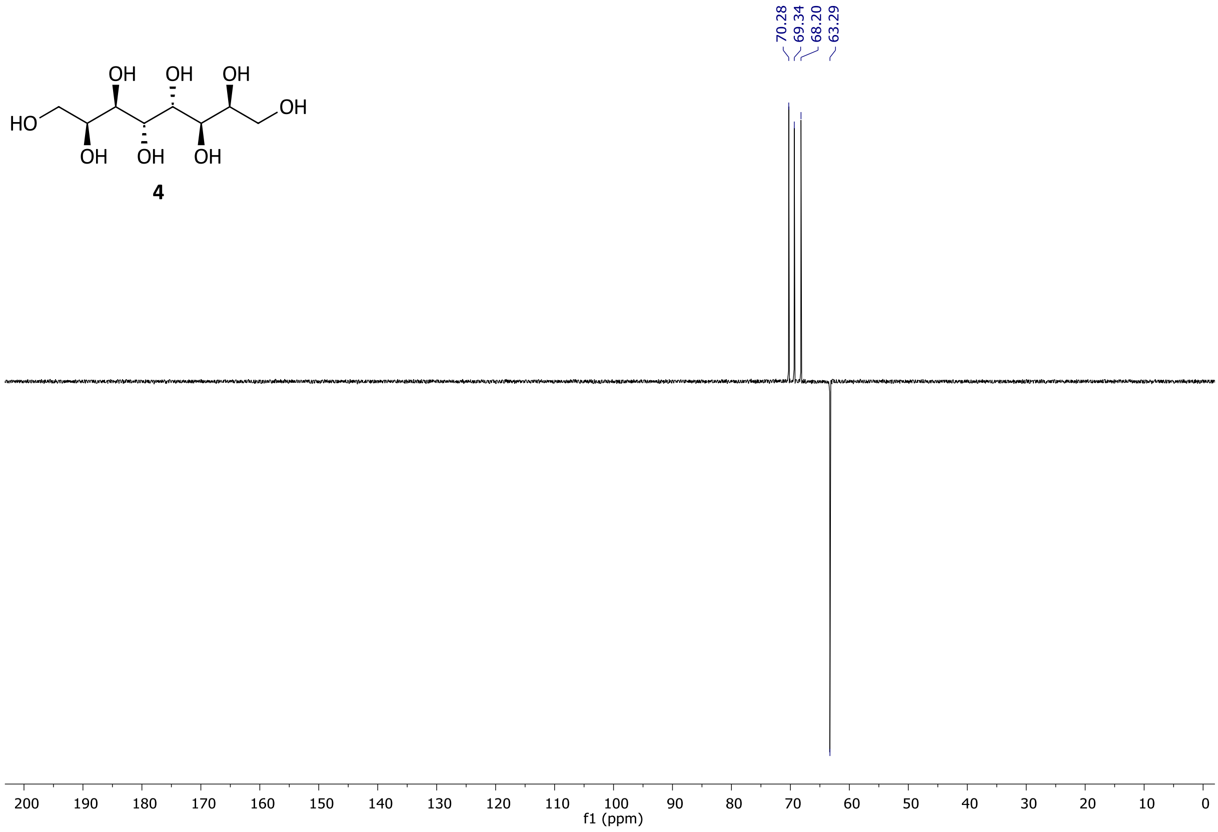

Figure S25. $^{13}C\{^{1}H\}$-NMR (151 MHz, D_2O) of L-*threo*-D-*galacto*-octitol (**4**).

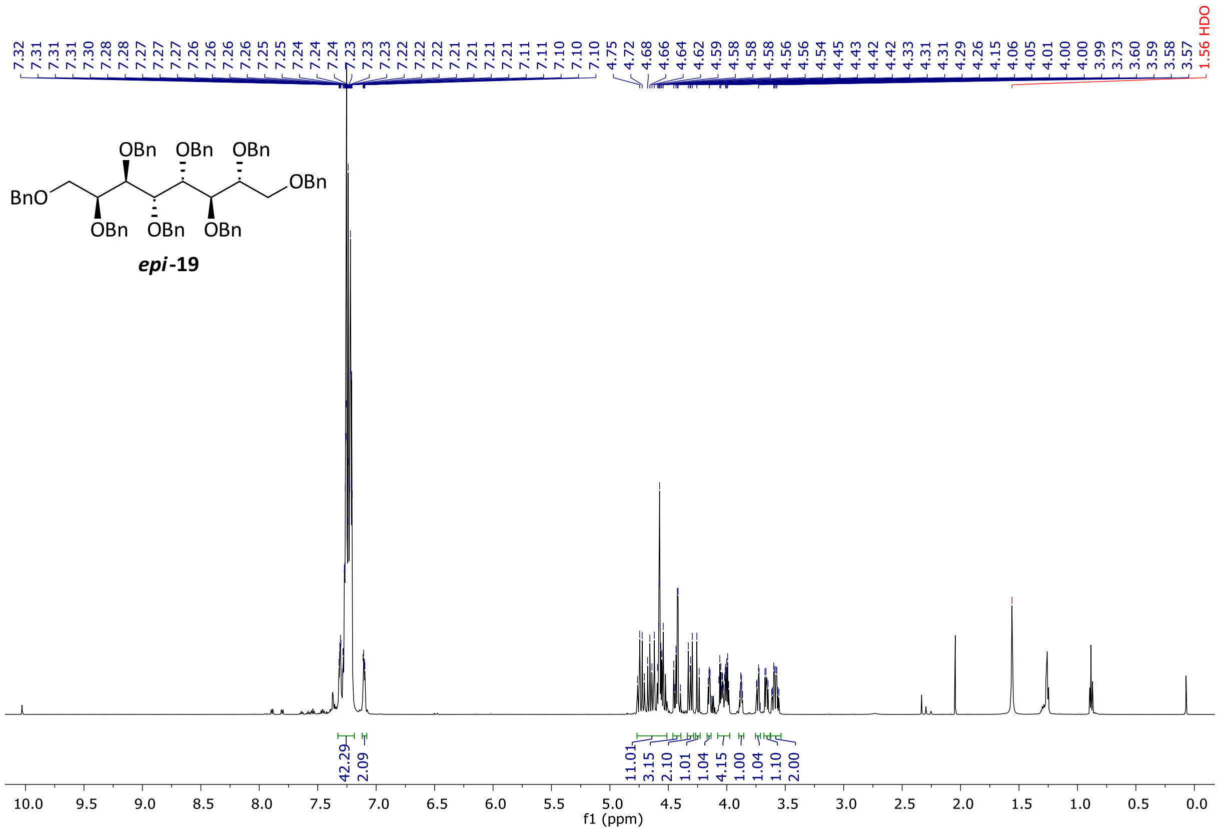

Figure S26. ^{1}H-NMR (600 MHz, $CDCl_3$) of 1,2,3,4,5,6,7,8-octa-*O*-benzyl-L-*threo*-D-*talo*-octitol (***epi*-19**).

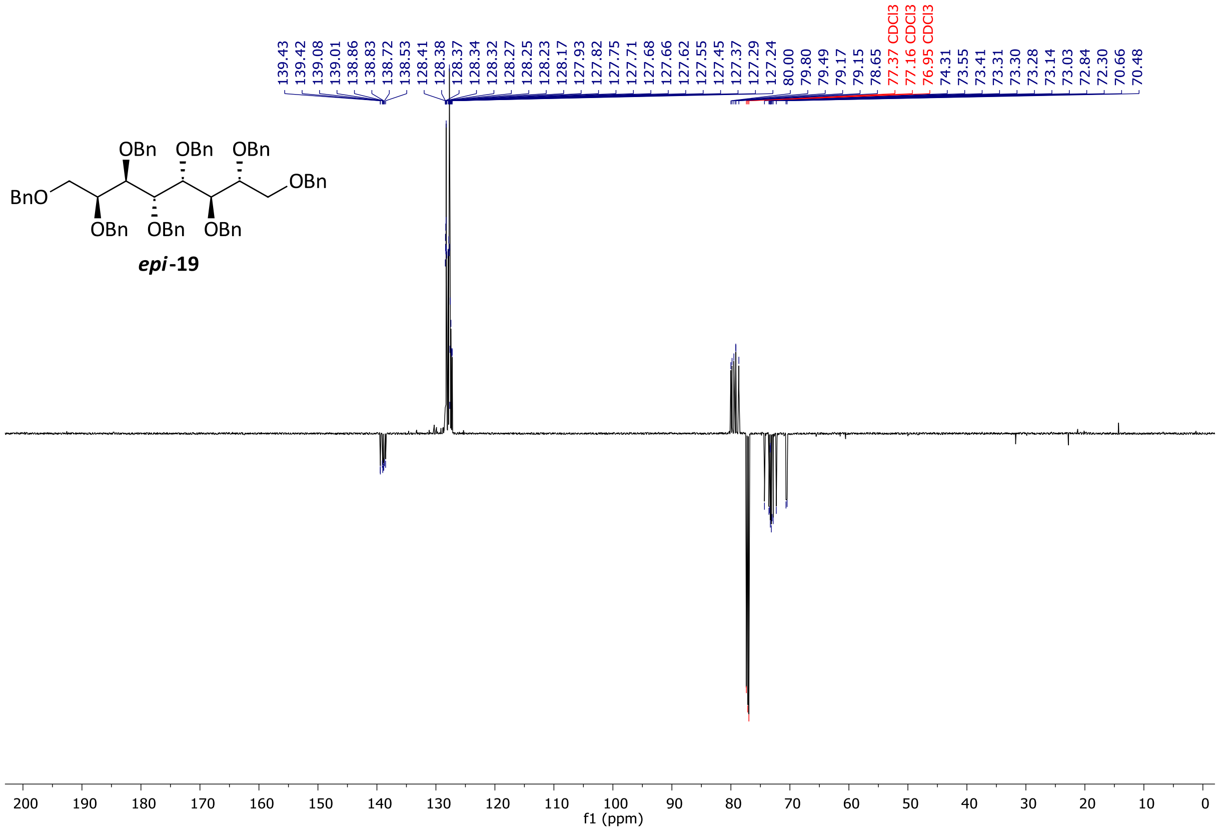

Figure S27. $^{13}C\{^{1}H\}$-NMR (151 MHz, $CDCl_3$) of 1,2,3,4,5,6,7,8-octa-*O*-benzyl-L-*threo*-D-*talo*-octitol (***epi*-19**).

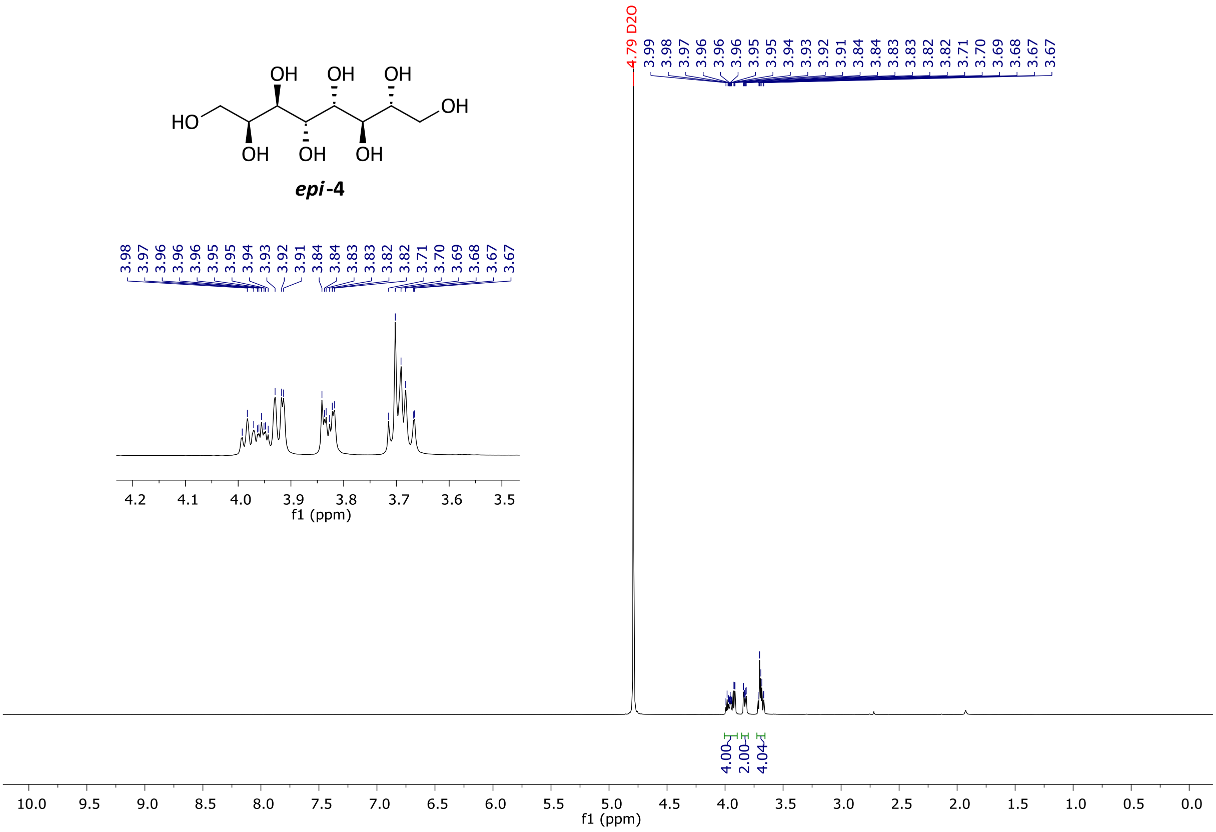

Figure S28. ^{1}H-NMR (600 MHz, D_2O) of L-*threo*-D-*talo*-octitol (***epi*-4**).

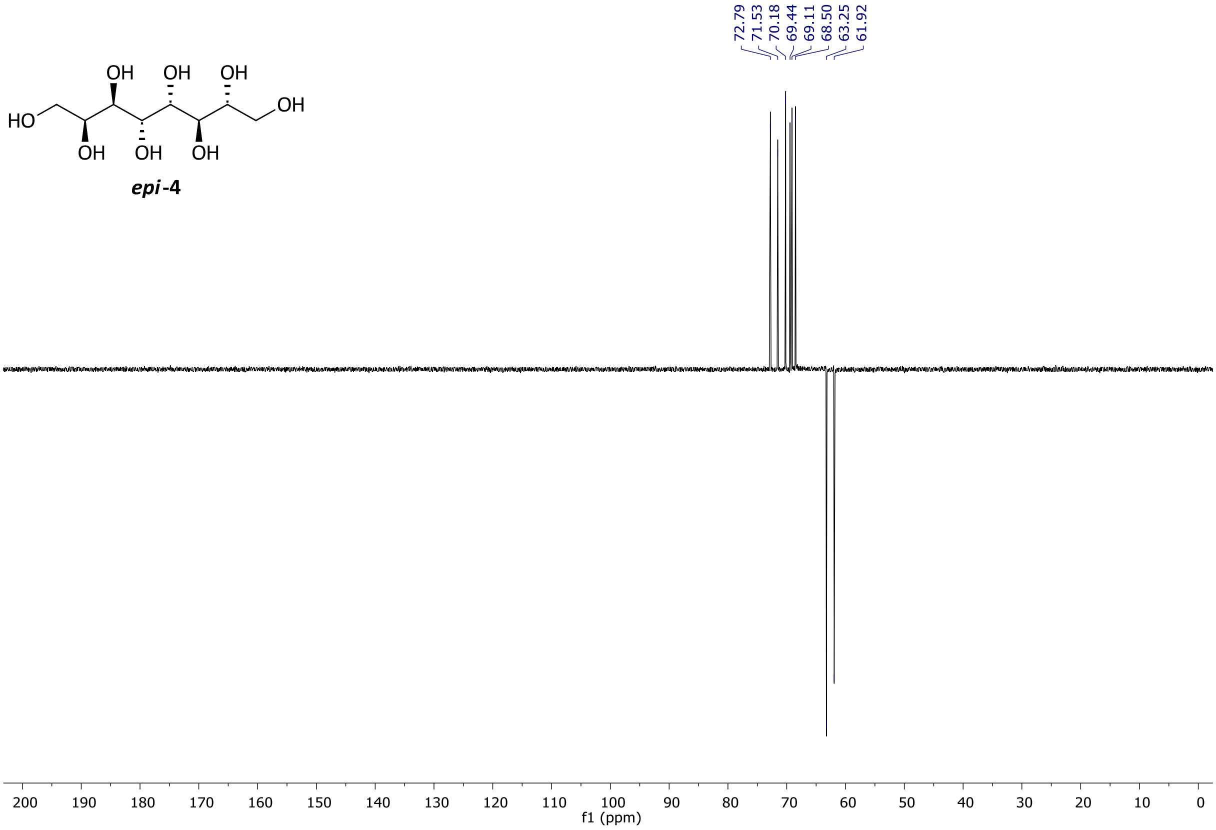

Figure S29. $^{13}C\{^{1}H\}$-NMR (151 MHz, D_2O) of L-*threo*-D-*talo*-octitol (***epi*-4**).

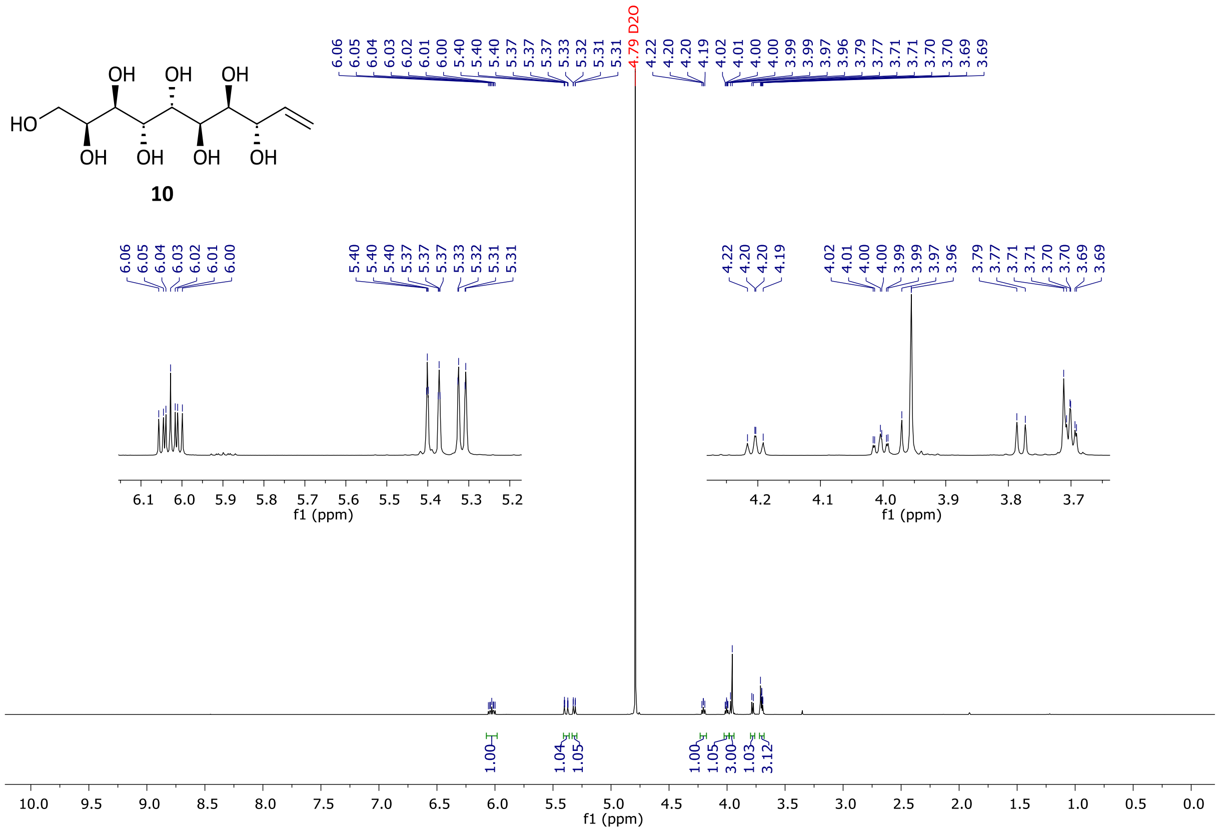

Figure S30. ^{1}H-NMR (600 MHz, D_2O) of 1,2-dideoxy-L-*lyxo*-L-*manno*-dec-1-enitol (**10**).

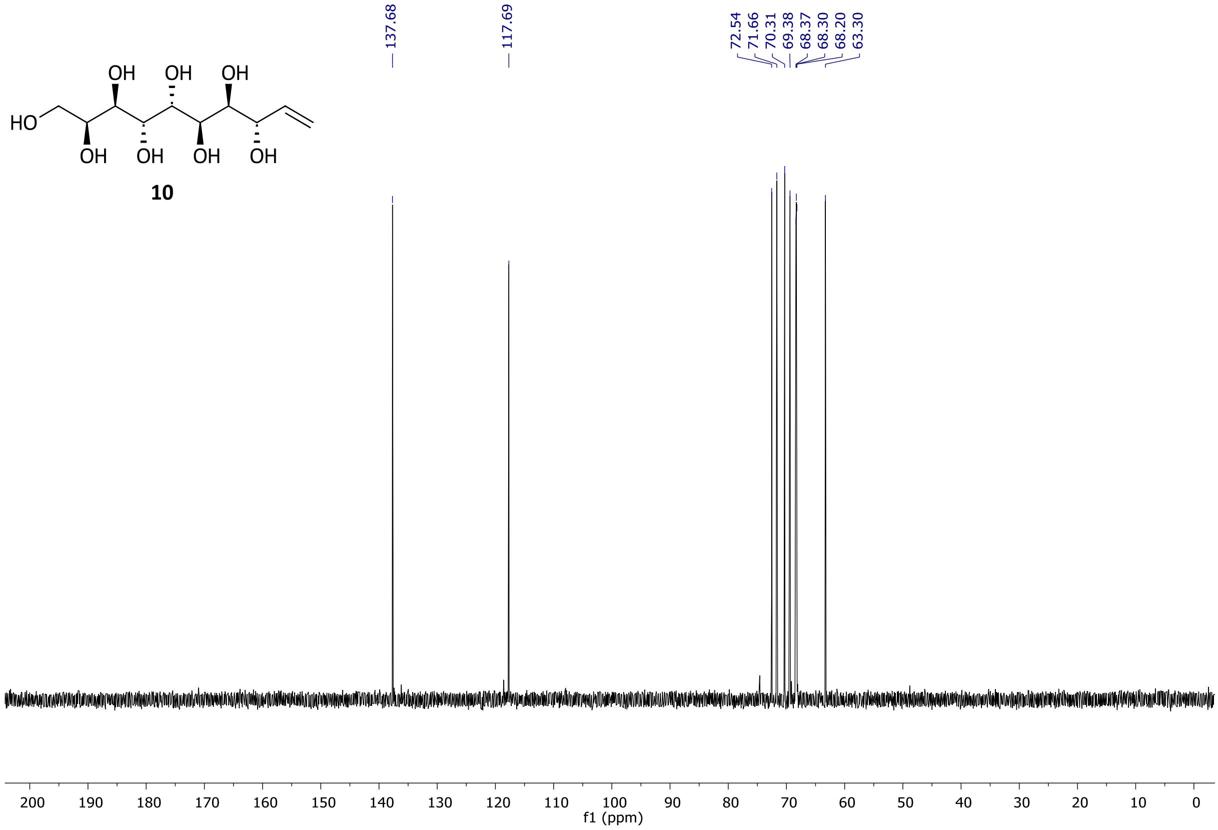

Figure S31. $^{13}C\{^{1}H\}$-NMR (151 MHz, D_2O) of 1,2-dideoxy-L-*lyxo*-L-*manno*-dec-1-enitol (**10**).

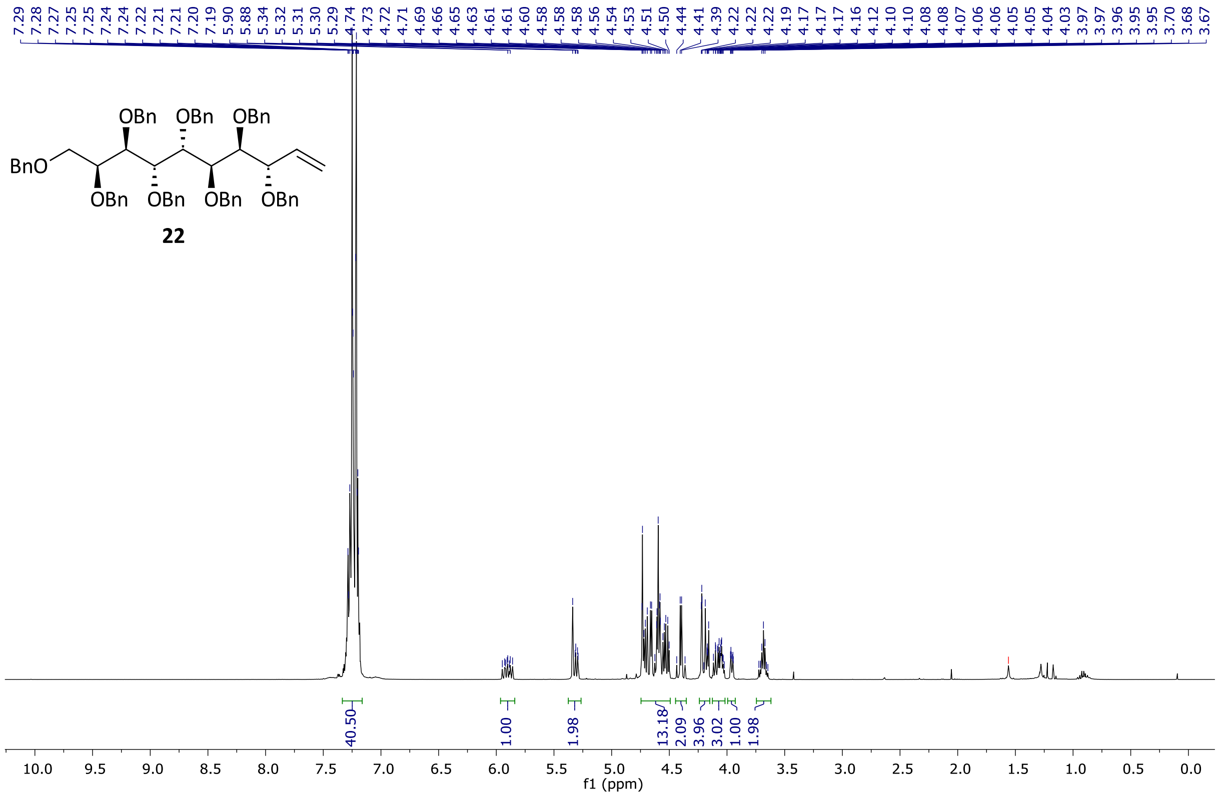

Figure S32. ^{1}H-NMR (400 MHz, $CDCl_3$) of 3,4,5,6,7,8,9,10-octa-*O*-benzyl-1,2-dideoxy-L-*lyxo*-L-*manno*-dec-1-enitol (**22**).

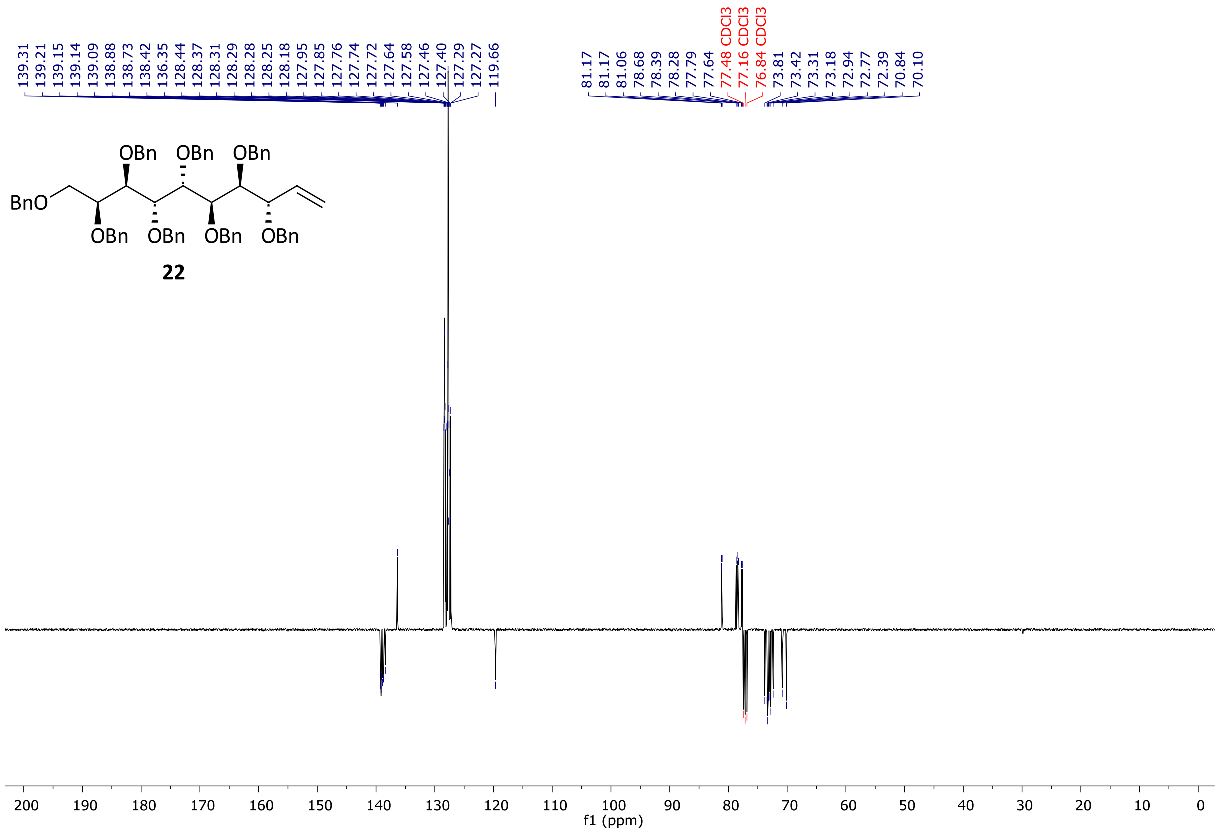

Figure S33. $^{13}C\{^{1}H\}$-NMR (101 MHz, $CDCl_3$) of 3,4,5,6,7,8,9,10-octa-*O*-benzyl-1,2-dideoxy-L-*lyxo*-L-*manno*-dec-1-enitol (**22**).

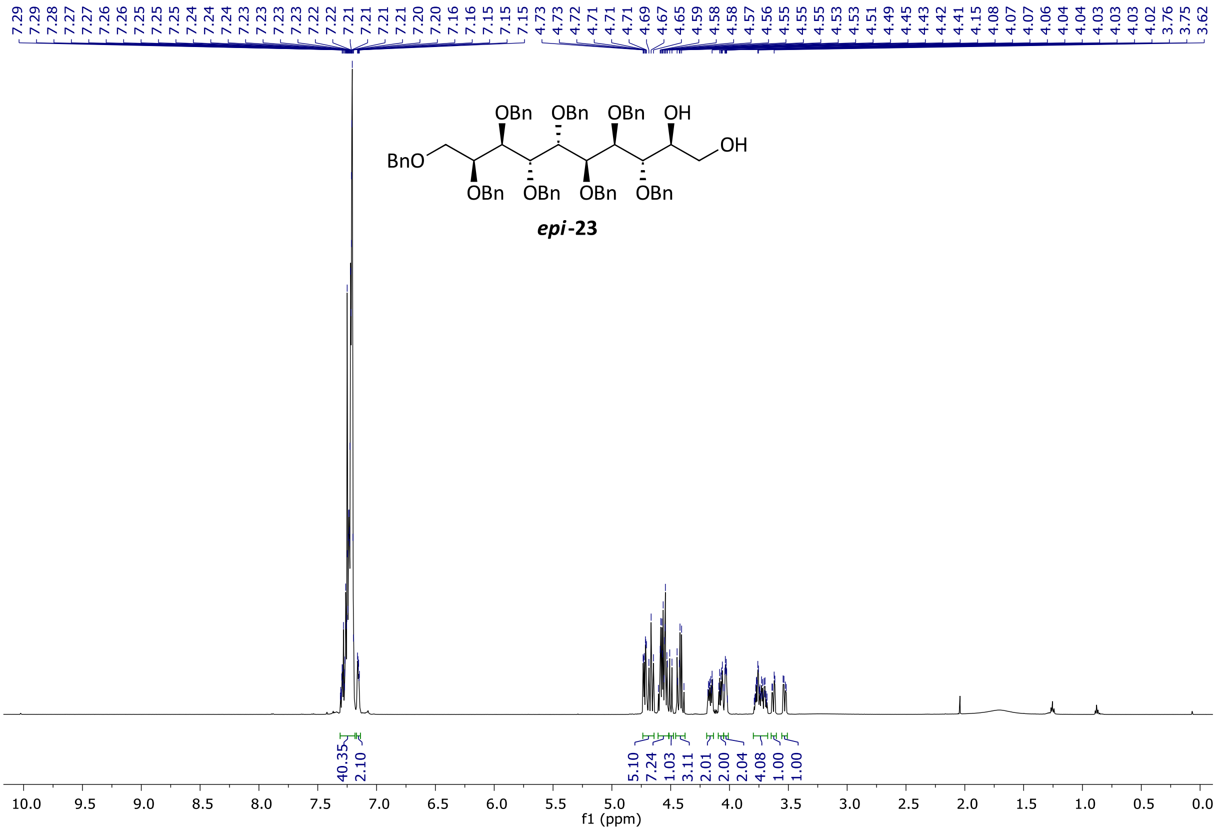

Figure S34. ^{1}H-NMR (600 MHz, $CDCl_3$) of 3,4,5,6,7,8,9,10-octa-*O*-benzyl-L-*galacto*-L-*talo*-decitol (***epi*-23**).

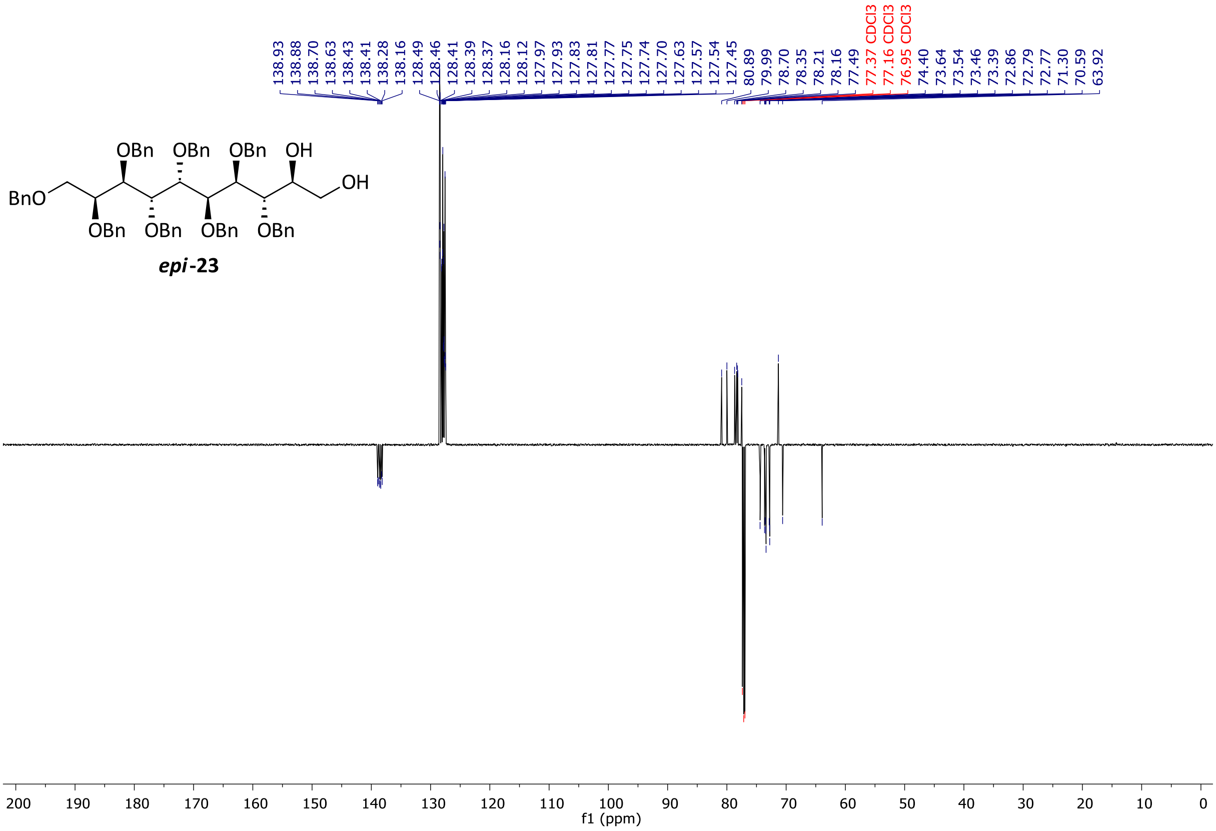

Figure S35. $^{13}C\{^{1}H\}$-NMR (151 MHz, $CDCl_3$) of 3,4,5,6,7,8,9,10-octa-*O*-benzyl-L-*galacto*-L-*talo*-decitol (***epi*-23**).

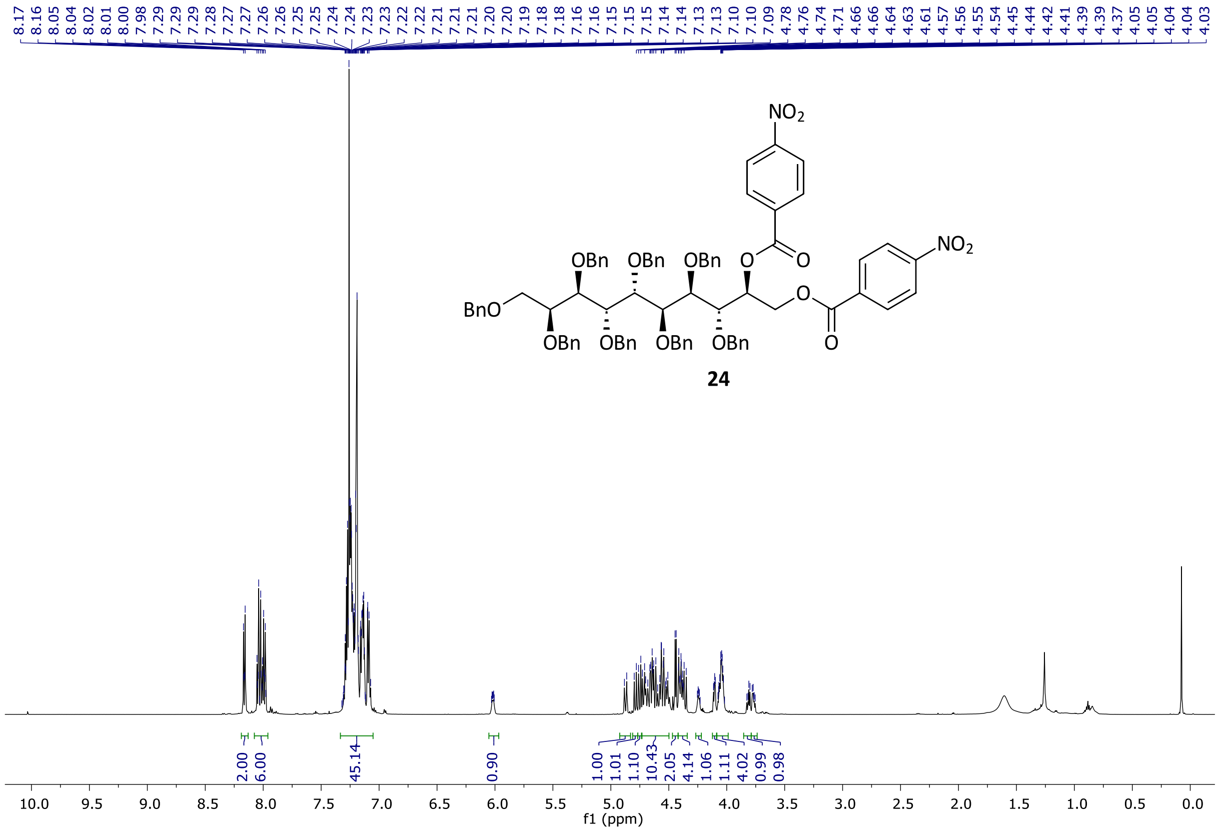

Figure S36. ^{1}H-NMR (600 MHz, $CDCl_3$) of 3,4,5,6,7,8,9,10-octa-*O*-benzyl-1,2-di-*O*-(4-nitrobenzoyl)-L-*galacto*-L-*galacto*-decitol (**24**).

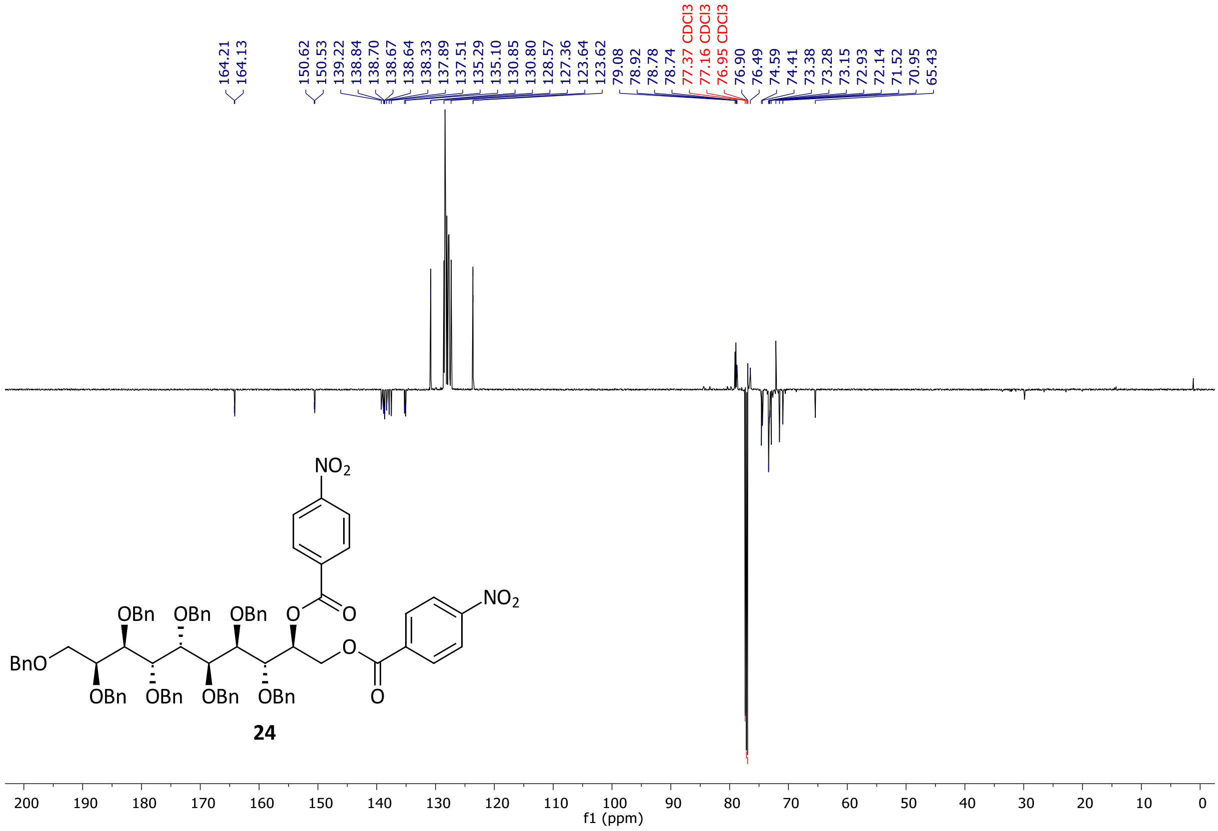

Figure S37. $^{13}C\{^{1}H\}$-NMR (151 MHz, $CDCl_3$) of 3,4,5,6,7,8,9,10-octa-*O*-benzyl-1,2-di-*O*-(4-nitrobenzoyl)-L-*galacto*-L-*galacto*-decitol (**24**).

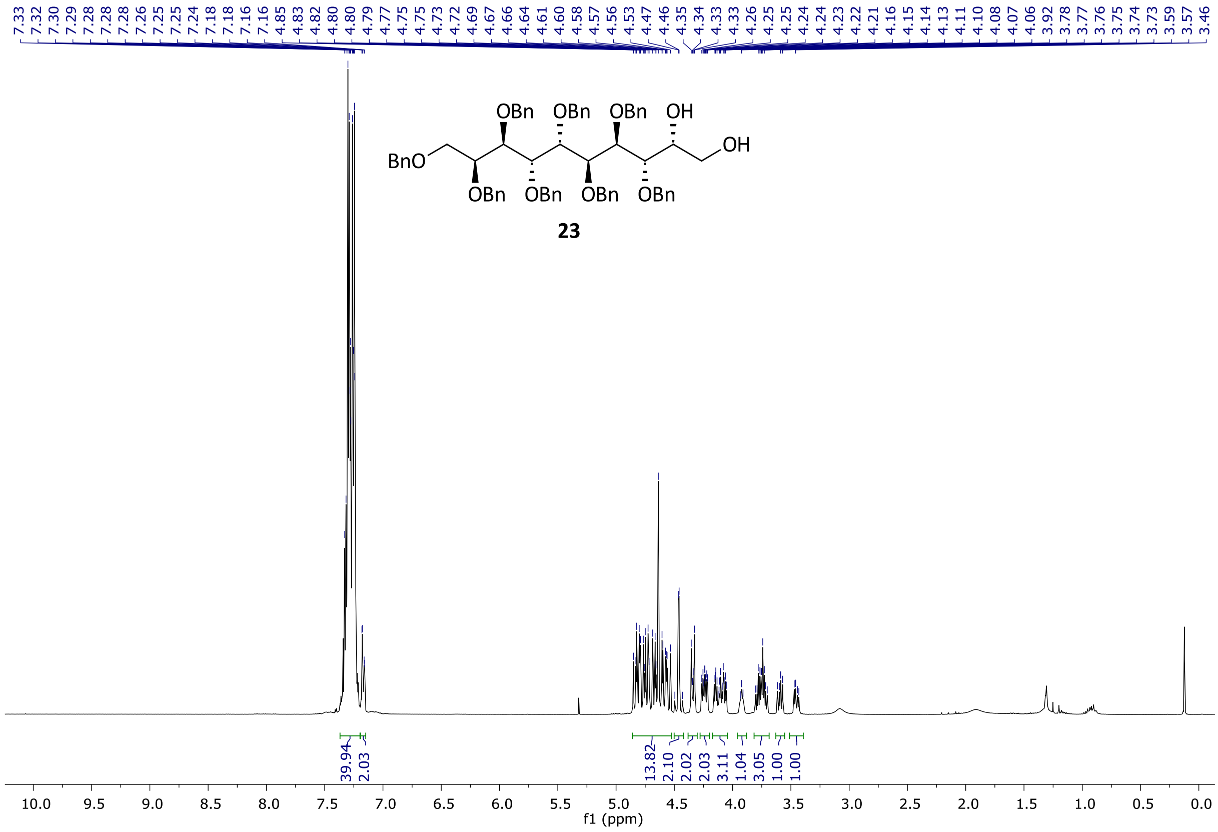

Figure S38. ^{1}H-NMR (600 MHz, $CDCl_3$) of 3,4,5,6,7,8,9,10-octa-*O*-benzyl-L-*galacto*-L-*galacto*-decitol (**23**).

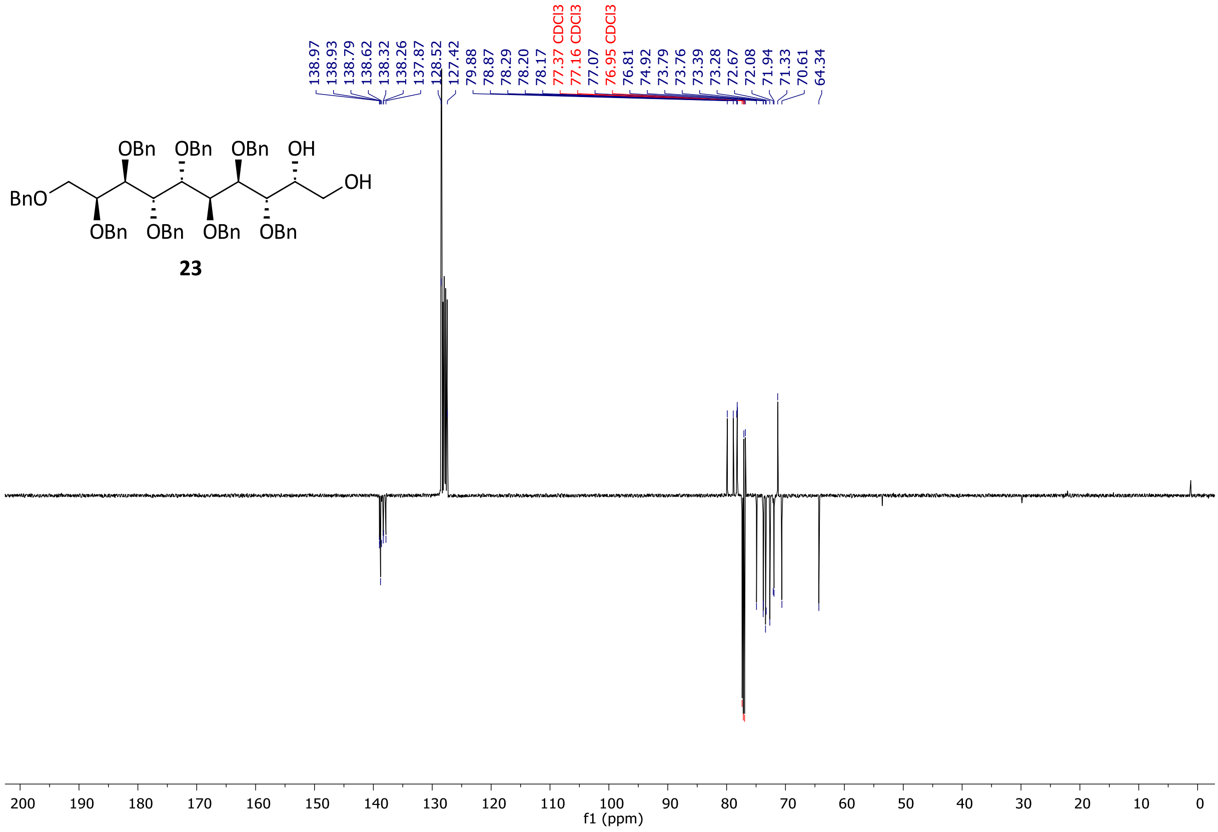

Figure S39. $^{13}C\{^{1}H\}$-NMR (151 MHz, $CDCl_3$) of 3,4,5,6,7,8,9,10-octa-*O*-benzyl-L-*galacto*-L-*galacto*-decitol (**23**).

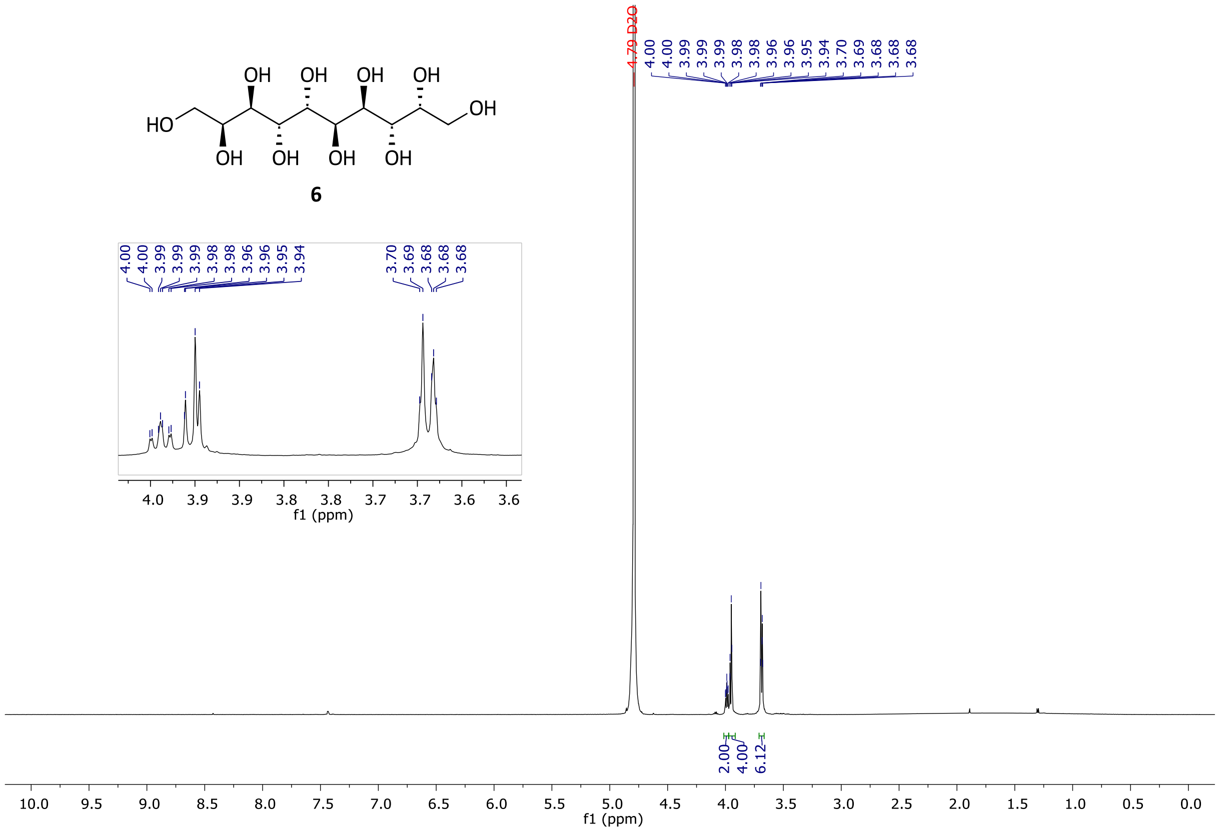

Figure S40. ^{1}H-NMR (600 MHz, D_2O) of *meso*-L-*galacto*-L-*galacto*-decitol (**6**).

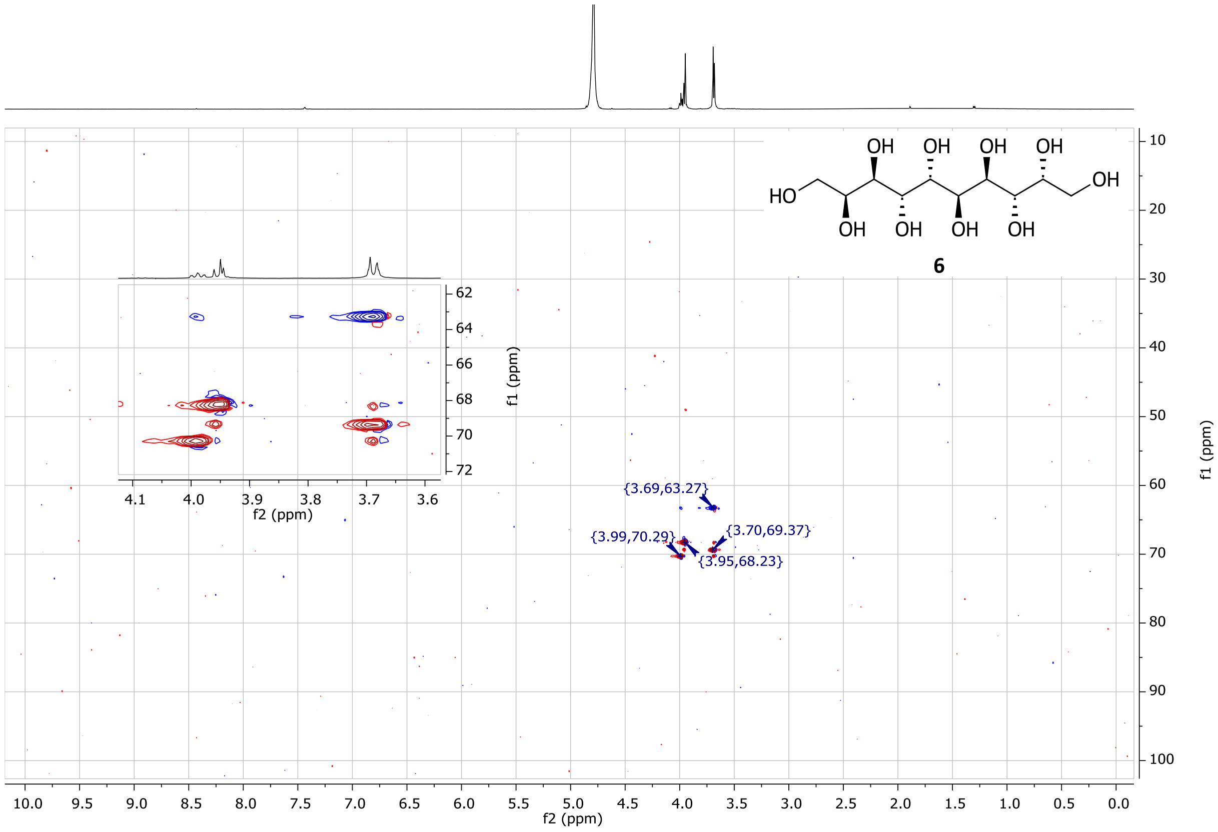

Figure S41. ^{1}H,^{13}C{^{1}H}-HSQC (151 MHz, D_2O) of *meso*-L-*galacto*-L-*galacto*-decitol (**6**).

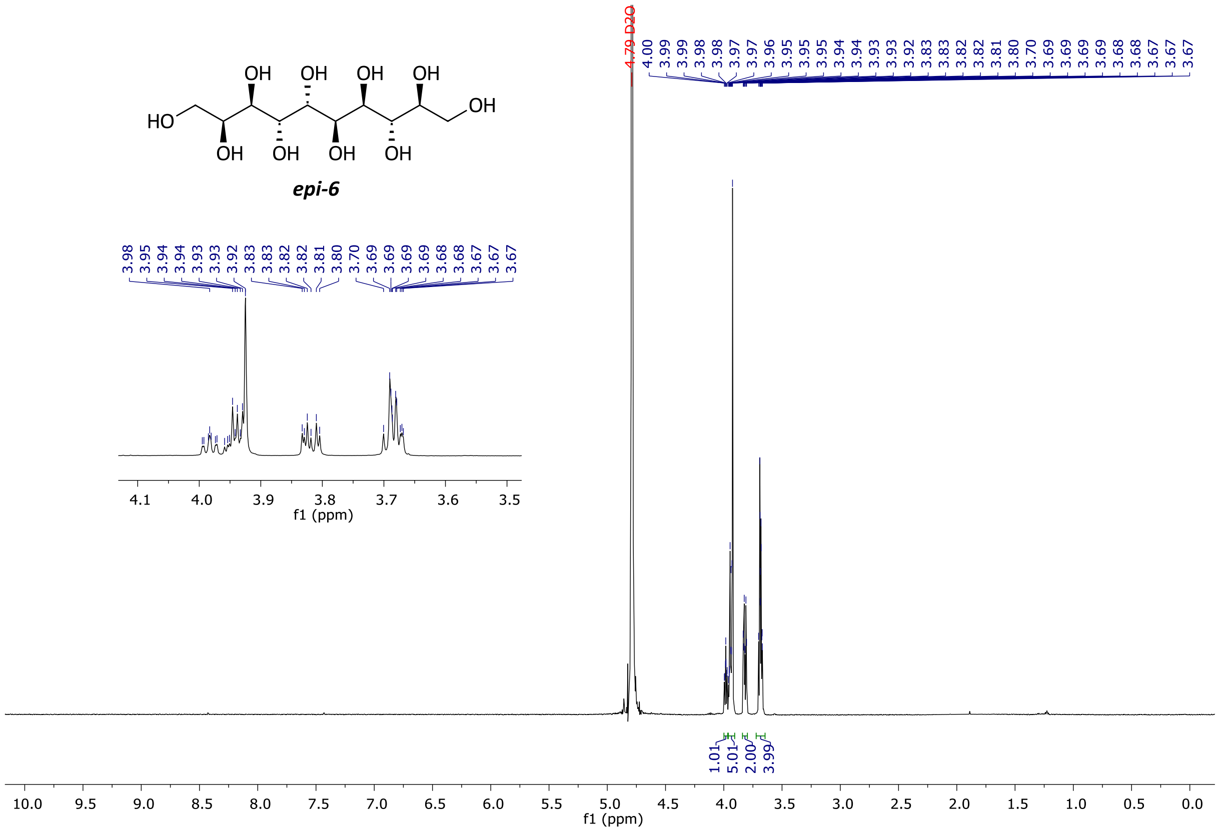

Figure S42. ^{1}H-NMR (600 MHz, D_2O) of L-*galacto*-L-*talo*-decitol (***epi*-6**).

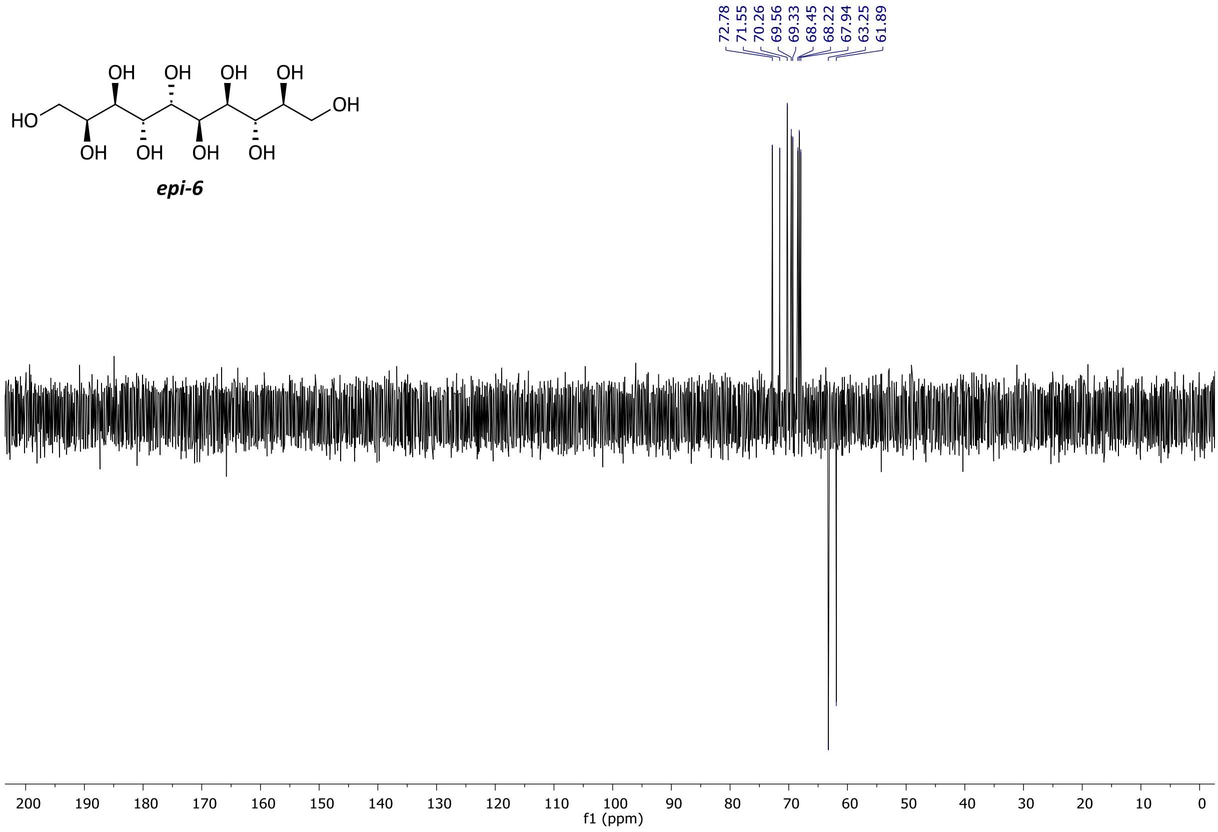

Figure S43. $^{13}C\{^{1}H\}$-NMR (151 MHz, D_2O) of L-*galacto*-L-*talo*-decitol (***epi*-6**).

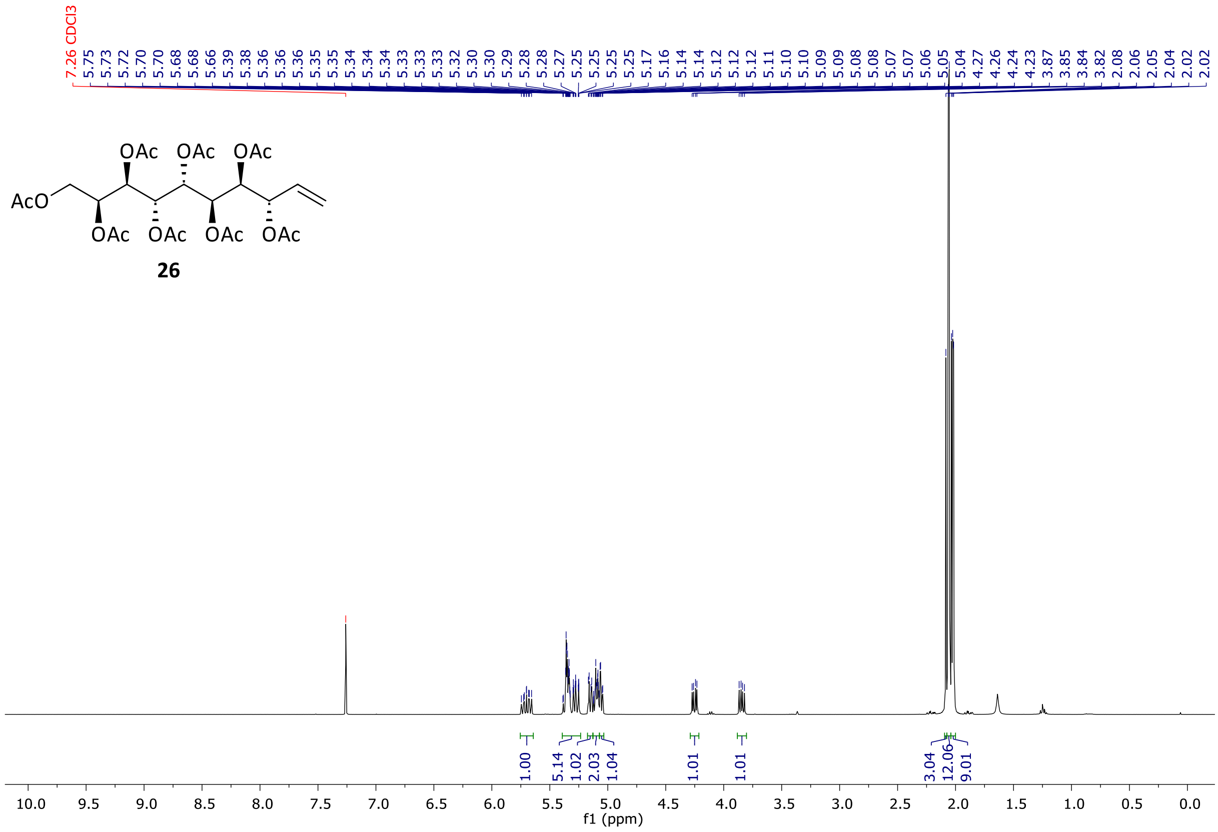

Figure S44. ^{1}H-NMR (400 MHz, $CDCl_3$) of 3,4,5,6,7,8,9,10-Octa-*O*-acetyl-1,2-dideoxy-L-*lyxo*-L-*manno*-dec-1-enitol (**25**).

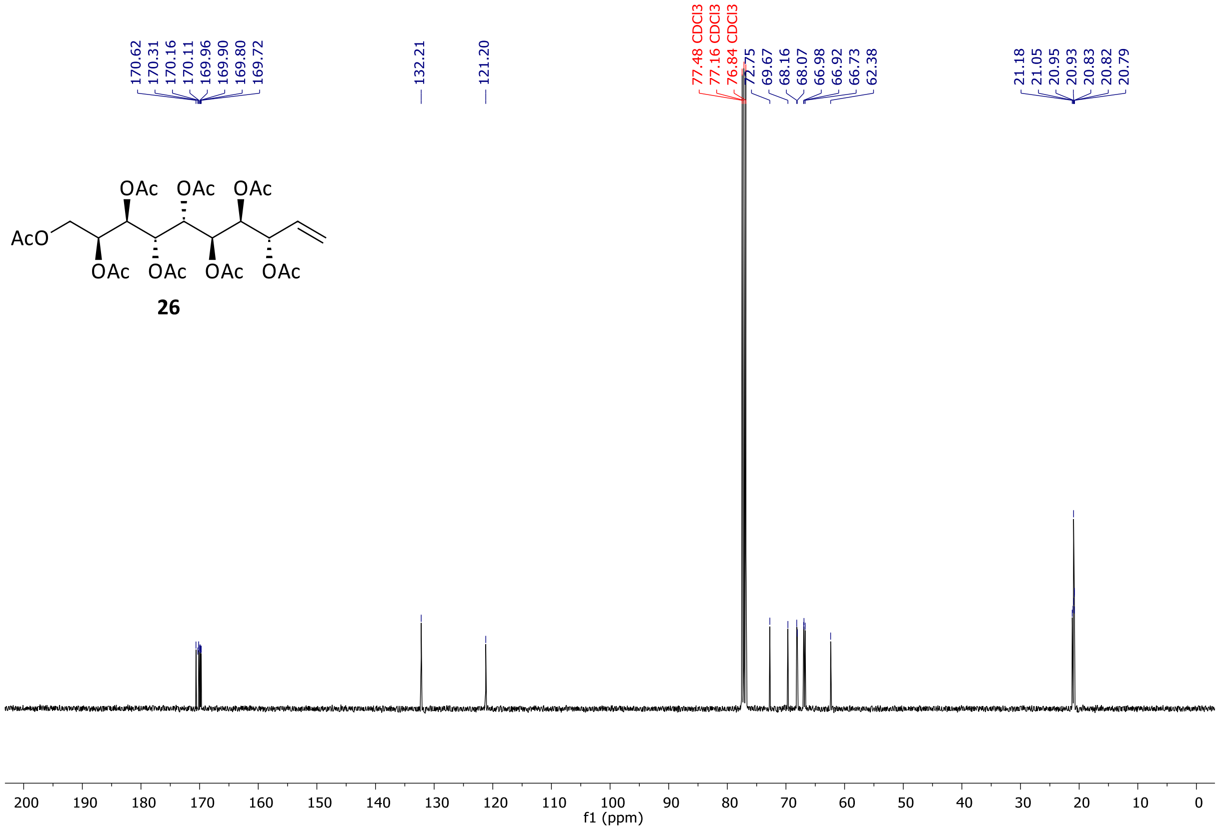

Figure S45. $^{13}C\{^{1}H\}$-NMR (101 MHz, $CDCl_3$) of 3,4,5,6,7,8,9,10-Octa-*O*-acetyl-1,2-dideoxy-L-*lyxo*-L-*manno*-dec-1-enitol (**25**).

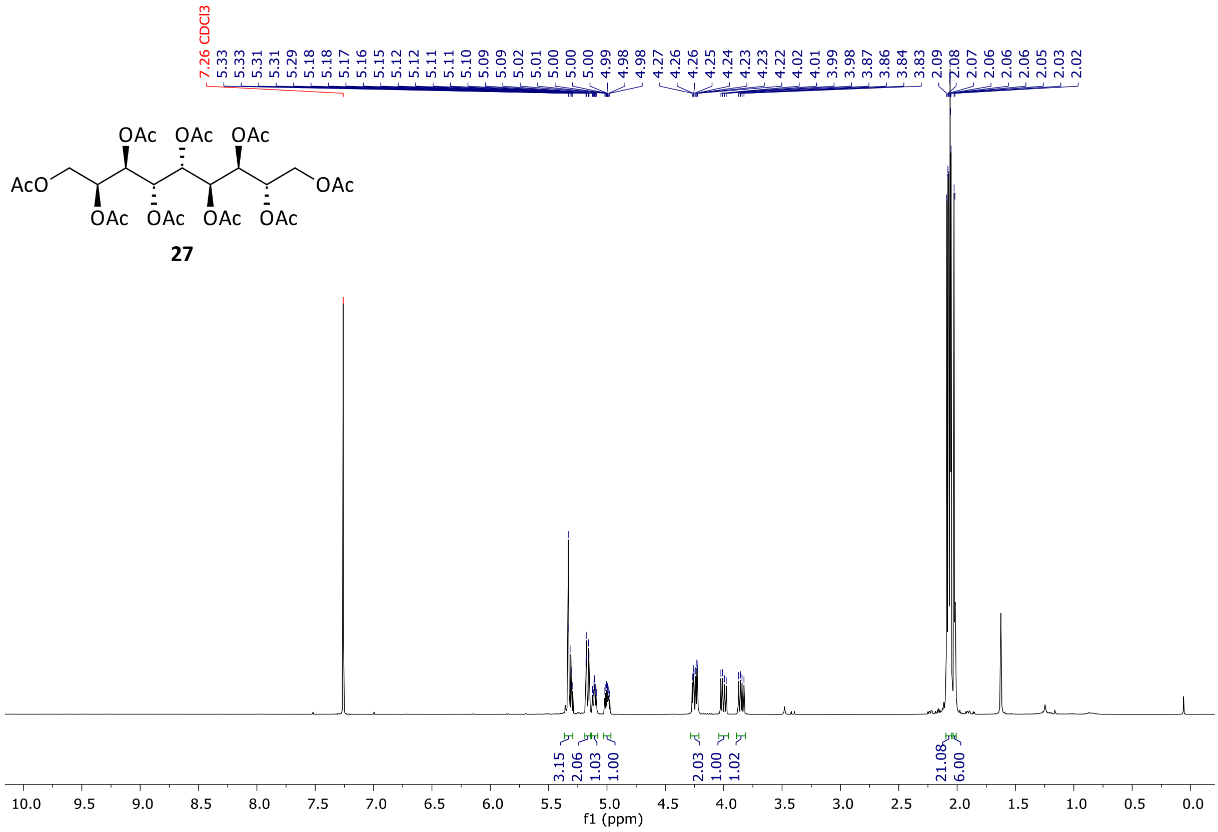

Figure S46. ^{1}H-NMR (400 MHz, $CDCl_3$) of 1,2,3,4,5,6,7,8,9-nona-*O*-acetyl-L-*lyxo*-L-*manno*-nonitol (**26**).

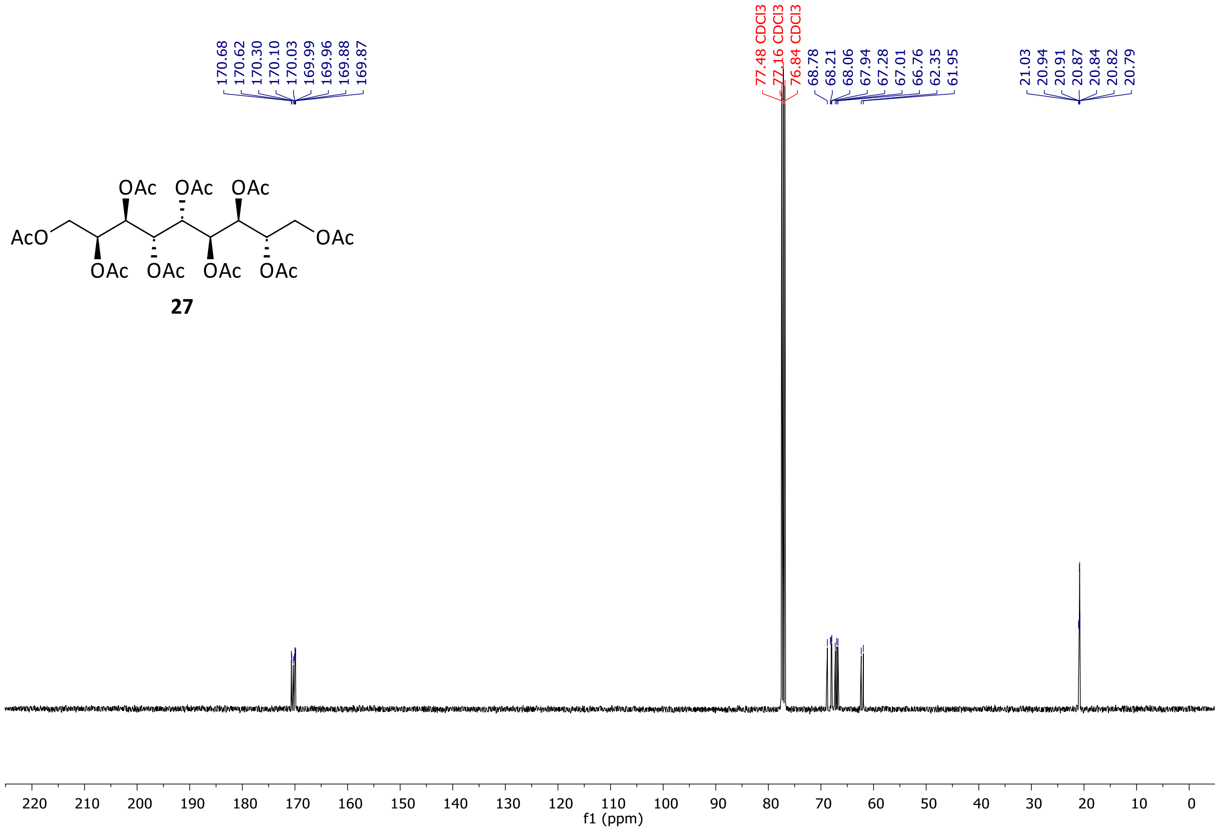

Figure S47. $^{13}C\{^{1}H\}$-NMR (101 MHz, $CDCl_3$) of 1,2,3,4,5,6,7,8,9-nona-*O*-acetyl-L-*lyxo*-L-*manno*-nonitol (**26**).

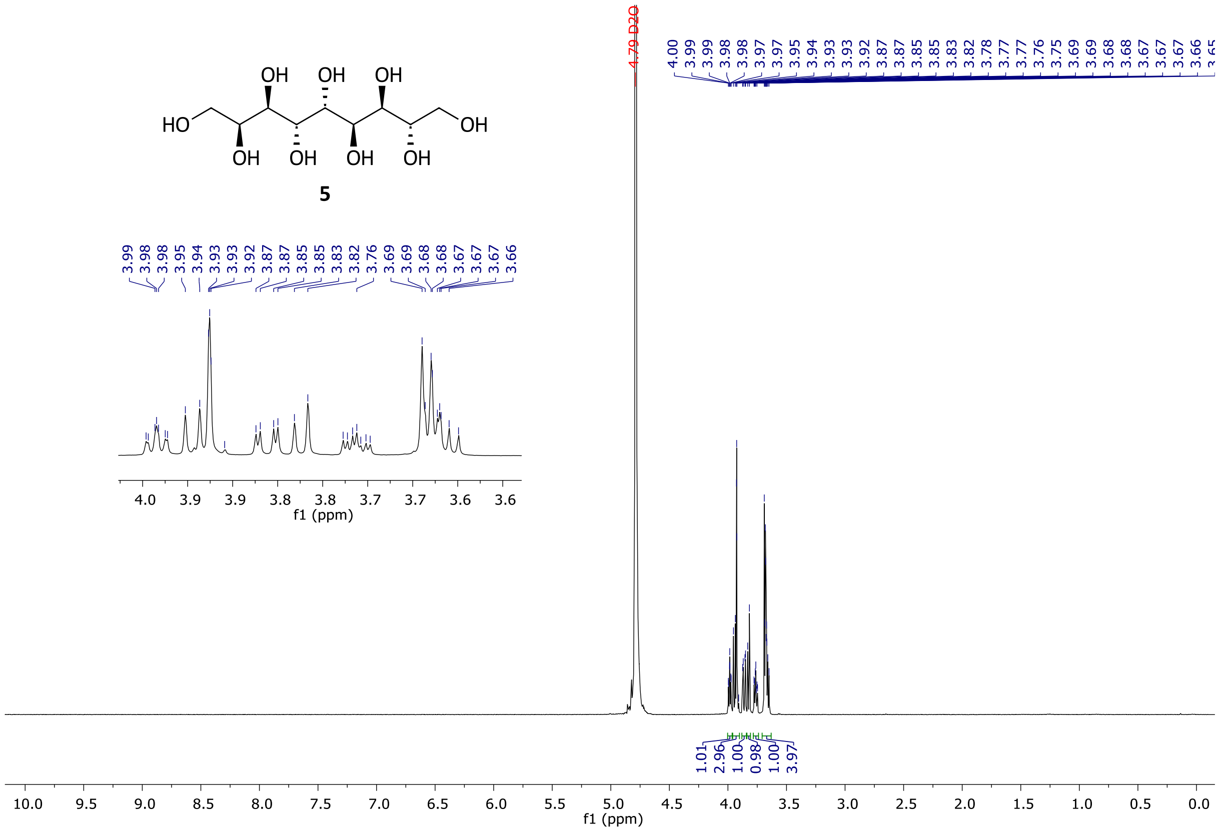

Figure S48. ^{1}H-NMR (600 MHz, D_2O) of L-*lyxo*-L-*manno*-nonitol (**5**).

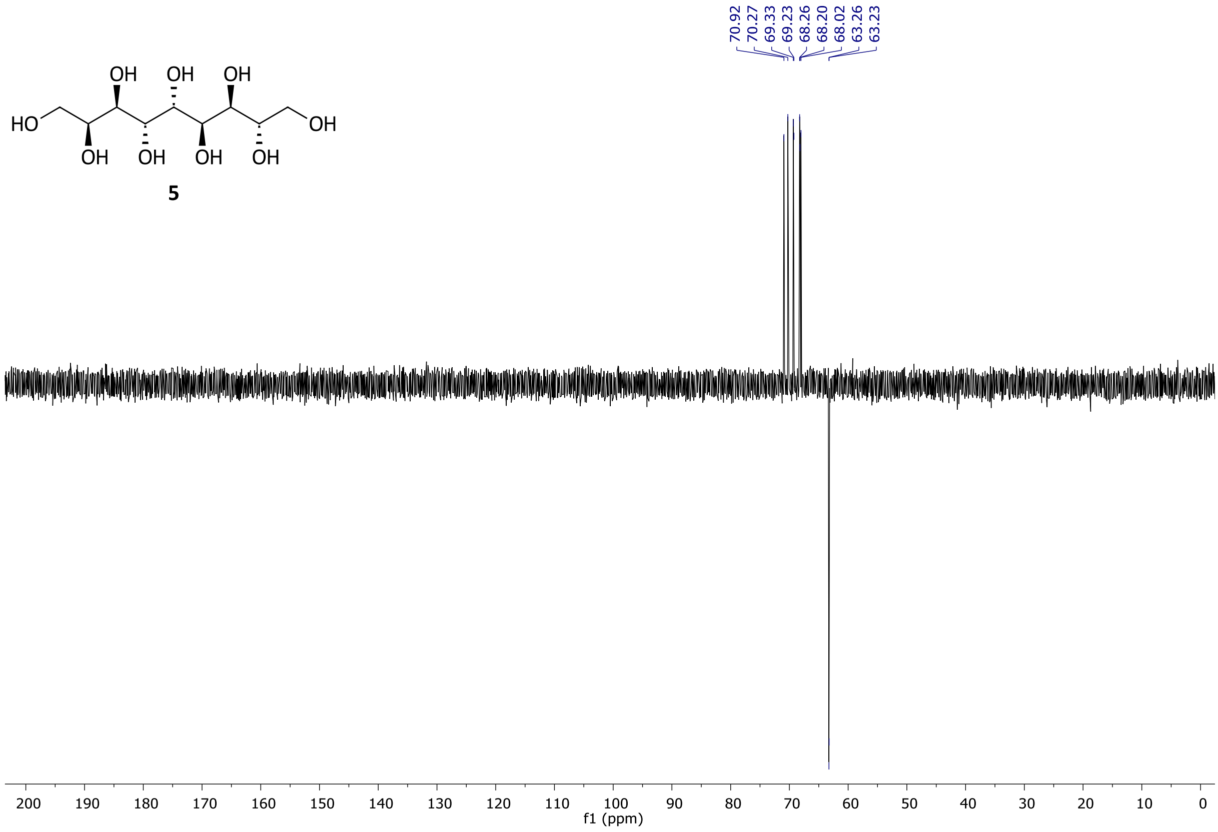

Figure S49. ^{13}C{^{1}H}-NMR (151 MHz, D_2O) of L-*lyxo*-L-*manno*-nonitol (**5**).

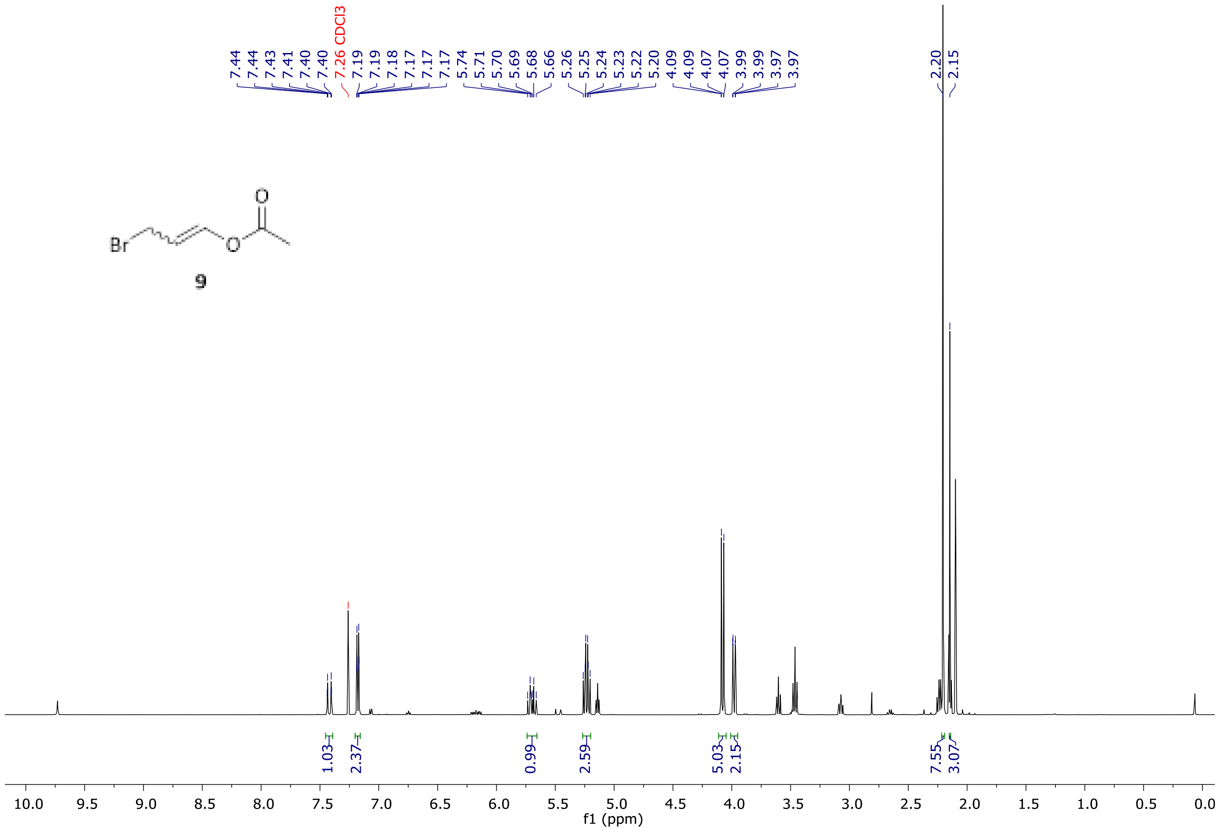

Figure S50. ^{1}H-NMR (400 MHz, $CDCl_3$) of 3-bromoprop-1-en-1-yl acetate (**9**) ((*E*)/(*Z*)-isomer mixture).

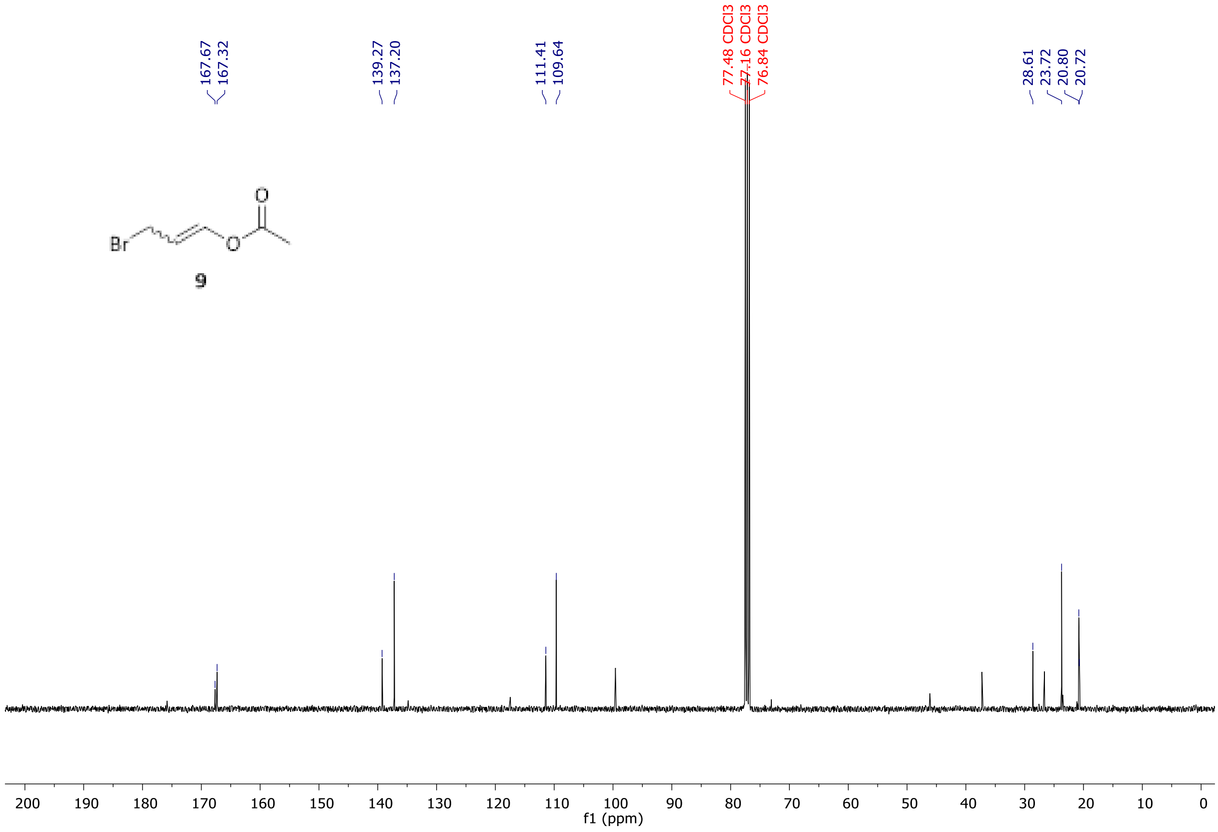

Figure S51. $^{13}C\{^{1}H\}$-NMR (101 MHz, $CDCl_3$) of 3-bromoprop-1-en-1-yl acetate (**9**) ((*E*)/(*Z*)-isomer mixture).

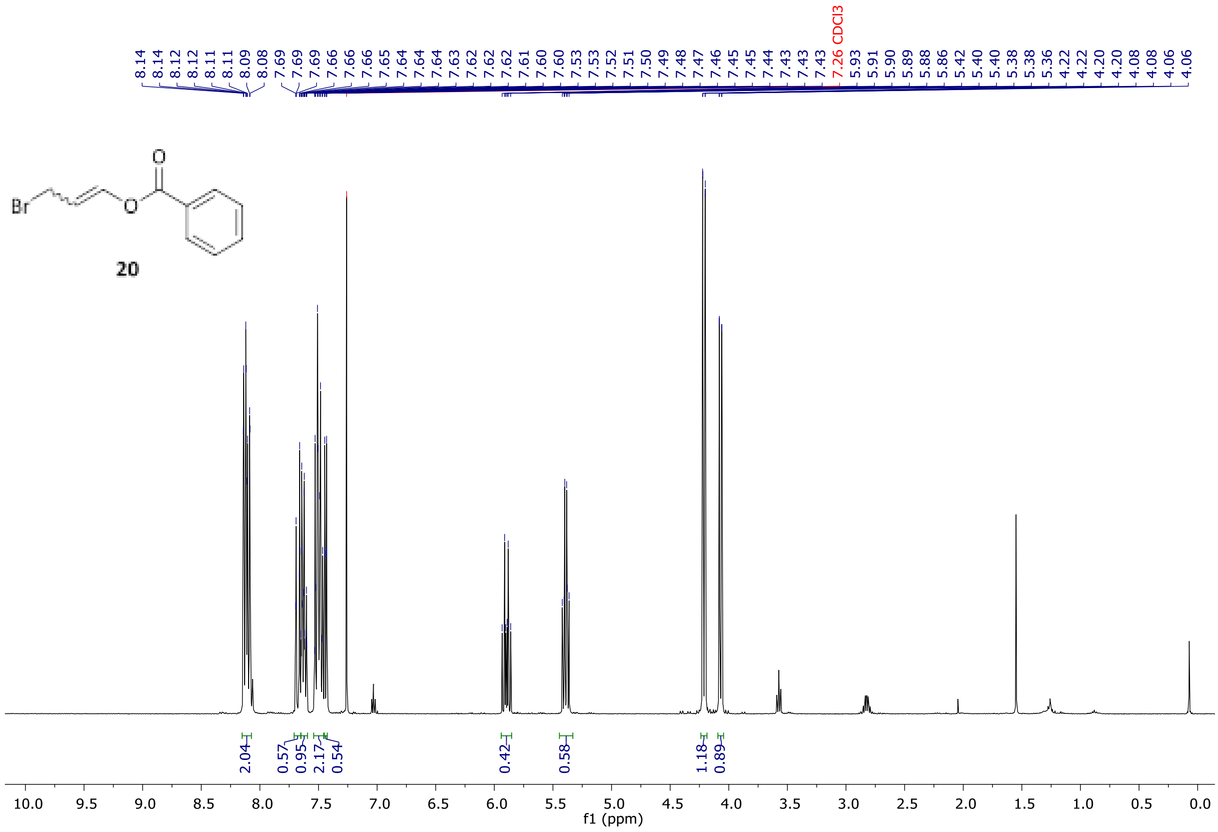

Figure S52. ^{1}H-NMR (400 MHz, $CDCl_3$) of 3-bromoprop-1-en-1-yl benzoate (**20**) ((*E*)/(*Z*)-isomer mixture).

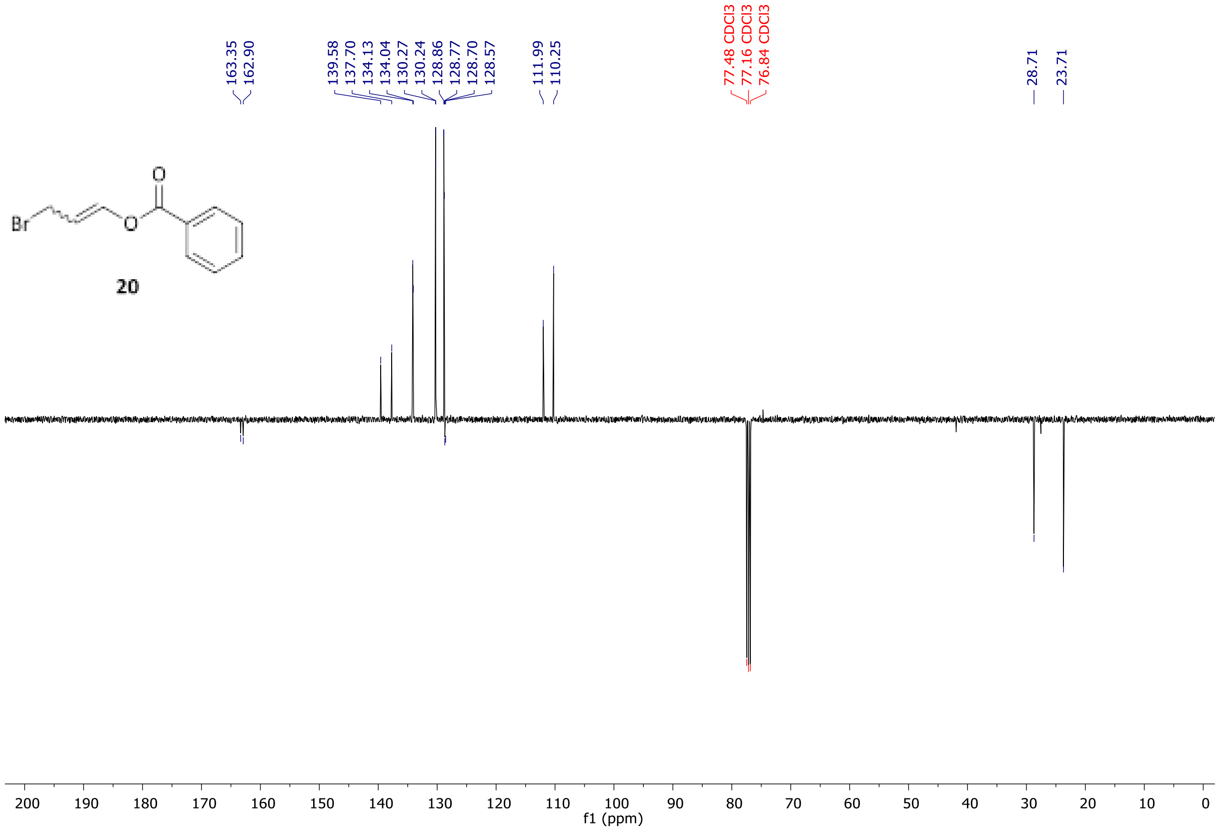

Figure S53. $^{13}C\{^{1}H\}$-NMR (101 MHz, $CDCl_3$) of 3-bromoprop-1-en-1-yl benzoate (**20**) ((*E*)/(*Z*)-isomer mixture).

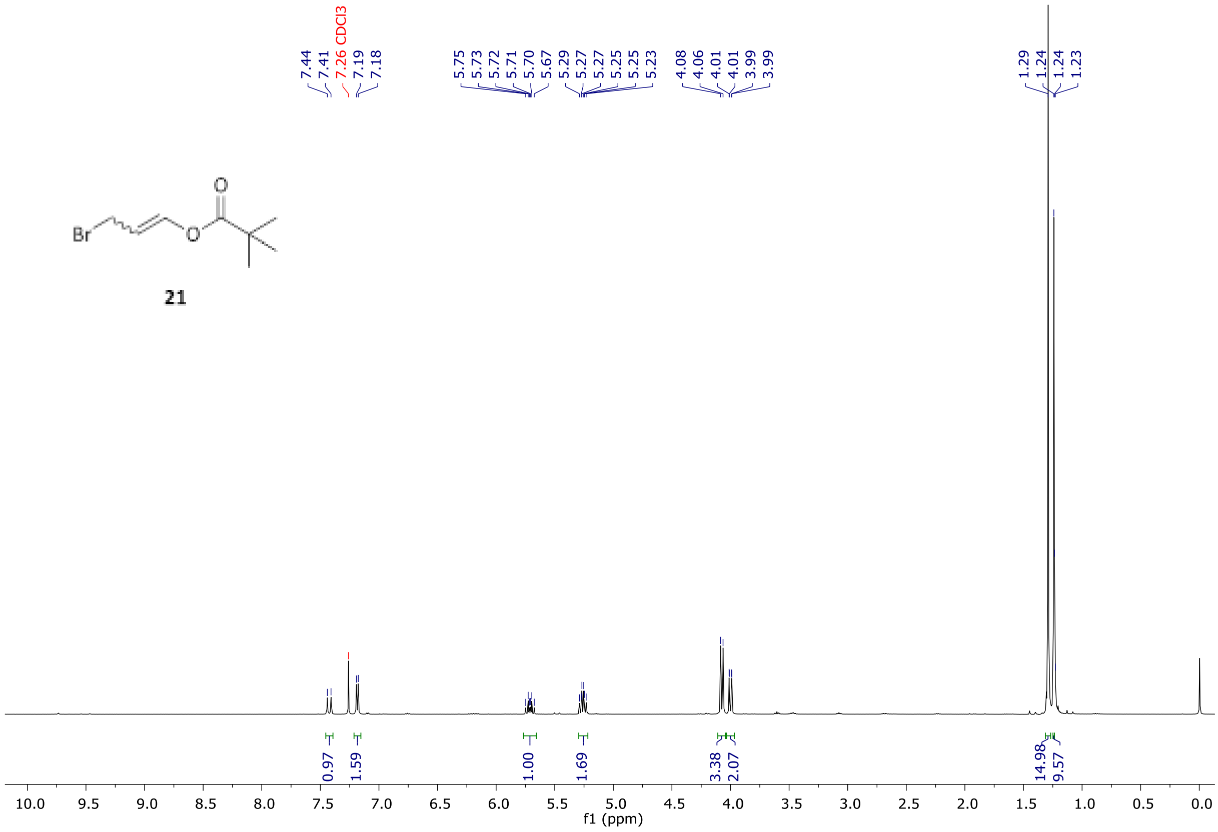

Figure S54. ^{1}H-NMR (400 MHz, $CDCl_3$) of 3-bromoprop-1-en-1-yl pivalate (**21**) ((*E*)/(*Z*)-isomer mixture).

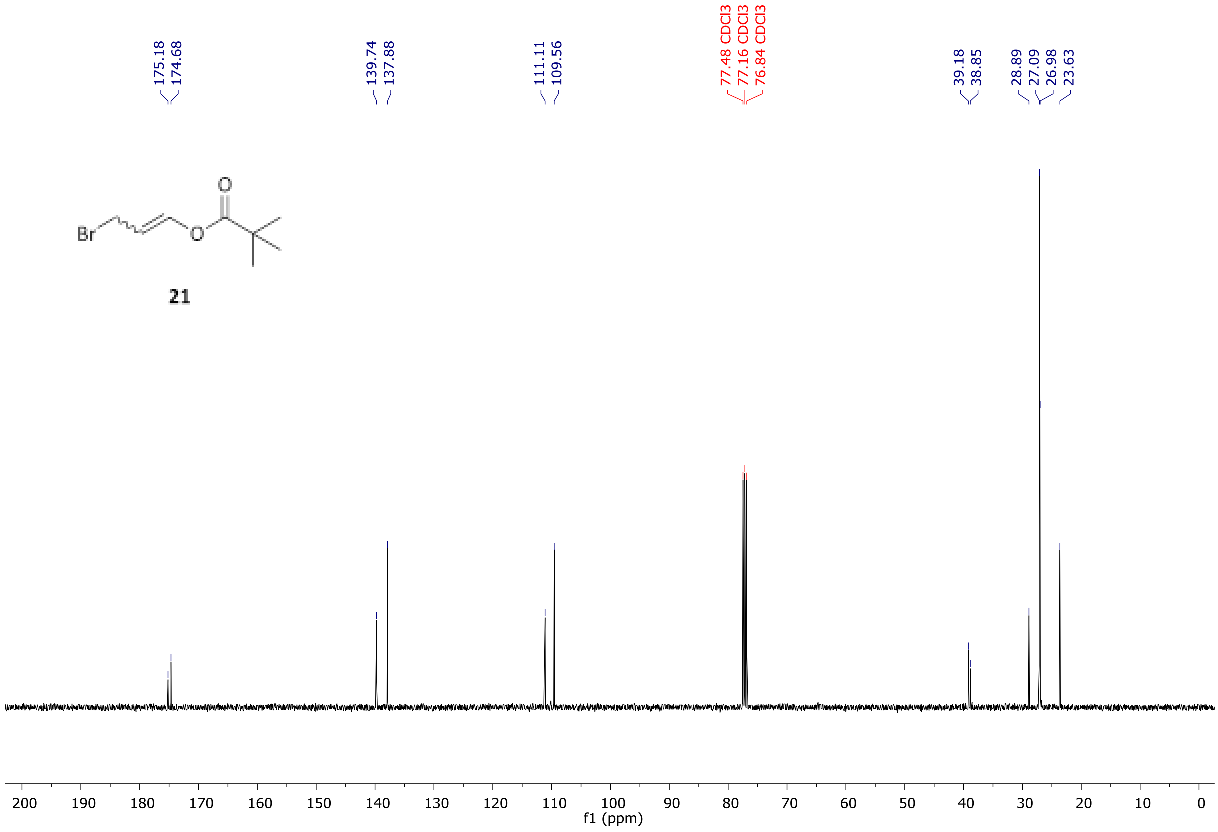

Figure S55. $^{13}C\{^{1}H\}$-NMR (101 MHz, $CDCl_3$) of 3-bromoprop-1-en-1-yl pivalate (**21**) ((*E*)/(*Z*)-isomer mixture).

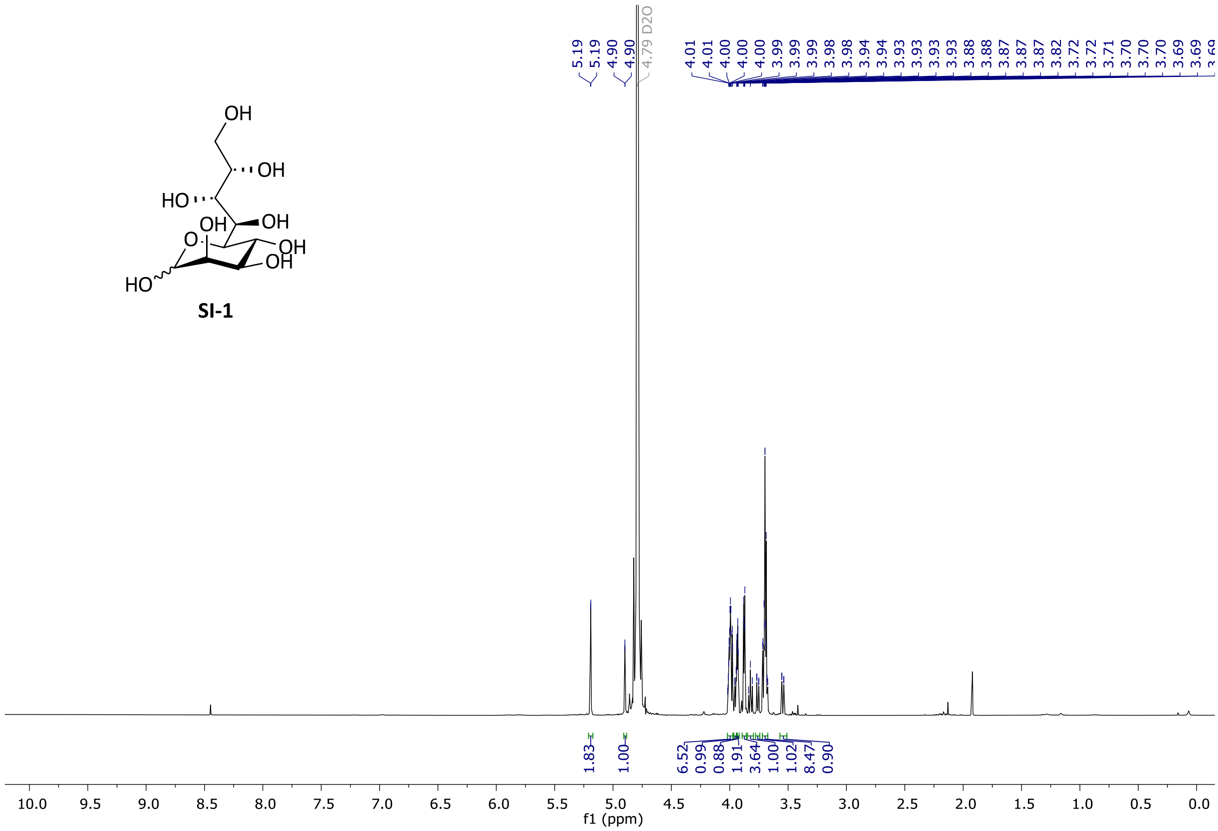

Figure S56. ^{1}H-NMR (600 MHz, D_2O) of L-*lyxo*-L-*manno*-nonose (**SI-1**).

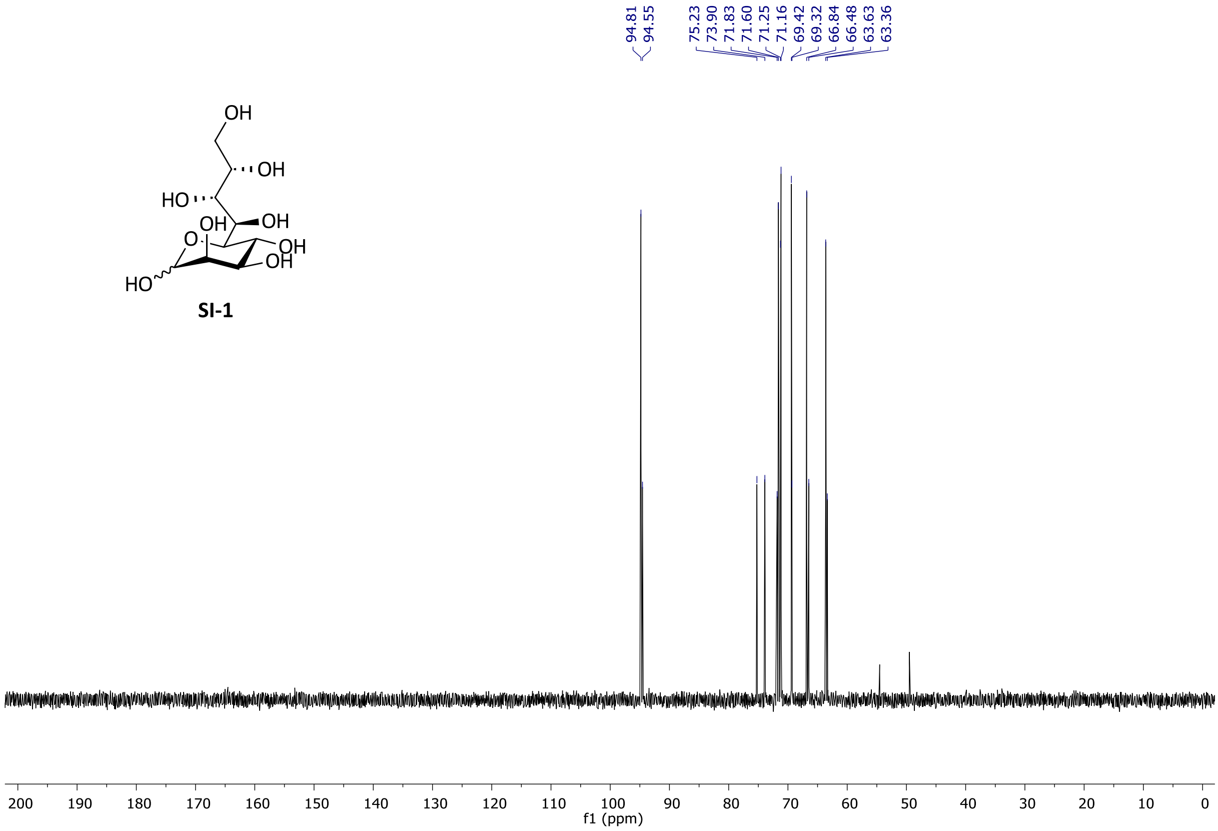

Figure S57. $^{13}C\{^{1}H\}$-NMR (101 MHz, D_2O) of L-*lyxo*-L-*manno*-nonose (**SI-1**).

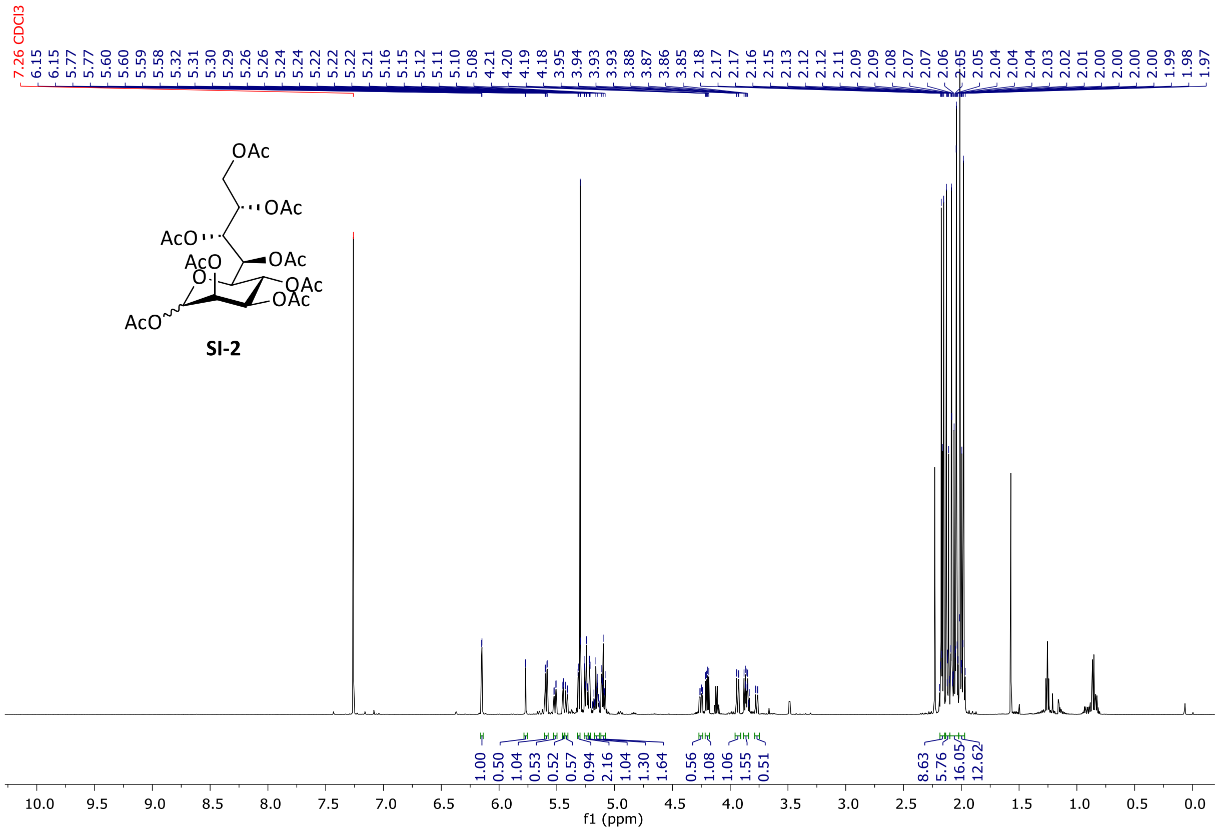

Figure S58. ^{1}H-NMR (600 MHz, $CDCl_3$) of 1,2,3,4,6,7,8,9-octa-*O*-acetyl-L-*lyxo*-L-*manno*-nonose (**SI-2**).

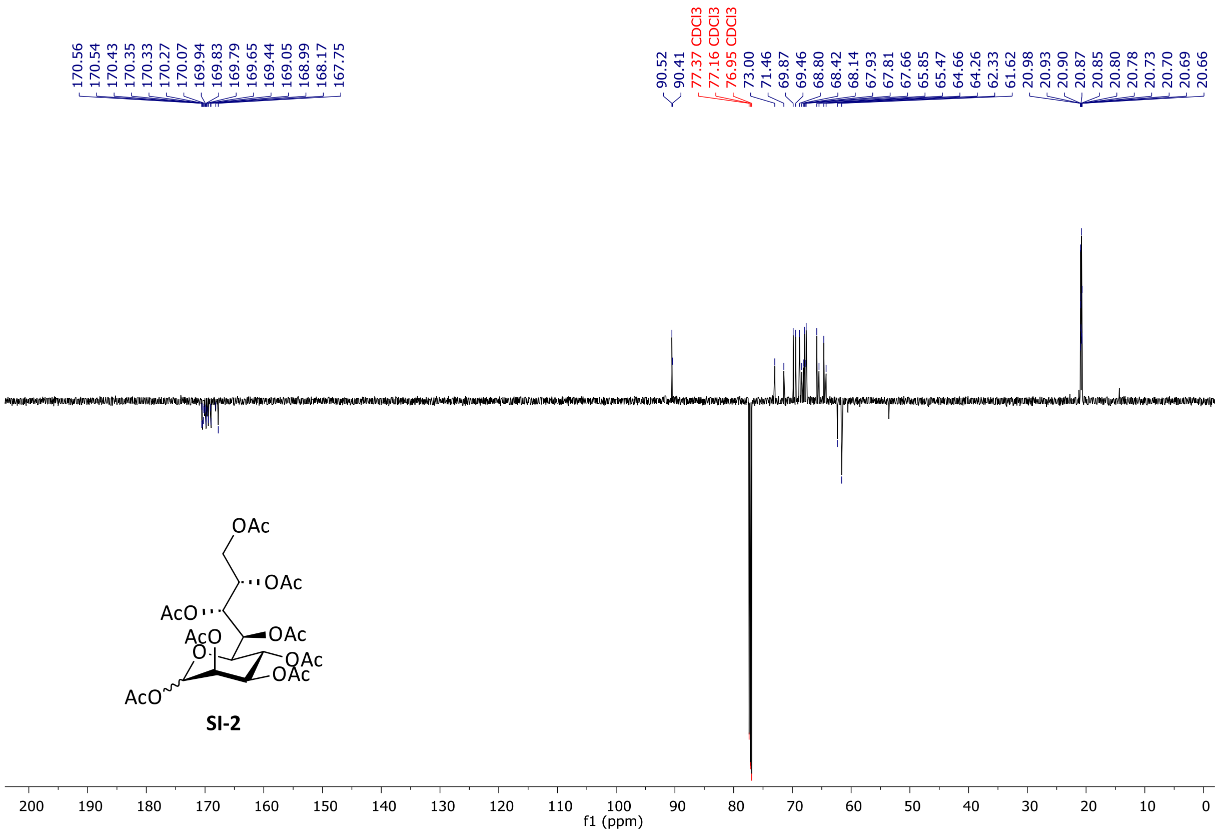

Figure S59. $^{13}C\{^1H\}$-NMR (151 MHz, $CDCl_3$) of 1,2,3,4,6,7,8,9-octa-*O*-acetyl-L-*lyxo*-L-*manno*-nonose (**SI-2**).

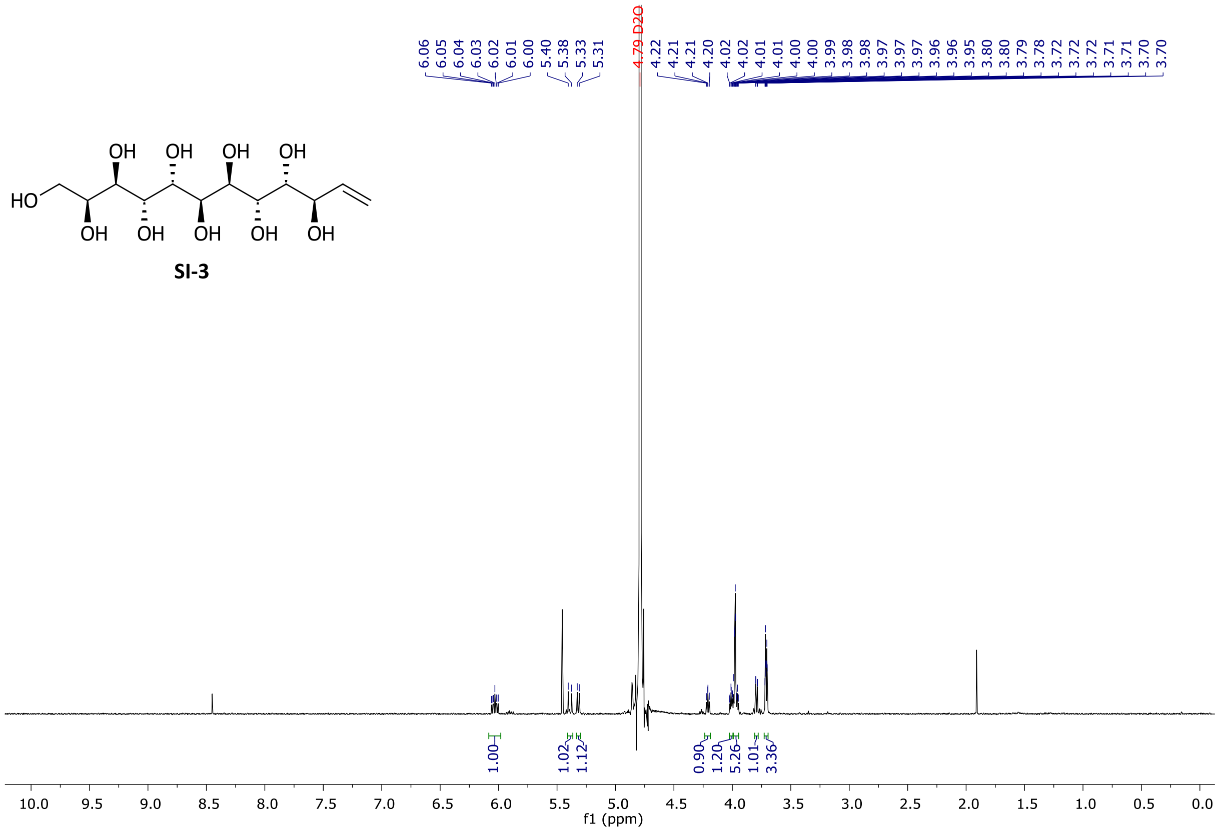

Figure S60. ^{1}H-NMR (600 MHz, D_2O) of 1,2-dideoxy-L-*glycero*-D-*manno*-D-*manno*-dodecenitol (**SI-3**).

References

(1) Stanetty, C.; Baxendale, I. R. Large-scale synthesis of crystalline 1,2,3,4,6,7-hexa-*O*-acetyl-L-*glycero*-α-D-*manno*-heptopyranose. *Eur. J. Org. Chem.* **2015**, *2015*(12), 2718-2726. DOI: 10.1002/ejoc.201500024

(2) Lombardo, M.; Morganti, S.; Trombini, C. 3-Bromopropenyl esters in organic synthesis: Indium- and zinc-mediated entries to alk-1-ene-3,4-diols. *J. Org. Chem.* **2003**, *68*(3), 997-1006. DOI: 10.1021/jo0262457

(3) Draskovits, M.; Biedermann, N.; Schnürch, M.; Stanetty, C. The synthesis of higher-carbon sugar alcohols via indium-mediated acyloxyallylation as potential phase change materials. *Monatsh. Chem.* **2023**. DOI: 10.1007/s00706-023-03136-6

(4) Matveeva, E. D.; Podrugina, T. A.; Sandakova, N. G.; Zefirov, N. S. Triphenylphosphine-2,4,4,6-tetrabromo-2,5-cyclohexadienone complex as a reagent for preparation of carboxylic acid bromides. *Russ. J. Org. Chem.* **2004**, *40*(10), 1469-1472. DOI: 10.1007/s11178-005-0042-0

(5) Draskovits, M.; Stanetty, C.; Baxendale, I. R.; Mihovilovic, M. D. Indium- and zinc-mediated acyloxyallylation of protected and unprotected aldotetroses—Revealing a pronounced diastereodivergence and a fundamental difference in the performance of the mediating metal. *J. Org. Chem.* **2018**, *83*(5), 2647-2659. DOI: 10.1021/acs.joc.7b03063

(6) Kalaus, H.; Reichetseder, A.; Scheibelreiter, V.; Rudroff, F.; Stanetty, C.; Mihovilovic, M. D. A kinetic photometric assay for the quantification of the open-chain content of aldoses. *Eur. J. Org. Chem.* **2021**, *2021*(18), 2589-2593. DOI: 10.1002/ejoc.202001641
